# Strategic Timing of Gene Silencing: Cellular Kinetics‐Based Administration of siRNA for Optimized Photothermal Cancer Treatment

**DOI:** 10.1002/advs.202510802

**Published:** 2025-09-06

**Authors:** Tianliang Fang, Li Li, Ziyad Tariq Muhseen, Lucas A. Lane, Huiming Cai, Christopher J. Butch, Yiqing Wang

**Affiliations:** ^1^ Department of Biomedical Engineering College of Engineering and Applied Sciences State Key Laboratory of Analytical Chemistry for Life Science Nanjing University Nanjing 210023 China; ^2^ International Ph.D. Program in Biomedical Engineering College of Biomedical Engineering Taipei Medical University Taipei 11031 Taiwan, ROC; ^3^ Graduate Institute of Biomedical Optomechatronics College of Biomedical Engineering Taipei Medical University Taipei 11031 Taiwan, ROC; ^4^ Nanjing Nuoyuan Medical Devices Co. Ltd Nanjing 211514 China

**Keywords:** biomimetic liposome, dual‐phase therapy, mild photothermal therapy, siRNA

## Abstract

Heat shock protein 70 (HSP70) represents a critical barrier to effective mild‐temperature photothermal therapy (MPTT), limiting its clinical utility in aggressive cancers like triple‐negative breast cancer (TNBC). While small interfering RNA (siRNA)‐mediated HSP70 suppression offers a promising solution, optimal timing for this therapeutic combination remains unexplored. Here, it is demonstrated that precisely timed administration significantly enhances MPTT efficacy through systematic temporal characterization of HSP70 expression dynamics. A three‐component temperature‐sensitive hybrid nanocarrier (I‐sR@MLNP) is developed that integrates: 1) indocyanine green dimer (ICG‐II) with exceptional photothermal conversion efficiency (PTCE, 95.4%); 2) macrophage membrane‐derived lipid nanoparticles for active TNBC targeting through integrin α4/vascular cell adhesion molecule‐1 (VCAM‐1) axis; and 3) HSP70‐targeting siRNA to overcome thermo‐resistance. This multifunctional platform enables spatiotemporally controlled co‐delivery and photo‐triggered release of both therapeutic agents. Through comprehensive profiling of post‐release HSP70 mRNA and protein kinetics, a critical therapeutic window is identified at 36 h post‐initial treatment when siRNA‐mediated suppression maximally sensitized cancer cells to subsequent thermal stress. In mouse TNBC models, this temporally optimized two‐phase MPTT approach achieves superior tumor reduction compared to conventional single‐treatment (+87%) or non‐optimized protocols (+43%). The findings establish a novel time modulated framework for enhancing nanomedicine efficacy by aligning treatment scheduling with underlying molecular kinetics—a strategy with potential applications across various siRNA‐based cancer therapies where timing of intervention may significantly impact therapeutic outcomes.

## Graphical abstract

Coordinating therapy with gene expression kinetics emerges as a key strategy for maximizing nanomedicine efficacy in aggressive cancers. A hybrid nanocarrier delivering HSP70‐targeting siRNA demonstrates this chronotherapeutic principle through systematic cellular profiling. Identification of a critical 36‐hour therapeutic window enables dual‐phase treatment achieving 86% enhanced tumor suppression compared to conventional protocols, establishing kinetics‐based nanomedicine design.

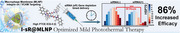

## 1 Introduction

Heat shock proteins (HSPs), particularly HSP70, are critical cellular defenses against thermal stress and pose a significant barrier to the clinical effectiveness of photothermal therapy (PTT) in cancer treatment, especially MPTT.^[^ ^1^ ^]^ PTT is an emerging cancer treatment modality that converts light energy into localized heat using photothermal agents (PTAs), offering minimal invasiveness and low systemic toxicity.^[^ ^2^ ^]^ These qualities make PTT an attractive option for direct treatment of surface malignancies such as skin and esophageal cancers,^[^ ^3^ ^]^ as well as some refractory or surgically inaccessible tumors through insertion of fiberoptic elements.^[^ ^4^ ^]^ To bypass HSP and other cellular defenses against heat shock, conventional PTT strategies typically employ temperatures above 50 °C to overcome HSP‐mediated resistance. While these high temperatures enhance treatment efficacy, precise control over which tissues are damaged is difficult to achieve.^[^ ^5^ ^]^ Consequently, side effects of PTT often include pain, vascular occlusion that can complicate future treatments, and in some cases, permanent damage.^[^ ^6^ ^]^

To address these limitations, MPTT using lower, sustained heating to 42–47 °C has attracted significant research interest due to its reduced side‐effect profile.^[^ ^7^ ^]^ However, this reduction in temperature often diminishes tumor‐killing efficacy. At these milder temperatures, cells have sufficient time to rapidly express HSP70, which maintains protein function and prevents apoptosis, thereby conferring resistance to heat‐based therapies. Thus, successful MPTT requires adjuvant strategies that effectively suppress HSP‐mediated thermo‐resistance. The two primary adjuvant approaches are coadministration of small molecule inhibitors to reduce the protective effect of HSP70 and either co‐ or prior administration of siRNA that target HSP70 mRNA and prevent expression of the protein.^[^ ^8^ ^]^

siRNA‐mediated gene silencing is an attractive option for enhancing MPTT due to higher specificity and the potential for increased activity, but faces challenges to optimal use in delivery and kinetics of action.^[^ ^2^, ^9^ ^]^ Use of siRNA typically requires a liposomal formulation capable of selectively delivering siRNA to the targeted cancer cells^[^ ^10^ ^]^ and faces several additional kinetic challenges: siRNA molecules degrade rapidly, baseline intracellular HSP70 must first be depleted, and thermal stress triggers compensatory transcriptional upregulation of HSP70, complicating treatment dynamics.^[^ ^11^ ^]^ Although previous studies have demonstrated siRNA's potential to sensitize tumors to PTT,^[^ ^2^, ^12^ ^]^ optimizing the timing and dosing schedule to achieve maximal therapeutic efficacy remains a significant barrier to clinical implementation.^[^ ^11a^ ^]^

To overcome these siRNA kinetic challenges and establish an evidence‐based treatment schedule, we conducted a systematic temporal analysis of HSP70 expression dynamics in TNBC cells following thermal stress and siRNA delivery. TNBC represents the most aggressive breast cancer subtype, disproportionately accounting for 30–40% of breast cancer‐related deaths despite comprising only 10–20% of cases, thus presenting an urgent clinical need.^[^ ^13^ ^]^ Our experimental strategy for identifying the optimal therapeutic window consists of a two‐dose, two‐treatment protocol: the first dose releases the PTA and siRNA through lysosomal disruption under 785 nm laser irradiation,^[^ ^14^ ^]^ and the second dose, administered at variable intervals, demonstrates the importance of HSP70 suppression dynamics on therapeutic efficacy. By precisely mapping the window during which siRNA‐mediated suppression most effectively counteracts heat‐induced HSP70 upregulation, we define an optimal therapeutic interval for sequenced MPTT treatment.

**Scheme 1:**
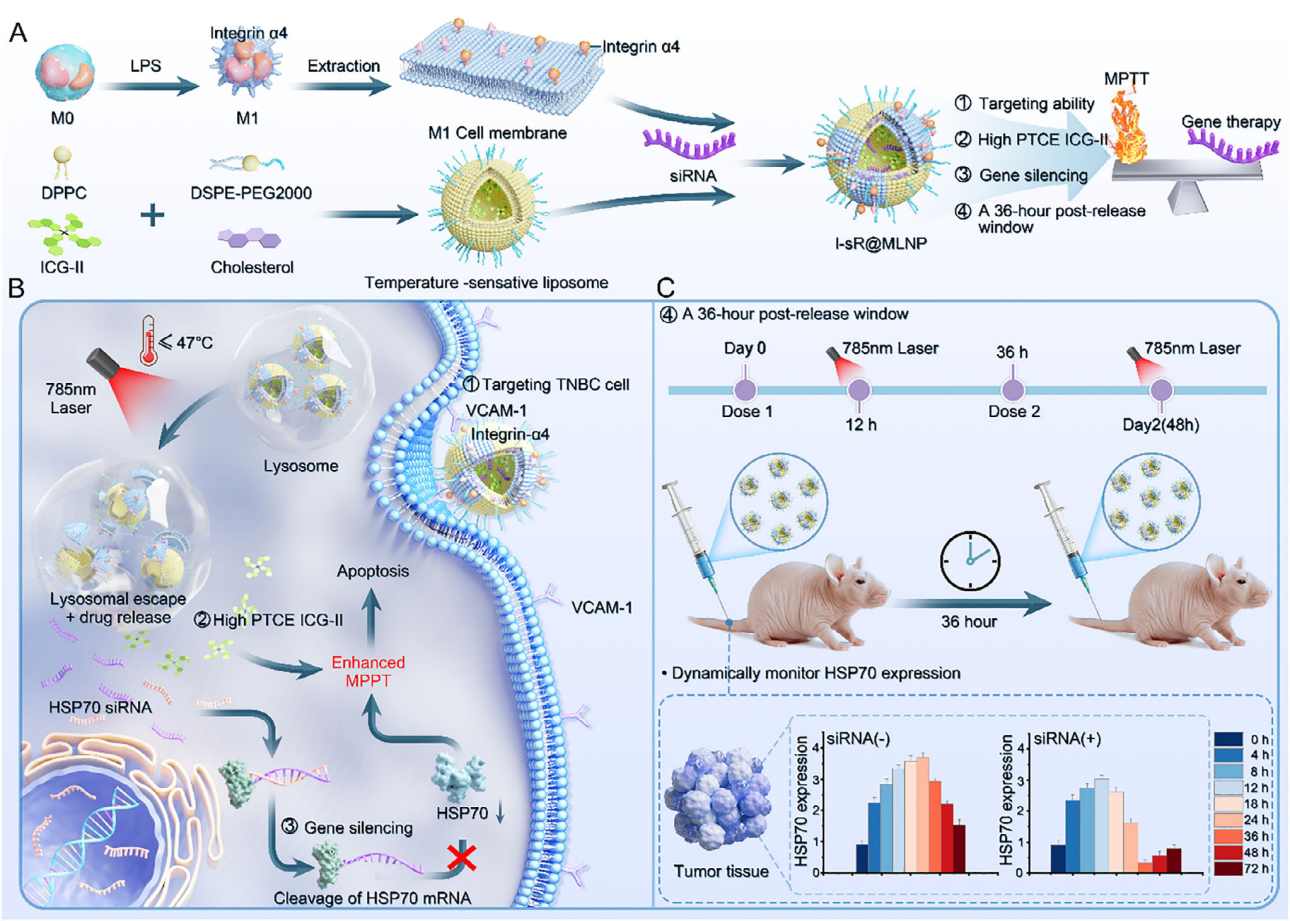
The synthesis of a three‐component temperature‐sensitive hybridized nanocarrier (I‐sR@MLNP) and its mechanism for eliciting antitumor responses. A) The I‐sR@MLNP was synthesized through sequential integration of biological and synthetic components. M1 cell membranes were first isolated from M0 polarized with lipopolysaccharide (LPS). Temperature‐sensitive liposomes were prepared via lipid film hydration using a composite of dipalmitoylphosphatidylcholine (DPPC), 1,2‐distearoyl‐sn‐glycero‐3‐phosphoethanolamine‐polyethylene glycol 2000 (DSPE‐PEG2000), cholesterol, and indocyanine green II (ICG‐II). The final nano‐construct was assembled by co‐encapsulating small interfering RNA (siRNA) with hybridized nanoparticles derived from M1 cell membrane and temperature‐sensitive liposome. B) Upon subcutaneous administration, I‐sR@MLNP selectively targeted TNBC cells via integrin α4. Near‐infrared irradiation (785 nm) triggered photothermal activation of the temperature‐sensitive liposomal matrix, inducing simultaneous payload release and lysosomal escape. This spatiotemporally controlled process facilitates cytosolic delivery of siRNA, enabling RNA interference‐mediated cleavage of HSP70 mRNA. C) A chronotherapeutic strategy was implemented to align siRNA bioactivity with tumor microenvironment dynamics.

To this end, we developed a three‐component temperature‐sensitive hybrid nanocarrier (I‐sR@MLNP, **Scheme** **1**) for the targeted co‐delivery of the indocyanine green dimer, ICG‐II (I), and siRNA (sR) in a hybrid macrophage membrane/lipid nanoparticle (MLNP). This combination provides mRNA silencing along with the high PTCE of ICG‐II (95.4%)^[^ ^7^ ^]^ while the macrophage membrane coating confers active tumor‐targeting capability through integrin α4 /VCAM‐1 complexation, enabling preferential accumulation and lysosomal uptake in TNBC cells.^[^ ^15^ ^]^ Through quantitative real‐time polymerase chain reaction (qPCR) and Western blot (WB) analyses, we systematically profiled HSP70 mRNA and protein expression dynamics post‐initial treatment, determining a 36 h post‐treatment interval as optimal for maximal siRNA‐induced HSP70 suppression. Both in vitro and in vivo validation confirmed that administering the second photothermal dose at this 36 h time point significantly enhanced cancer cell sensitivity and therapeutic outcomes.

Collectively, this work demonstrates the efficacy of the tumor‐targeted hybrid liposomal nanocarrier I‐sR@MLNP as a highly effective PTA and introduces a robust timing‐optimized strategy for leveraging siRNA‐based gene silencing in combination with MPTT. Our findings provide a significant step toward overcoming thermo‐resistance barriers, offering a promising translational approach for enhanced RNA interference‐guided cancer therapies.

## 2 Results

### 2.1 Preparation and Characterization of I‐sR@MLNP

ICG‐II was synthesized as detailed in the referenced article. Nanoparticles were formulated using the thin film hydration method. Due to the aggregation state of ICG‐II affects its PTCE, we processed liposomes loaded with different aggregation states of ICG‐II under two reaction conditions of 4 and 40 °C, respectively. The dispersion state exhibits an optimal absorption peak at 785 nm at 4 °C, whereas the J‐aggregation state at 40 °C shows an optimal absorption peak at 890 nm (Figure S1A, Supporting Information). This observation suggests that nanoparticles with a dispersed state of ICG‐II (I@LNP) are readily formed at 4 °C. Conversely, ICG‐II forms J aggregates within the nanoparticles at 40 °C, and we designated this nanoparticle as I(J)@LNP. I(J)@LNP was generated with an 8 h reaction period at 40 °C to ensure the complete formation of J aggregates, as depicted in Figure S1B (Supporting Information). Both I@LNP and I(J)@LNP manifest as electrically neutral spherical structures with an average particle size of ≈120 nm (Figure S1C–J, Supporting Information). The dispersion coefficients for both nanoparticles fall within the range of about 0.15–0.20. The encapsulation efficiencies (EE) for I@LNP and I(J)@LNP were determined to be 60.2 ± 3.8% and 82.4 ± 4.2%, respectively (Figure S2, Supporting Information). We explored the photothermal properties of liposomes (I@LNP and I(J)@LNP) in different states of ICG‐II. As shown in Figure S6 (Supporting Information), the photothermal properties and PTCE (95.4%) of ICG‐II and I@LNP containing the same concentration of ICG‐II were the same and superior to that of I(J)@LNP (89.6%). Following a comprehensive assessment of both nanoparticle types, we selected 4 °C as the optimal preparation condition for nanoparticles. This condition also ensured the preservation of siRNA integrity during the encapsulation process.

**Figure 1:**
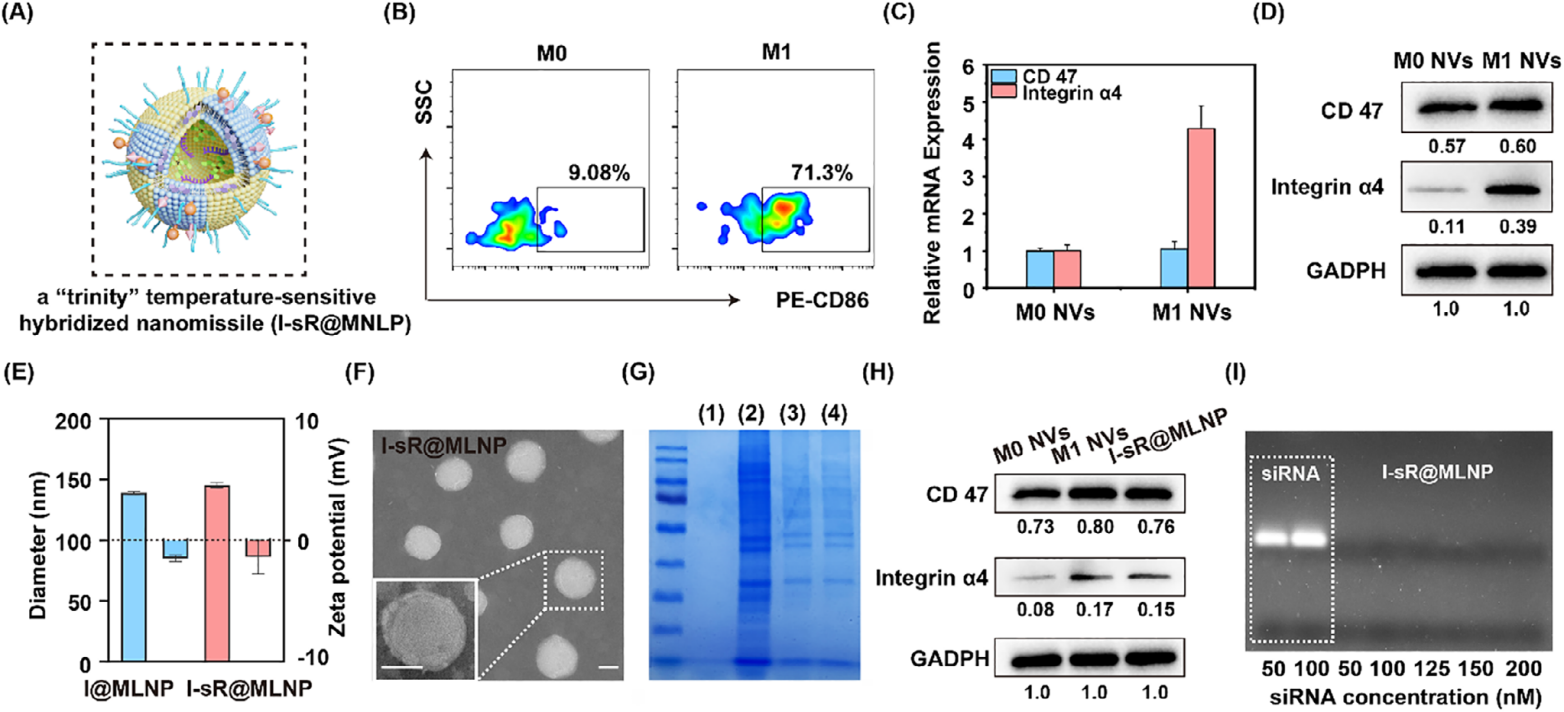
Preparation and characterization of I‐sR@MLNP. A) The synthesis of I‐sR@MLNP; B) The proportion of M1 macrophages was analyzed by flow cytometry; B) The relative integrin α4 and CD47 mRNA levels in M0 NVs and M1 NVs; C) The qPCR analysis of the integrin α4 and CD47 protein expression level in M0 NVs and M1 NVs (*n = 3*); D) The WB analysis of the integrin α4 and CD47 protein expression level in M0 NVs and M1 NVs; E) The diameters and zeta potentials of I@MLNP and I‐sR@MLNP; F) The transmission electron microscope (TEM) data of I‐sR@MLNP (*n = 3*). Scale bar: 50 nm; G) Protein profiles determined by sodium dodecyl sulfate‐polyacrylamide gel electrophoresis (SDS‐PAGE) electrophoresis assay. H) The WB analysis of the integrin α4 and CD47 protein expression level in different materials. I) Verify whether I‐sR@MLNP containing different concentrations of siRNA was leaked by gel electrophoresis. Notes: 1 means I‐sR@LNP; 2 means M1 NVs; 3 means I@MLNP; 4 means I‐sR@MLNP;.

Previous reports demonstrate that macrophage cell membranes containing integrin α4 exhibit specific binding affinity to MDA‐MB‐231 cells overexpressing VCAM‐1.^[^ ^16^ ^]^ Building on this targeting mechanism, we developed macrophage membrane‐hybridized nanoparticles to enhance the targeting ability of nanomaterials for TNBC (**Figure** **1**A). Given the differential expression profiles of integrin α4 among macrophage subtypes, classical M1 polarization was induced using lipopolysaccharide (LPS), with flow cytometric analysis confirming 71.3% M1 polarization efficiency (Figure 1B). Subsequently, the analysis of integrin α4 expression between M0‐ and M1‐derived nanovesicles (M0 NVs vs M1 NVs) was performed using qPCR and WB. Consistent with previous reports,^[^ ^17^ ^]^ membrane‐associated integrin α4 levels in M1 NVs significantly exceeded those in M0 NVs (Figure 1C,D). Notably, despite CD47's well‐established role as an immunoregulatory transmembrane protein captured by the immune system, WB analysis revealed comparable CD47 expression between the two nanovesicle groups. Based on these findings, we engineered M1 NV‐hybridized liposome co‐encapsulating ICG‐II and siRNA for the treatment of MPTT. Subsequently, I‐sR@MLNP was synthesized and characterized. In parallel, nanoparticles without siRNA (I@MLNP) were also prepared using the same method to serve as a control for subsequent experiments. Dynamic light scattering demonstrated both I@MLNP and I‐sR@MLNP with an average diameter of 140 nm and a polydispersity index (PDI) of 0.18 ± 0.02 (Figure 1E; Figure S3, Supporting Information). The TEM data further confirmed that I‐sR@MLNP took the form of a round sphere with a double membrane and uniform size (Figure 1F). Collectively, these characterizations underscored I‐sR@MLNP had the uniform and excellent nano‐size.

Functional validation of the successful integration of M1 NVs onto both I@MLNP and I‐sR@MLNP, we examined the protein profiles. The coomassie brilliant blue staining confirmed preservation of the full membrane proteome in both nanoparticle formulations (Figure 1G). To assess the hybrid liposomes inheriting natural protein‐mediated immune escape and tumor targeting from macrophage, WB analysis was employed, which unequivocally identified critical biomarkers (CD47 and integrin α4) in I‐sR@MLNP (Figure 1H). Spectrophotometric characterization via UV–vis spectroscopy revealed characteristic absorption peaks at ≈785 nm for both liposomal systems (Figure S4, Supporting Information), indicative of ICG‐II encapsulated in a dispersed state with a drug loading efficiency of ≈43% in both formulations. For systematic evaluation of nucleic acid containment, a siRNA dose‐escalation encapsulation assay was conducted. Gel electrophoresis analysis confirmed complete siRNA retention at 200 nm payload concentrations without detectable nucleic acid leakage (Figure 1I), demonstrating superior payload containment capacity of the hybrid nanocarriers. Collectively, the above results demonstrated that I‐sR@MLNP is a nanomaterial with potential ultra‐high PTCE and potential targeting ability.

### 2.2 Photothermal Property and Temperature‐Sensitive Responsiveness of I‐sR@MLNP

**Figure 2:**
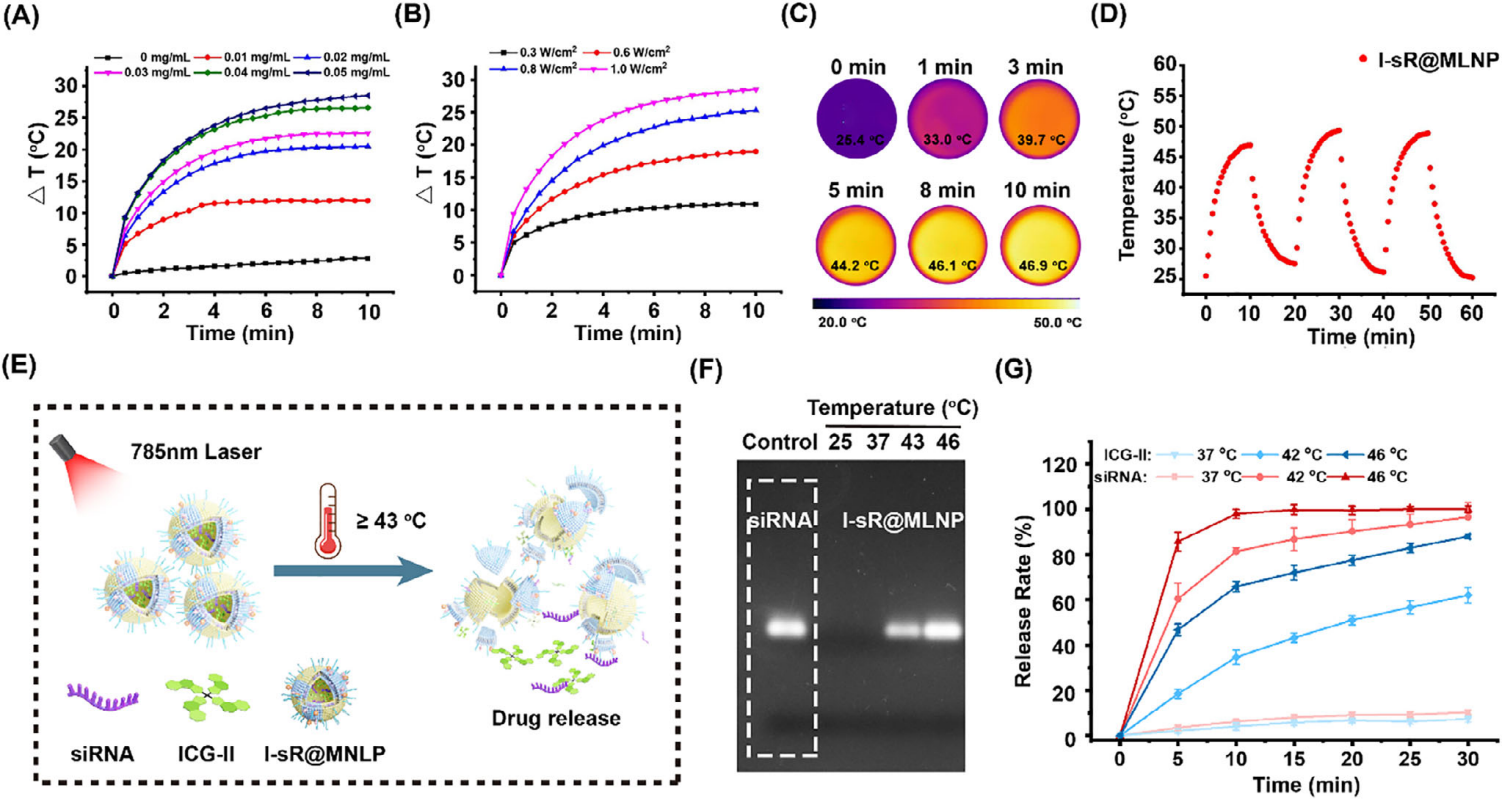
Photothermal property and temperature‐sensitive responsiveness of I‐sR@MLNP. A) The temperature changes (△T) of I‐sR@MLNP with different concentrations under laser (785 nm, 1 W cm^−2^); B) The △T of I‐sR@MLNP (including 50 µg mL^−1^ ICG‐II) under laser with different power densities; C) The IR thermal images of I‐sR@MLNP (785 nm, 0.6 W cm^−2^); D) The temperature of I‐sR@MLNP over several ON/OFF cycles involving irradiation with a laser (785 nm, 1 W/cm^2^) for 60 min followed by passive cooling. E) The synthesis of temperature‐sensitive responsiveness of I‐sR@MLNP; F) Temperature‐triggered release profiles of siRNA and ICG‐II from I‐sR@MLNP at 37, 42, and 46 °C, respectively. G) Temperature‐triggered release profiles of siRNA from I‐sR@MLNP by gel electrophoresis (*n = 3*). Note:The red line represents the release rates of siRNA. The blue line represents the release rates of ICG‐II.

Systematic evaluation of I‐sR@MLNP's photothermal performance was conducted using a calibrated thermocouple probe coupled with infrared (IR) thermography. As demonstrated in **Figure** **2**A,B, the nanoplatform exhibited concentration‐dependent and power density‐dependent temperature elevation. The IR thermography images visually substantiated the exceptional photothermal properties of I‐sR@MLNP (Figure 2C; Figures S8–S11, Supporting Information). We also assessed the photothermal stability of the hybrid liposomes. As depicted in Figure 2D and I‐sR@MLNP demonstrated outstanding photostability even after undergoing three photo‐switching cycles. Meanwhile, we compared the photothermal properties of I‐sR@MLNP, ICG‐II and other liposomes. Remarkably, as shown in Figures S6 and S7 (Supporting Information), the photothermal properties of I‐sR@MLNP are consistent with those of ICG‐II at the same concentration due to the inclusion of dispersed ICG‐II, and the photothermal conversion rate is as high as 95.4%.

Besides testing the photostability of liposomes, we also investigated temperature‐sensitive responsiveness of I‐sR@MLNP (Figure 2E). The gel electrophoresis experiments visually confirmed that I‐sR@MLNP could effectively release siRNA above 43 °C, while there was no siRNA band detectable at 37 °C (Figure 2F). Subsequently, we investigated the siRNA and ICG release efficiency of I‐sR@MLNP at different times, which we achieved visualization by using Cy5‐modified siRNA instead of the packaged siRNA in liposomes. As shown in Figure 2G and Figure S12 (Supporting Information), 85.7% of siRNA was released within 5 min at 46 °C, while ICG‐II showed 46.7% release. These findings indicated the temperature responsiveness of I‐sR@MLNP. Mechanistically, near‐infrared irradiation triggers localized heating that induces the DPPC phase transition, causing hydrocarbon chains to undergo trans‐to‐gauche conformational shifts. This structural rearrangement expands intermolecular spacing within the bilayer and generates transient membrane pores, enabling rapid efflux of encapsulated siRNA and ICG‐II via concentration gradients—consistent with established thermosensitive liposome behavior.^[^ ^18^ ^]^ Next, the stability of the constructed liposomes was investigated by monitoring the particle size of the nanoparticles after 14 days (Figure S5, Supporting Information). The engineered liposomes maintained hydrodynamic diameter consistency without significant particle aggregation after 14 days of placement. As shown in Figure S13A (Supporting Information), I‐sR@MLNP demonstrated viability for over 7 days with minimal leakage, even after 12 days. Additionally, we investigated the stability of I‐sR@MLNP in DMEM medium (containing 10% FBS) using gel electrophoresis. The results demonstrated the sustained presence of I‐sR@MLNP in DMEM complete medium for 36 h (Figure S13B, Supporting Information). Collectively, these results demonstrated that I‐sR@MLNP possessed photothermal properties, temperature‐sensitive responsiveness, and long‐term storage stability.

### 2.3 In Vitro and In Vivo Cellular Uptake and Lysosomal Escape Ability

**Figure 3:**
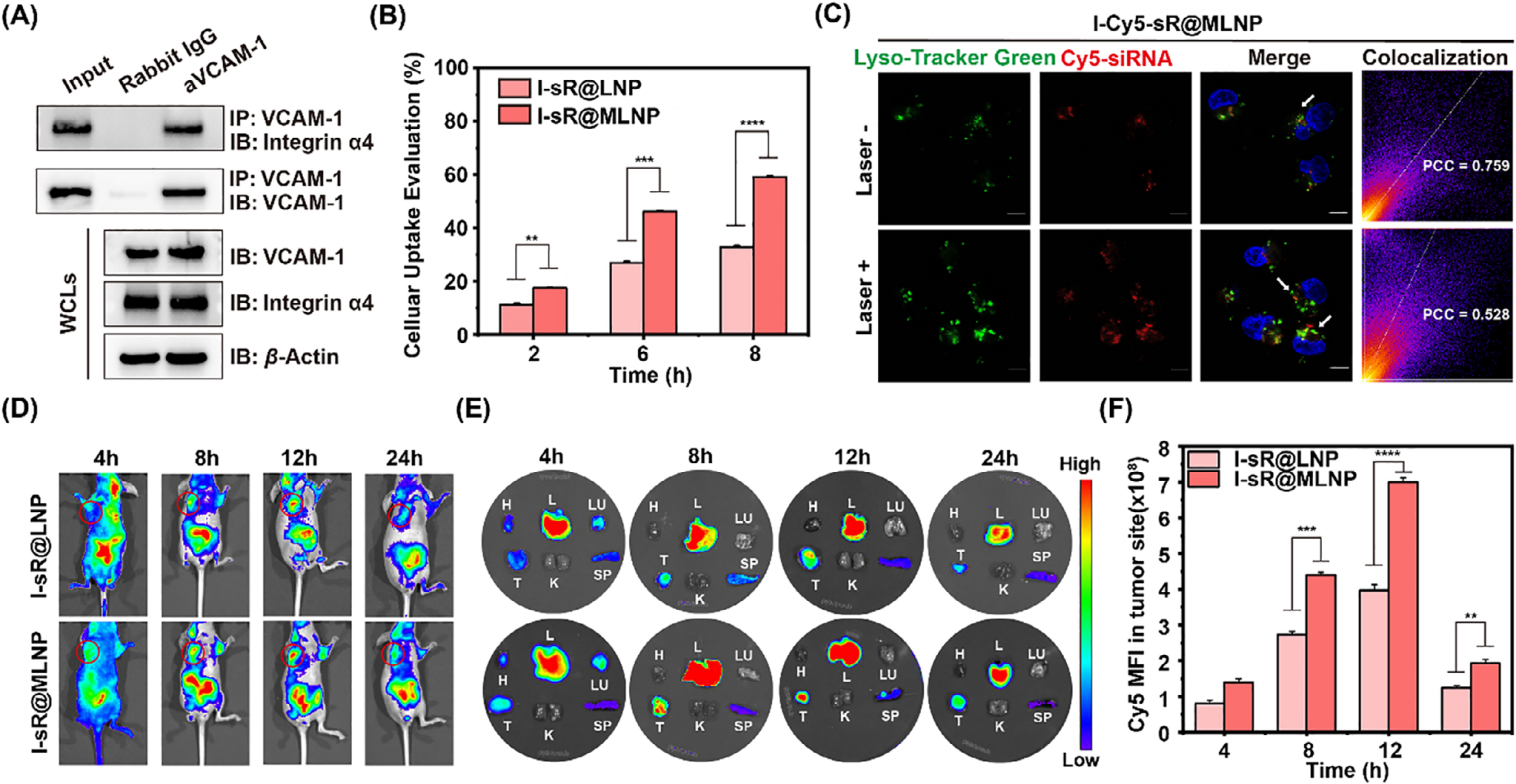
In vitro and in vivo cellular uptake and lysosomal escape ability. A) The CO‐IP analysis of Integrin α4 and VCAM‐1. B) The cell uptake evaluation in MDA‐MB‐231 cells with LNP and MLNP at different times (*n = 3*). C) The CLSM merge images of MDA‐MB‐231 cells with different approaches; Blue represents nuclear staining with Hoechst. Green represents is lysosomal staining with a lysosomal green commercial probe. Red represents cells treated with cy5. The co‐localization here is that the lysosome green probe is co‐localized with cy5. Scale bar: 20 µm. D) The systemic fluorescent imaging of mice at different time points after administration of various samples. E) The systemic fluorescent imaging of major organ at different time points after administration of various samples. H: Heart; L: Liver; LU: Lung; T: Tumor; K: Kidney; SP: Spleen. F) The average fluorescence intensity of tumor sites (*n = 3*). *p < 0.05, **p < 0.01, ***p < 0.001, ****p < 0.0001. Note: The laser is 785 nm and the density of the laser is 0.6 W cm^−2^.

To evaluate the biosafety of liposomes under non‐irradiated conditions, we performed MTT assays on four formulations (I@LNP, I@MLNP, I‐sR@LNP, and I‐sR@MLNP). The results demonstrated favorable biocompatibility of these liposomal systems (Figure S14, Supporting Information). Given potential immunogenicity concerns from macrophage‐derived membranes in I‐sR@MLNP, serum levels of pro‐inflammatory cytokines (TNF‐α, IFN‐γ, IL‐6, and IL‐1β) were quantified by ELISA. No significant cytokine elevation was observed in membrane‐coated groups (I‐sR@MLNP) compared to controls (I‐sR@LNP) (Figure S15, Supporting Information), indicating negligible immune activation. Subsequently, we systematically investigated the targeting specificity of the developed formulations. First, WB analysis revealed consistent expression of integrin α4 in both I‐sR@MLNP and M1‐type macrophages, along with VCAM‐1 overexpression in MDA‐MB‐231 cells (Figure S16, Supporting Information). Co‐immunoprecipitation (CO‐IP) assays further confirmed specific binding between integrin α4 on I‐sR@MLNP and VCAM‐1 protein (**Figure** **3**A), providing molecular‐level evidence for the active targeting mechanism. To quantitatively assess targeting efficiency, flow cytometric analysis showed time‐dependent cellular uptake of I‐sR@MLNP by MDA‐MB‐231 cells, reaching maximum internalization (59.6%) at 8 h post‐incubation (Figure 3B; Figure S17, Supporting Information). Notably, I‐sR@MLNP exhibited 1.8‐fold higher uptake efficiency compared to I‐sR@LNP.

While nanomaterials can effectively reach target cells, they are susceptible to lysosomal capture after entering the cell via endocytosis.^[^ ^18^, ^19^ ^]^ As evidenced by Figure 3C and Figures S18–S20 (Supporting Information), prior to light treatment, I‐sR@MLNP was predominantly localized within lysosomes, co‐localizing with lysosomal compartments stained with Lyso‐Tracker green. Following laser irradiation, the spatial separation of red fluorescence from green signals indicated successful lysosomal escape of I‐sR@MLNP mediated by photothermal effects. This phenomenon was primarily attributed to photothermally induced lysosomal membrane disruption. Importantly, the thermally triggered release of siRNA from the temperature‐sensitive response of I‐sR@MLNP under photothermal conditions ensured efficient cytoplasmic delivery for subsequent protein downregulation.^[^ ^18^, ^19^ ^]^ Building upon these in vitro findings, we further evaluated tumor‐targeting efficacy in an MDA‐MB‐231 xenograft mouse model. The uptake of Cy5‐labeled liposomes was investigated in a mouse model of xenografted MDA‐MB‐231 tumor. Real‐time fluorescence imaging demonstrated that Cy5‐labeled I‐sR@MLNP exhibited significantly enhanced tumor accumulation compared to conventional I‐sR@LNP formulations (Figure 3D–F; Figure S21, Supporting Information). In addition, quantitative analysis revealed peak tumor accumulation of I‐sR@MLNP at 12 h post‐injection, with a 1.76‐fold higher fluorescence intensity than the I‐sR@LNP control group. To evaluate the in vivo systemic pharmacokinetics of nanoparticles, Cy5.5‐labeled I‐sR@LNP and I‐sR@MLNP were intravenously administered to mice. Quantitative analysis of blood circulation kinetics (Figure S22, Supporting Information) revealed that macrophage membrane‐coated I‐sR@MLNP exhibited significantly prolonged half‐life compared to uncoated I‐sR@LNP, validating the role of macrophage membrane in reducing clearance by the mononuclear phagocyte system.^[^ ^20^ ^]^ Collectively, these findings demonstrate that the developed nanoparticles exhibited favorable targeting capability and lysosomal escape capacity.

### 2.4 The Efficacy of a Single‐Phase Photothermal Therapy In Vitro

**Figure 4:**
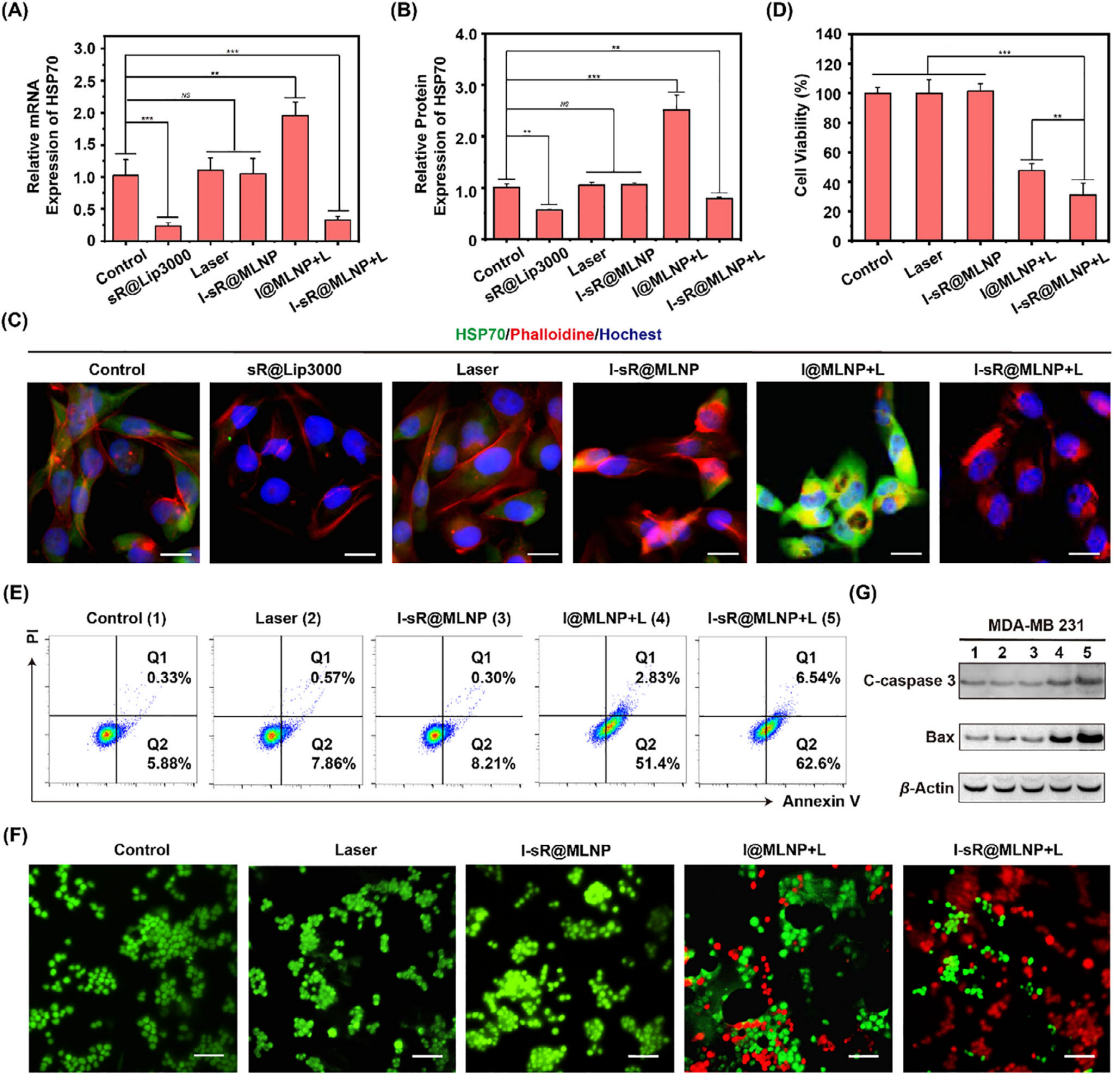
The efficacy of a single‐phase photothermal therapy in vitro. A) The relative mRNA expression levels HSP70 in MDA‐MB‐231 cells after incubation with different approaches (*n = 3*). B) The relative protein expression levels HSP70 in MDA‐MB‐231 cells after incubation with different approaches (*n = 3*). C) The CLSM merge images with different treatments. Scale bar: 20 µm. D) The cell viability in MDA‐MB‐231 cells after incubation with different approaches (*n = 3*). E) The flow cytometric analysis of cell apoptosis with different treatments using the Annexin V‐FITC/PI staining. F) The live and dead cell staining in MDA‐MB‐231 cells with different approaches. All cells were stained with calcein‐AM and PI. Scale bar: 100 µm. G) The WB analysis of C‐caspase 3 and bax after incubation with different approaches. *p < 0.05, **p < 0.01, ***p < 0.001, ****p < 0.0001. Note: The laser is 785 nm and the density of the laser is 0.6 W cm^−2^. I@MLNP and I‐sR@MLNP contain 50 µg mL^−1^ ICG‐II. And I‐sR@MLNP contain 100 nm siRNA. *p <0.05, **p < 0.01, ***p < 0.001, ****p < 0.0001.

After confirming that siRNA was effectively released, verified its ability to silence the expression of HSP70. In this study, Lipofectamine 3000 (Lipo3000), a well‐established transfection reagent for siRNA delivery, served as the positive control. We established six experimental groups: negative control, laser, sR@Lipo3000 (positive control), I‐sR@MLNP, I@MLNP with laser, and I‐sR@MLNP with laser. Both qPCR and WB results showed that laser irradiation induced significant upregulation of HSP70 mRNA/protein in PBS and I@MLNP groups compared to baseline levels (**Figure** **4**A,B). Conversely, the sR@Lipo3000 control and I‐sR@MLNP under laser group demonstrated marked suppression of HSP70 expression at both transcriptional and translational levels. Notably, the HSP70 expression in irradiated I@MLNP group exceeded that of the positive control, likely attributable to laser‐induced HSP70 overexpression overriding baseline expression. Additionally, the immunofluorescence imaging corroborated these findings (Figure 4C; Figure S23, Supporting Information). A distinct attenuation of green fluorescence was observed in irradiated I‐sR@MLNP groups, confirming effective siRNA‐mediated HSP70 knockdown. Importantly, non‐irradiated I‐sR@MLNP showed no silencing activity across all detection methods (qPCR, WB, imaging), demonstrating the light‐activated gene regulation capability of this delivery system.^[^ ^21^ ^]^ Considering that the presence of RNase might affect siRNA knockdown efficiency, I‐sR@MLNP was subsequently incubated with RNase A (0.1 mg mL^−1^, 37 °C) for 1 h. Considering potential RNase effects on siRNA knockdown efficiency, I‐sR@MLNP was incubated with RNase A (0.1 mg mL^−1^, 37 °C) for 1 h. Subsequent agarose gel electrophoresis revealed intact nanocarrier‐encapsulated siRNA in I‐sR@MLNP, while free siRNA underwent complete degradation (Figure S24A, Supporting Information), confirming effective siRNA shielding by the liposome coating. Bioactivity retention post‐RNase exposure was verified by delivering pretreated formulations to MDA‐MB‐231 cells: I‐sR@MLNP maintained potent gene silencing, reducing HSP70 mRNA and protein levels (Figure S24B,C, Supporting Information). In contrast, RNase‐treated sR@Lipo3000 control showed no significant knockdown due to siRNA degradation.

To systematically evaluate the photothermal therapeutic efficacy, we conducted in vitro MPTT assessment under standardized laser parameters (785 nm, 0.6 W cm^−^^2^, 5 min irradiation). Experimental groups included: control, laser, I‐sR@MLNP, I@MLNP with laser, and I‐sR@MLNP with laser. Both I‐sR@LNP and I‐sR@MLNP under laser demonstrated significantly enhanced cytotoxicity compared to PBS and laser‐only, achieving 51% and 72% cell death rates, respectively (Figure 4D). This differential efficacy correlated with nanoparticle internalization efficiency, as confirmed by cellular uptake studies. However, the MPTT effect of I@MLNP was not great due to the high expression of HSP70 protein at 46 °C, preventing thermal damage to the cells, resulting in a cell death rate of about 48% (Figure S25, Supporting Information). In contrast, I‐sR@MLNP‐mediated HSP70 silencing through light‐activated siRNA release effectively disrupted cellular heat‐shock response, increasing mortality by 24% compared to I@MLNP under laser. Flow cytometric quantification of apoptosis ratios substantiated this finding (Figure 4E). Live and dead cell staining visually confirmed that I‐sR@MLNP under laser exhibited specific photothermal therapeutic effects (Figure 4F; Figure S26, Supporting Information). Mechanistically, WB analysis revealed significant upregulation of cleaved caspase‐3 (C‐caspase 3) (1.6‐fold vs control) in the I‐sR@MLNP + laser group (Figure 4G; Figure S27, Supporting Information), indicating apoptosis as the predominant cell death pathway.

### 2.5 Exploration of siRNA Regulation Kinetics In Vitro

**Figure 5:**
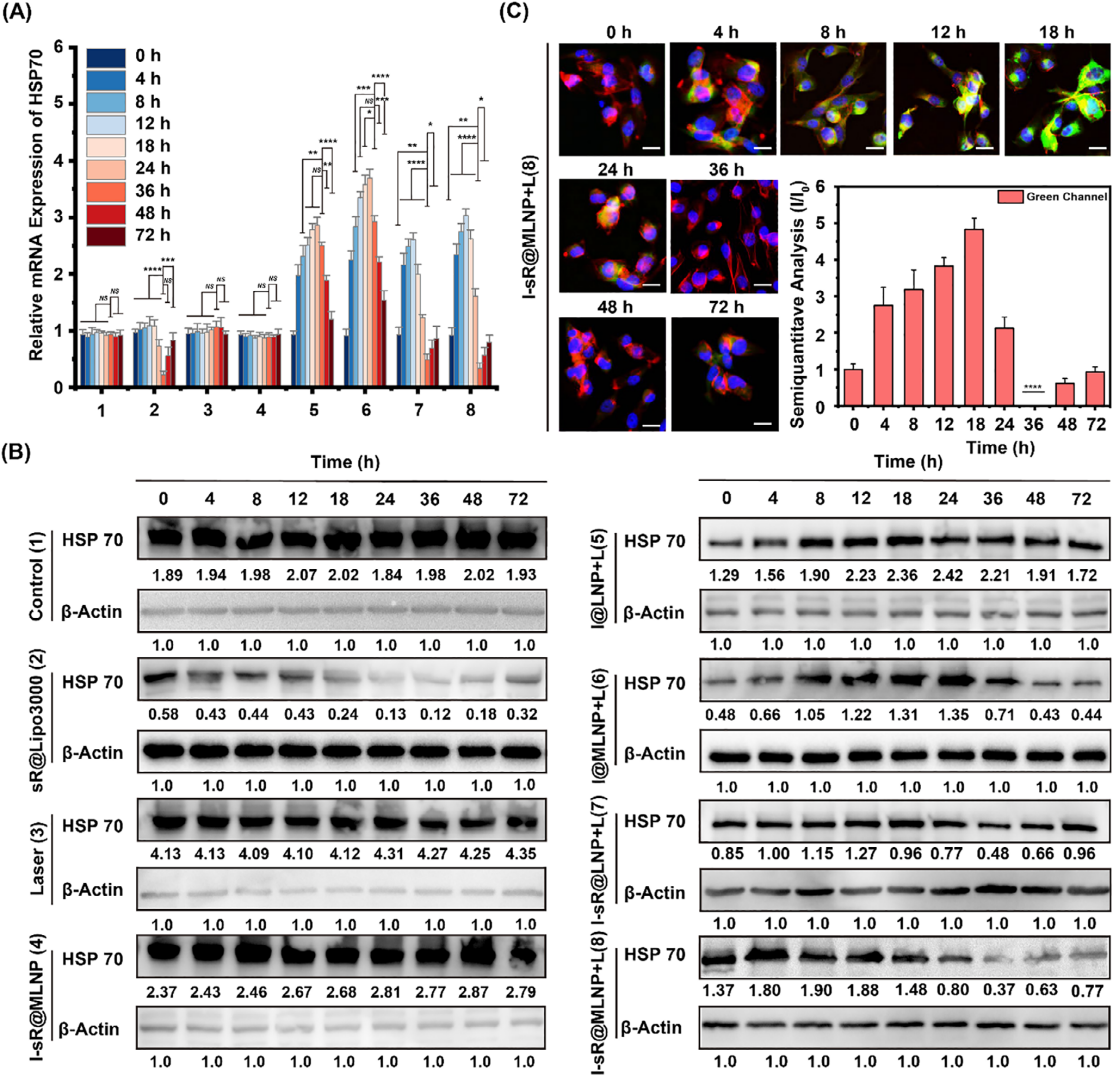
A) The relative HSP70 mRNA levels in MDA‐MB‐231 cells after incubation with different treatments. Note: 1 to 8 respectively represent PBS, sR@Lipo3000, Laser, I‐sR@MLNP, I@LNP with Laser, I@MLNP with Laser, I‐sR@LNP with Laser, and I‐sR@MLNP with Laser (*n = 3*). B) The WB analysis on the HSP70 level with different treatments. Scale bar: 20 µm. C) The CLSM merge images treated with I‐sR@MLNP with Laser at different times (*n = 3*). Note: I@LNP, I@MLNP, I‐sR@LNP and I‐sR@MLNP contain 50 µg mL^−1^ ICG‐II. sR@Lipo3000, I‐sR@LNP and I‐sR@MLNP contain 100 nm siRNA at the same time. L is used to stand for laser. Scale bar: 50 µm. *p < 0.05, **p < 0.01, ***p < 0.001, ****p < 0.0001.

To determine the temporal dynamics of HSP70 siRNA regulation, we conducted a 72 h kinetic study comparing multiple formulations. As delineated in **Figure** **5**A, temporal analysis of HSP70 mRNA levels revealed critical kinetic differences among experimental groups. Both I@LNP and I@MLNP exhibited acute upregulation of HSP70 mRNA within 24 h post‐laser irradiation, followed by gradual return to baseline levels by 72 h. Although I‐sR@LNP and I‐sR@MLNP also showed initial HSP70 induction under laser exposure, their mRNA elevation was attenuated by 30–35% compared to non‐siRNA counterparts, demonstrating early‐stage gene silencing activity. Notably, siRNA‐mediated suppression became dominant from 18 h onward, with mRNA levels progressively declining to reach minimal expression at 36 h. Additionally, free siRNA failed to reduce HSP70 expression at any time point, due to its well‐established poor cellular internalization (Figure S28A, Supporting Information).^[^ ^22^ ^]^ These results proved that the optimal gene silencing effect of siRNA at 36 h. WB analysis further corroborated this temporal pattern, showing minimal HSP70 protein expression at 36 h post‐treatment in siRNA‐loaded formulations (Figure 5B; Figure S28B, Supporting Information). Simultaneously, CLSM fluorescence images also validated that the green fluorescence intensity of I‐sR@LNP and I‐sR@MLNP groups decreased significantly after 36 h of laser irradiation (Figure 5C; Figures S29–S36, Supporting Information). Notably, the positive control (sR@Lipo3000) exhibited analogous kinetics but with earlier onset. This comprehensive timeline established 36 h as the therapeutic window for optimal HSP70 suppression, providing critical guidance for subsequent in vivo treatment scheduling.

**Figure 6:**
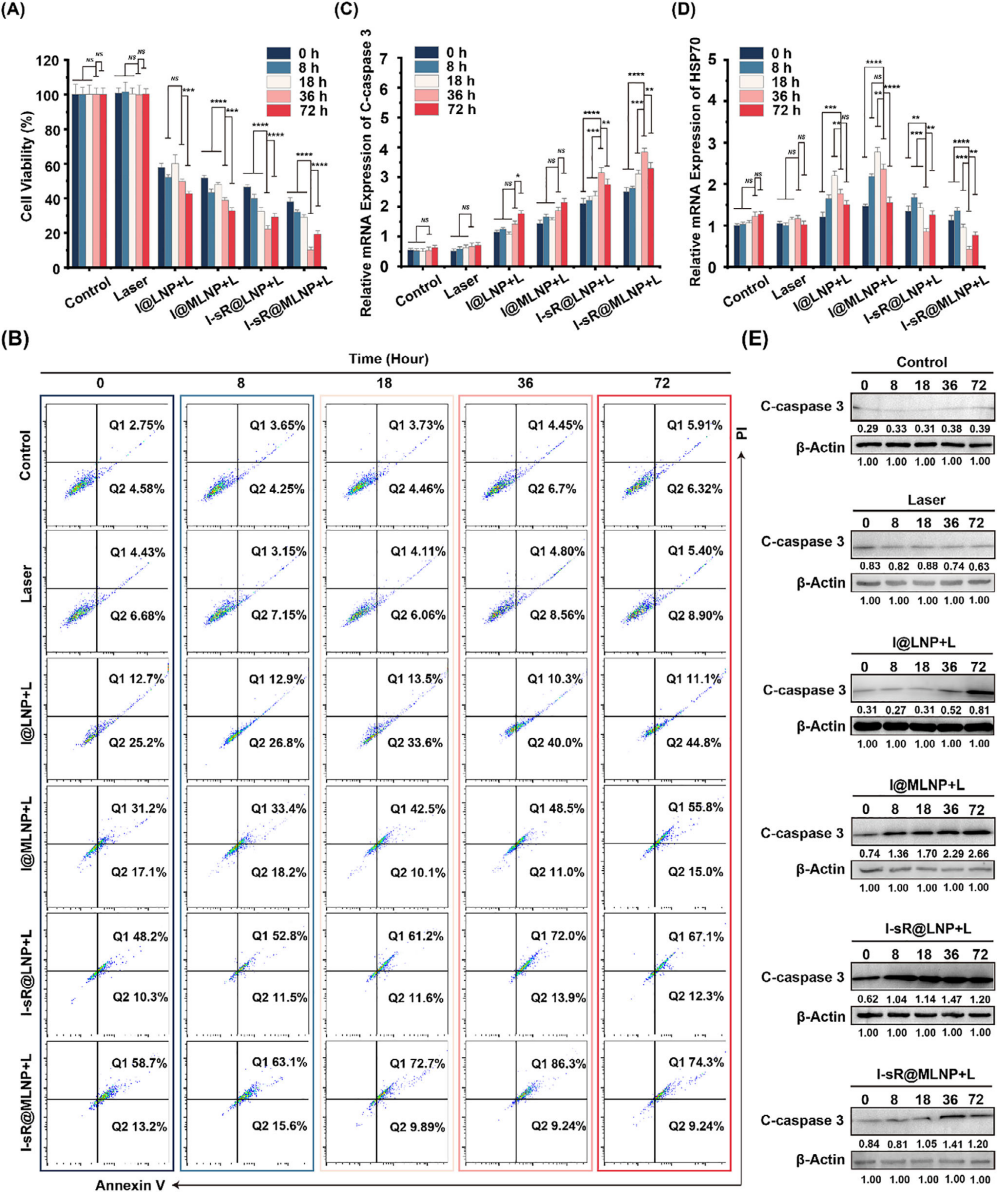
A) Cell viabilities after double treatment in each group at different times (*n = 3*). B) The flow cytometric analysis of cell apoptosis with different treatments using the Annexin V‐FITC/PI staining. C) The relative C‐caspase 3 mRNA levels mRNA levels in MDA‐MB‐231 cells after incubation with different treatments (*n = 3*). D) The relative HSP70 mRNA levels in MDA‐MB‐231 cells after incubation with different treatments (*n = 3*). E) The WB analysis on the C‐caspase 3 levels with different treatments. Note: The laser is 785 nm and the density of the laser is 0.6 W cm^−2^. *p < 0.05, **p < 0.01, ***p < 0.001, ****p < 0.0001.

To establish temporal coordination between siRNA activity and photothermal efficacy, we selected five time points (0, 8, 18, 36, and 72 h) as time intervals to perform dual‐phase PTT. As shown in **Figure** **6**A, both I‐sR@LNP and I‐sR@MLNP achieved maximal cytotoxicity (73.4% and 90.5% cell death respectively) at the 36 h interval. Flow cytometry quantification through annexin V/PI co‐staining further confirmed this temporal optimization, showing apoptotic rates of 85.9% and 95.4% for I‐sR@LNP and I‐sR@MLNP respectively in the 36 h regimen higher than 18 or 72 hour interval controls (Figure 6B). These results suggested that a 36 h interval of dual MPTT therapy displayed a higher mortality rate for cells treated with liposomes containing siRNA compared to other treatment strategies. Additionally, we analyzed the mRNA and protein expression of HSP70 and C‐caspase 3 in the cells treated with different treatment strategies. The qPCR results confirmed that I‐sR@MLNP group had the highest expression of C‐caspase 3 under the dual MPTT treatment strategy with an interval of 36 h (Figure 6C). This coordinated effect was maintained through sustained HSP70 suppression (Figure 6D). We obtained similar, consistent experimental results on protein expression levels by WB experiment (Figure 6E). The above experimental results all confirmed the superiority of the dual‐MPTT strategy based on the optimal action time of siRNA. This chronotherapeutic approach establishes a paradigm for precision nanomedicine by rationally integrating gene regulation kinetics with photothermal dose delivery.

**Figure 7:**
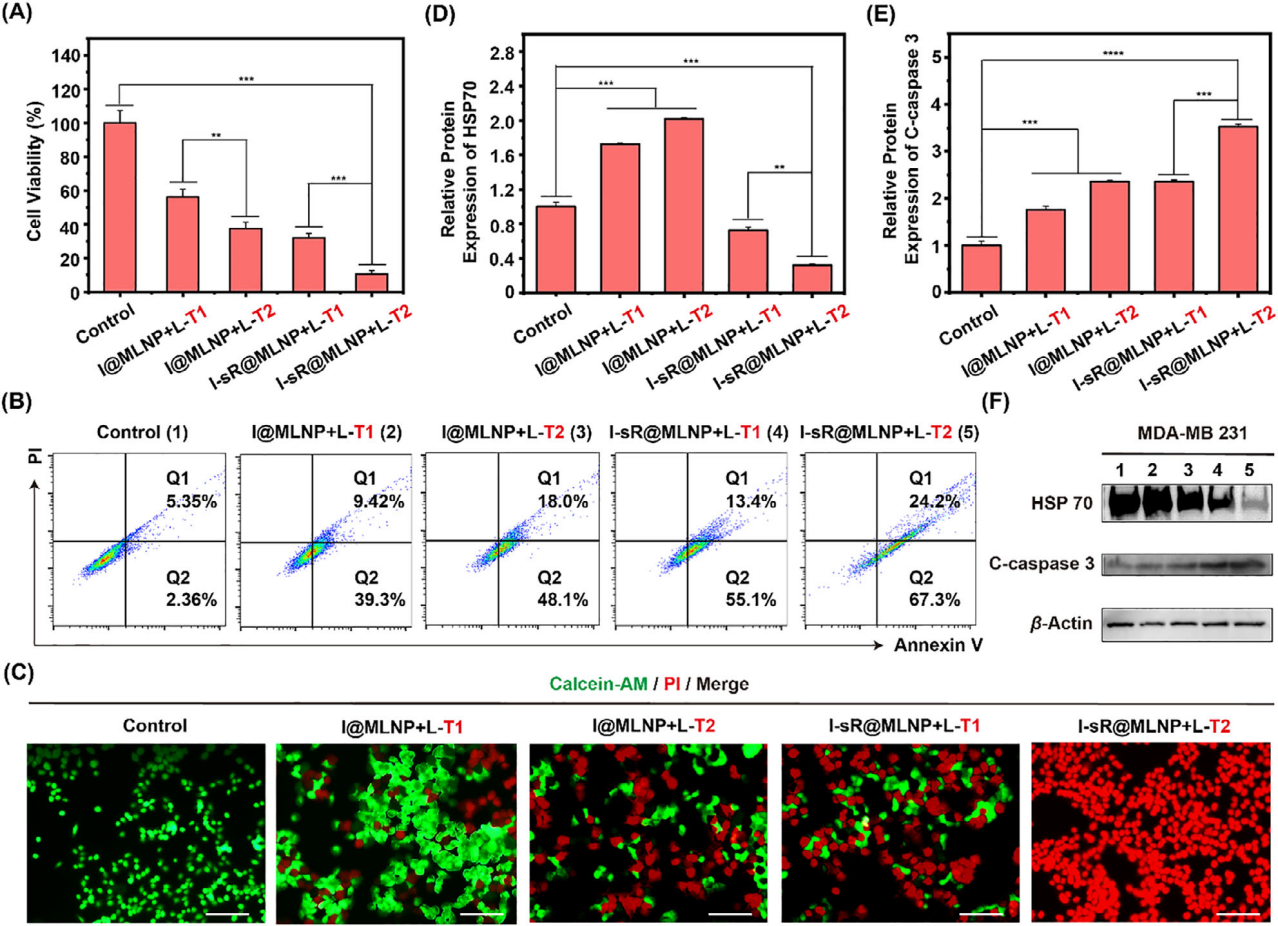
A) The cell viability in MDA‐MB‐231 cells after incubation with different approaches (*n = 3*). B) The flow cytometric analysis of cell apoptosis with different treatments using the Annexin V‐FITC/PI staining. C) The live and dead cell staining in MDA‐MB‐231 cells with different approaches. All cells were stained with calcein‐AM and PI. Scale bar: 100 µm. D) The relative expression levels of HSP70 with different treatments (*n = 3*). E) The expression levels of Caspase 3 with different treatments (*n = 3*). F) The WB analysis of C‐caspase 3 and HSP70 after incubation with different approaches. *p < 0.05, **p < 0.01, ***p < 0.001, ****p < 0.0001.

We further conducted a comparative evaluation of single versus dual photothermal treatment regimens, where numerical indices denote treatment frequency. Quantitative analysis demonstrated substantial therapeutic enhancement in the dual‐irradiation protocol: I‐sR@MLNP with two laser exposures (36 h interval) achieved 92.5% cell death, compared to 64.5% cytotoxicity in the single‐dose group (**Figure** **7**A,B). This efficacy escalation was visually confirmed by live/dead staining showing 44.7% reduction in viable cell density (Figure 7C; Figure S37, Supporting Information). The expression of HSP70 and C‐caspase 3 in different treatments were further investigated (Figure 7D–F). Relative to the PBS group, HSP70 protein levels in I@MLNP groups exhibited progressive upregulation, reaching 1.7‐fold with single treatment and 2.2‐fold under dual irradiation (Figure 7D,F). Conversely, dual‐irradiated I‐sR@MLNP demonstrated 59.6% suppression of HSP70 induction compared with that of single‐dose, concomitant with 1.6‐fold elevation of C‐caspase‐3 compared to single‐PTT controls, which attributed to the maximum influence of siRNA released after the initial light exposure.

### 2.6 Evaluation of In Vivo Uptake and Dual‐Phase Photothermal Therapy

**Figure 8:**
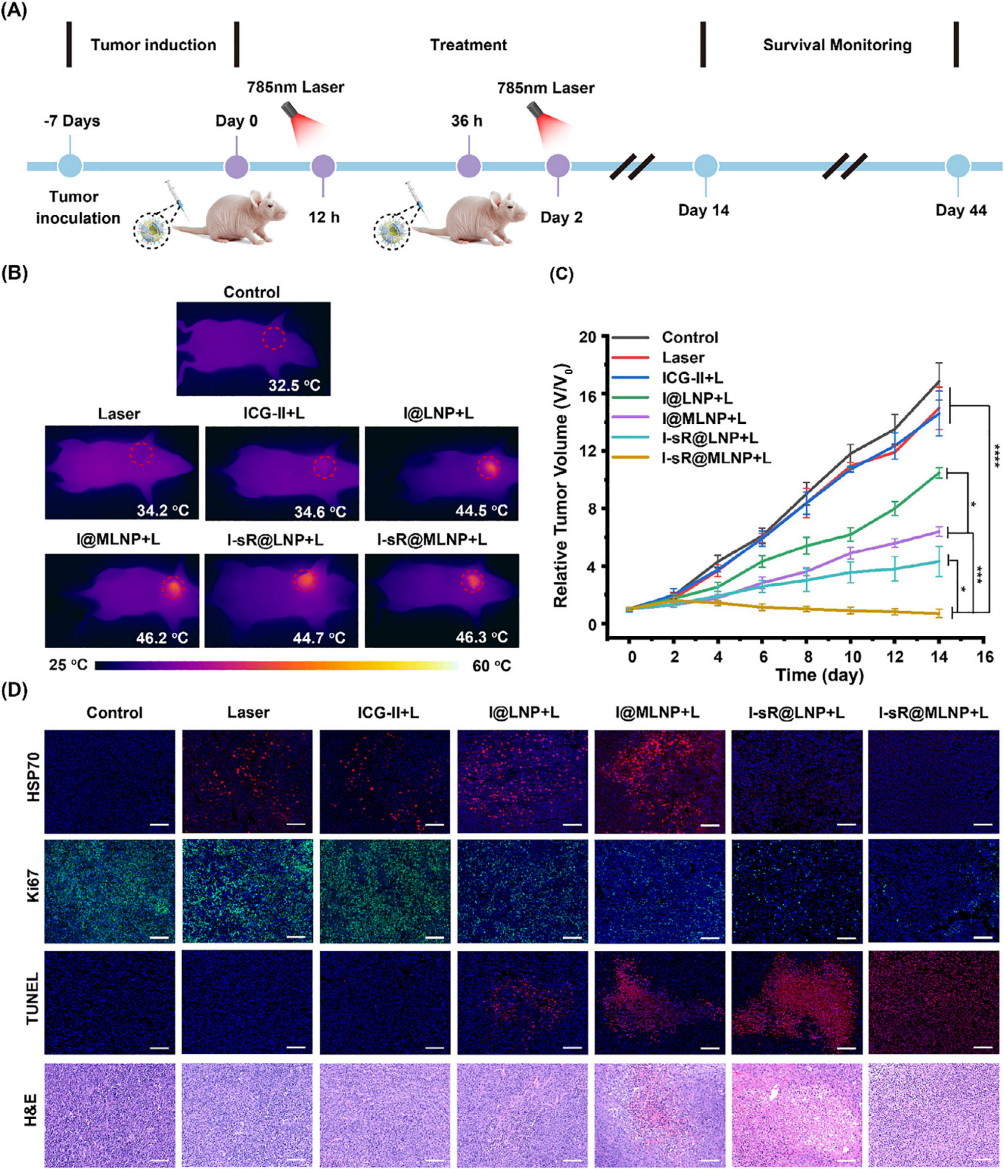
Evaluation of in vivo uptake and dual‐phase photothermal therapy. A) The evaluation protocol of MPTT for MDA‐MB‐231 tumor‐bearing animal models under laser with different treatments. B) The IR imaging photograph with different treatments after 5 min. C)The growth profiles of different groups of tumors (*n = 5*). D) The H&E and immunofluorescence staining for different groups of tumors. Scale bar: 100 µm. *p < 0.05, **p < 0.01, ***p < 0.001, ****p < 0.0001. Note: The laser is 785 nm and the density of the laser is 0.6 W cm^−2^.

Based on the in vivo liposome uptake studies, we established a 12 h interval between administration and therapeutic laser intervention. With the exception of the control group mice, experimental cohorts received one of the following treatments via intravenous injection: ICG‐II (50 µg mL^−1^, 200 µL), I@LNP (containing 50 µg mL^−1^ ICG‐II, 200 µL), I@MLNP (containing 50 µg mL^−1^ ICG‐II, 200 µL), I‐sR@LNP (containing 50 µg mL^−1^ ICG‐II and 100 nm siRNA, 200 µL) and I‐sR@MLNP (containing 50 µg mL^−1^ ICG‐II and 100 nm siRNA, 200 µL). All groups subsequently underwent laser irradiation, with infrared thermography confirming maintenance of intertumoral temperatures at 46.0 during treatment (**Figure** **8**B; Figure S38, Supporting Information). Building on comprehensive in vitro optimization of siRNA temporal activity, we developed a photothermal treatment protocol (Figure 8A) featuring sequential interventions. This approach entailed a 5‐min laser irradiation performed 12 h post‐injection, primarily directed at facilitating temperature‐responsive release of both ICG‐II and siRNA. A second therapeutic administration followed 24 h later. This dual‐injection strategy was designed to address two key pharmacokinetic considerations: 1) complete metabolic clearance of initially released ICG‐II within 36 h post‐PTT, and 2) temporal alignment of peak drug uptake from the second injection (12 h post‐administration) with optimal siRNA activity from the first treatment cycle. A subsequent round of photothermal treatment was then executed under identical temperature parameters. Post‐treatment, each group was monitored for 14 days, using the initial treatment as the reference point. As showed in Figure 8C and Figure S39 (Supporting Information), the mice treated with ICG‐II under a laser exhibited no significant changes in tumors relative to the PBS and laser groups. This equivalence likely stems from ICG‐II's inherent lack of tumor‐targeting specificity. While I@LNP and I‐sR@LNP formulations achieved passive tumor accumulation through the enhanced permeability and retention (EPR) effect, their therapeutic performance remained substantially inferior to actively targeted counterparts (I@MLNP and I‐sR@MLNP). Notably, the I‐sR@MLNP group exhibited superior MPTT outcomes comparted with I@MLNP, revealing siRNA co‐delivery contributing significantly to tumor suppression. Representative photographic documentation of tumor‐bearing mice and dissected tumor tissue provided visual confirmation of therapeutic efficacy (Figures S40 and S41, Supporting Information). Throughout the treatment and monitoring cycle, there was no discernible change in the body weight of the mice. The results underscored the negligible impact of the constructed drug on the mouse organism (Figure S42A, Supporting Information). Survival analysis conducted over 30 days post‐treatment demonstrated significantly prolonged survival in mice receiving targeted I‐sR@MLNP therapy with laser irradiation compared to other experimental groups (Figure S42B, Supporting Information).

To evaluate siRNA‐mediated HSP70 protein downregulation in tumor tissue, we performed comprehensive immunofluorescence analysis (Figure 8D). Quantitative assessment revealed significant HSP70 upregulation in the laser group and among mice injected with ICG‐II, I@LNP, and I@MLNP after laser, relative to the control group. In stark contrast, siRNA‐loaded formulations (I‐sR@LNP and I‐sR@MLNP) exhibited marked HSP70 suppression under identical photothermal conditions, consistent with prior in vitro gene silencing observations. Additionally, the I‐sR@MLNP group showed a 72% reduction in Ki67‐positive cells versus controls, confirming effective suppression of tumor proliferation. Concurrently, hematoxylin and eosin (HE) staining and terminal deoxynucleotidyl transferase dUTP nick end labeling (TUNEL) staining were also performed on tumor tissue samples collected after 24 h of dual‐phase treatment (Figure 8D). The results from HE staining and TUNEL staining further affirmed the outstanding MPTT efficacy of I‐sR@MLNP under the new therapeutic strategy. The in vivo safety of the injected drugs was evaluated using a final administered dose of 10 mg kg^−1^ ICG‐II and 1.2 mg kg^−1^ siRNA. Blood chemistry profiles analyzed 48‐h and 20‐day post‐injection showed no abnormal hepatic or renal biomarkers (Figures S43 and S44, Supporting Information). Histopathological assessment of major organs via H&E staining revealed no structural abnormalities, confirming the formulations' biocompatibility. These findings collectively establish I‐sR@MLNP as both a potent and biosafe nanoplatform for MPTT.

## 3 Conclusion

In this study, we established a precisely‐timed approach to overcome HSP70‐mediated thermo‐resistance in MPTT cancer therapy through temporally optimized siRNA delivery. Our three component temperature‐responsive hybrid nanocarrier (I‐sR@MLNP) addressed the poor efficacy of MPTT through three integrated mechanisms: 1) extremely high PTCE (95.4%) from ICG‐II encapsulation, enabling efficient photothermal conversion; 2) active targeting of VCAM‐1M1 by integrin α4 abundantly expressed on the surface of the macrophage membrane coating, yielding 4.7‐fold greater tumor accumulation than non‐targeted liposomes; 3) controlled delivery of HSP70 siRNA to silence HSP70 expression, allowing temporal control of tumor cells' critical heat stress defense for more effective treatment. Upon irradiation by a 785 nm near‐infrared laser, I‐sR@MLNP facilitated controlled co‐release of therapeutic agents through lysosomal escape, enabling precise spatiotemporal control over therapeutic delivery. In vivo experiments confirmed I‐sR@MLNP to significantly suppress tumor growth while presenting a favorable safety profile both pre‐ and post‐treatment based on cellular viability studies and organ tissue and blood biochemistry analysis 2 and 20 days post‐treatment.

Most significantly, our systematic kinetic profiling of HSP70 expression revealed a critical 36 h window during which siRNA‐mediated gene silencing maximally sensitized cancer cells to subsequent thermal stress. This timing‐optimized, dual‐phase MPTT approach demonstrated 1.87‐ and 1.43‐ fold enhanced therapeutic efficacy compared to conventional single‐treatment or non‐optimized dual‐treatment protocols. By aligning treatment timing with the underlying molecular kinetics of HSP70 suppression, we provide a novel strategy to maximize therapeutic outcomes while maintaining the reduced side‐effect profile of mild‐temperature interventions.

Our findings point toward potential translational applications in addressing the ongoing challenges of triple‐negative breast cancer therapy. Recent literature has highlighted the potential of biomimetic delivery systems, and we provide compelling additional evidence for the exceptional targeting affinity of macrophage membrane‐derived nanoparticles toward TNBC,^[^ ^23^ ^]^ supporting this platform as a reliable delivery vector for this historically difficult‐to‐target malignancy. While previous investigations have explored alternative photothermal approaches such as photoimmunotherapy and antibody‐conjugated PTT for TNBC,^[^ ^24^ ^]^ our work represents the first demonstration of significant TNBC tumor volume reduction using the milder MPTT approach alone. Aside direct treatment of the primary tumor, other preclinical studies have suggested potential benefits of adjuvant PTT administered to the surgical wound bed in preventing local recurrence.^[^ ^25^ ^]^ Despite these various investigational directions, TNBC remains a formidable clinical challenge with inadequate treatment options. Encouraging Phase I/II clinical results (NCT03202446) published in 2011^[^ ^26^ ^]^ demonstrated potential for PTT in treating refractory breast cancers, yet broader clinical adoption has not materialized—with the subsequent Phase 3 trial concluded in 2018 without published follow‐up results. Our timing‐optimized siRNA‐MPTT strategy offers a fundamentally new direction that may help overcome these persistent therapeutic barriers in TNBC, potentially transforming these promising but currently insufficient approaches into clinically meaningful interventions.

In summary, this study introduces a temporal dimension to siRNA‐enhanced phototherapy that addresses a fundamental challenge in nanomedicine: optimizing treatment scheduling to align with underlying molecular kinetics. While our current findings focus on HSP70 dynamics in TNBC, the concept of chrono‐modulated treatment regimens has broader implications. Future investigations should systematically profile temporal dynamics of other molecular targets (e.g., HSP90) to establish universal principles for timing‐optimized siRNA‐based combination therapies—work that forms the foundation of our ongoing research program. By incorporating molecular kinetics into treatment scheduling, this approach creates a framework for maximizing therapeutic efficacy while minimizing required dosing, representing a significant advancement in precision nanomedicine for cancer therapy.

## 4 Experimental Section

### Materials and Instruments

ICG‐II was synthesized in the laboratory.^[^ ^7^ ^]^ The reagents were sourced from various companies. 1,2‐Dipalmitoyl‐sn‐glycero‐3‐phosphorylcholine (DPPC) was acquired from Peng Sheng Biotechnology Co. (Shanghai, China), while DSPE‐PEG‐2000 and cholesterol were obtained from Bide Pharmatech. Ltd. (Shanghai, China). The HSP 70 siRNA (Sequence: 5′‐CCAUUGAGGAGGAGGUAGAUUAdTdT‐3′; Nonsense sequence: 5′‐GUACCAAUCCUA CUGCCGAdTdT‐3′),^[^ ^2^ ^]^ and Cy5‐labeled siRNAs were sourced from OBiO Tech. Inc. (Shanghai, China). Other biological experimental materials were mainly purchased from MedChemExpress Inc. (Shanghai, China) and Beyotime Biotech. Inc. (Shanghai, China). Dynamic light scattering (DLS) and zeta potential analysis were conducted using a Malvern Zetasizer Nano ZS90 (Worcestershire, United Kingdom). The morphology and size of the nanoparticles were observed with FEI Tecnai F20 (New York, America). The UV–vis–NIR absorption spectra were recorded on a Shimadzu UV‐1900i spectrometer, and fluorescence spectra were captured on a Shimadzu RF‐6000 spectrometer (Kyoto, Japan). The 785 nm and 880 nm lasers were supplied by Changchun Laser Technology Co. Ltd (Changchun, China), The infrared (IR) thermal images were captured using a FLIR E6 thermal imaging camera. Cell imaging was documented with a laser confocal microscope (OLYMPUS FV3000, Tokyo, Japan). qPCR data were obtained using a ThermoFisher ABI7300 (Wilmington, America). The cell viability was assessed with a Molecular Devices SpectraMax i3X (Sunnyvale, America). Live/dead cell staining images were captured with a Zeiss Axio Vert A1 FL‐LED (Oberkochen, Germany). Flow analysis was performed using a BD FacsversaTM flow cytometer (Burlingame, America).

### Cells culture and Animals

RAW264.7 cells (RRID: CVCL_0493) and MDA‐MB‐231 cells (CVCL_0062) were procured from the China Center for Type Culture Collection (CCTCC). Female Balb/c Nude mice (weighing 18–20 g) were obtained from GemPharmatech. Inc. (Nanjing, China). The animal experiment protocol was approved by the Animal Ethics and Welfare Committee of Nanjing University (AEWC, IACUC‐2208004).

### Preparation and Characterization of I@LNP and I(J)@LNP

1) ICG‐II was synthesized based on previously reported methods within the laboratory.^[^ ^7^ ^]^ I@LNP and I(J)@LNP were prepared using thin film hydration. Specifically, DPPC, DSPE‐PEG‐2000, cholesterol, and ICG‐II (in a ratio of 10:3:3:2) were dissolved in anhydrous trichloromethane and transferred to a round‐bottom flask. The solution was evaporated in a rotary evaporator at 150 rpm to form thin films. The resulting mixture was stirred in DI water using a mechanical stirrer. I‐LNP was stirred for 1 h at 4 °C in an ice bath, while I(J)@LNP was stirred at 40 °C until no J aggregates were forming. The combined solution underwent overnight dialysis to remove unencapsulated small molecules and was then stored at 4 °C.
2) The particle size and potential of the nanoparticles were closely monitored. Transmission electron microscopy was employed to further scrutinize the morphological state and size of I@LNP in comparison to I(J)@LNP. Simultaneously, UV–vis absorption spectra of I@LNP and I(J)@LNP solutions were examined to confirm the encapsulation of ICG‐II within the nanoparticles. Subsequently, after a 14‐day incubation, the particle size of both nanoparticles was reevaluated to gauge the chemical stability of the formulations.
3) UV–vis absorption spectra were generated in DMSO, varying the concentrations of ICG‐II to establish standard curves. Following this, the UV–vis absorption of the prepared nanoparticles was systematically monitored and calculated using the provided equation. The drug loading of I@LNP and I(J)@LNP was quantified. Initially, the two purified nanoparticles underwent lyophilization.
(1) $$\mathrm{EE}=\frac{\mathrm{Intensit}y_{\left(\mathrm{NPs}\right)}}{\mathrm{Intensit}y_{\left(\mathrm{ICG}-\mathrm{II}\;\mathrm{in}\;\mathrm{DMSO}\right)}}\times 100\%$$

where EE is the encapsulation rate, Intensity (NPs) is the UV‐absorption intensity of the prepared nanoparticles, and Intensity (ICG‐II) is the UV‐absorption intensity of ICG‐II in DMSO corresponding to the standard curve at the time of nanoparticle formation.

4) The weight of the resulting lyophilized powder was considered the total formulation amount. Subsequently, the lyophilized powder was dissolved in DMSO and subjected to UV absorption analysis using a UV spectrophotometer. By referencing the standard UV–vis absorption spectra of ICG‐II, the encapsulated ICG‐II content was determined. The drug loading of the nanoparticles was calculated using the following equation:
(2) $$\mathrm{DL}=\frac{\mathrm{Qualit}y_{\left(\mathrm{ICG}-\mathrm{II}\right)}}{\mathrm{Total}\;\mathrm{Quality}}\times 100\%$$

### Photothermal Property Testing of I@LNP and I(J)@LNP

Utilizing the encapsulation rate and drug loading rate criteria, I@LNP and I(J)@LNP containing ICG‐II at a concentration of 80 µg mL^−1^ were formulated. Various nanoparticles were subjected to continuous laser exposure at 785 nm for 8 min. The photothermal performances of water, I@LNP, and I(J)@LNP were concurrently assessed under 1 W cm^−^^2^ laser illumination. Infrared thermograms of I@LNP and I(J)@LNP were acquired, followed by the calculation of the photothermal conversion of the two nanoparticles in accordance with a previously published methodology.^[^ ^1^ ^]^

### Preparation and Characterization of I@MLNP, I‐sR@LNP, and I‐sR@MLNP

1) To obtain purified M1 macrophage cell, RAW 264.7 cells were polarized to the M1 phenotype by incubating the cells with lipopolysaccharide (LPS, Sigma, #93572‐42‐0). In detail, 1×10^5^ RAW 264.7 cells dispersed in 500 µL of DMEM medium were inoculated in each well of a 24‐well plate and incubated at 37^ °^C. Then, LPS (500 ng mL^−1^) was added to the wells. After 24 h of treatment, macrophage types were assessed by flow cytometry stained with PE anti‐mouse CD86 (Biolegend, #105007), PerCP/Cyanine5.5 anti‐mouse CD11b (Biolegend, #101227), Alexa Flour488 anti‐mouse F4/80 (Biolegend, #123120), APC anti‐mouse CD206 (Biolegend, #141707). Flow cytometry results were analyzed by FlowJo software.
2) To obtain purified M1 macrophage cell membranes, M1 macrophage cells were scraped using a cell scraper, followed by centrifugation at 1000 rpm for 3 min at 4 °C for isolation. The supernatant was discarded, and the pellet was resuspended in PBS.^[^ ^27^ ^]^ The suspension was sonicated with an ultrasonic probe for 3 min (25% power, on 2s, off 3s). After centrifugation at 1000 g for 5 min, the supernatant was collected to eliminate residual cells. Further purification was achieved by centrifugation at 3000 g for 5 min to remove debris. Finally, the resulting precipitate was isolated for subsequent use.
3) Films comprising DPPC, DSPE‐PEG‐2000, cholesterol, and ICG‐II (in a ratio of 10:3:3:2) were formulated as outlined earlier. The macrophage membrane, previously prepared, was dissolved in DEPC water. This DEPC water solution, containing the macrophage membranes, was then combined with the films and stirred using a mechanical stirrer for 1 h under ice bath conditions to yield I@MLNP.
4) The siRNA was dissolved in DEPC water and then put it in the ice bath. The thin film is prepared according to the previous method. Subsequently, the film was dissolved in DEPC water mixed with siRNA and stirred with a mechanical stirrer for 1 h under ice bath conditions, resulting in the formation of I‐sR@LNP.
5) Macrophage membranes and commercially acquired siRNA were dissolved in DEPC water and left to stand in an ice bath. Following this, membranes containing DPPC, DSPE‐PEG‐2000, cholesterol, and ICG‐II were dissolved in a mixture of DEPC and stirred with a mechanical stirrer for 1 h under ice bath conditions, resulting in the formation of I‐sR@MLNP. The preparation of I‐cy5‐sR@MLNP followed the same procedures outlined above.
6) The particle size and potential of the nanoparticles were systematically monitored. The morphological state and dimensions of I@MLNP, in comparison with I‐sR@MLNP, were observed using transmission electron microscopy. Additionally, UV–vis absorption spectra of the I@MLNP, I‐sR@LNP and I‐sR@MLNP solutions were scrutinized to confirm the encapsulation of ICG‐II within the nanoparticles. Following a 14‐day period, the particle size of both nanoparticles was reassessed to evaluate the chemical stability of the formulations.
7) To assess the stability of nanomaterials in siRNA encapsulation, nanoparticles with varied siRNA concentrations were formulated. The potential leakage of siRNA from these nanoparticles was then evaluated through 3% agarose gel electrophoresis.
8) The encapsulation and drug loading rates of siRNA were assessed following established procedures in the literature.^[^ ^28^ ^]^ A homogeneous solution, incorporating 200 mL of 10% (w/v) Triton X‐100 into 200 mL of nanoparticle suspension, was created to contain free cy5‐siRNA, with the amount quantified using fluorescence spectroscopy. Subsequently, to determine the quantity of free cy5‐siRNA within the nanoparticles, another 200 mL of nanoparticle suspension was taken, diluted to 2 mL with distilled water, introduced into an ultrafilter (Vivaspin2; Sartorius Biotech, USA), and centrifuged at 7992 rpm for 10 min. The fluorescence spectrometry was employed to detect free cy5‐siRNA in the filtrate, and the encapsulation rate was calculated using the provided equation:
(3) $$\mathrm{EE}=\frac{W_{\left(\mathrm{Total}\;\mathrm{drug}\right)}-W_{\left(\mathrm{Free}\;\mathrm{drug}\right)}}{W_{\left(\mathrm{Total}\;\mathrm{drug}\right)}}\times 100\%$$

W (Total drug) signifies the overall quantity of siRNA present in the nanoparticles, while W (Free drug) represents the unbound amount of siRNA within the nanoparticles. The loading rate of siRNA was determined using the same method employed for calculating the loading rate of ICG‐II.

9) To guarantee the integrity and authenticity of the proteins present on the nanoparticles, SDS‐PAGE and WB analysis were employed to assess the protein profiles and specific proteins in the purified macrophage membranes and macrophage membrane‐encapsulated nanoparticles, respectively.

### Photothermal Property Testing of I‐sR@MLNP

I‐sR@MLNP with varying concentrations of ICG‐II underwent continuous light illumination using a 785 nm laser (0.6 W cm^−2^) for 8 min. Temperature measurements were recorded using a thermocouple. Concurrently, water, nano‐empty shells, ICG‐II, and nanoparticles with an equivalent ICG‐II concentration underwent infrared thermography experiments to provide a more intuitive assessment of the photothermal properties of I‐sR@MLNP.

### Stability Testing of I@MLNP, I‐sR@LNP, and I‐sR@MLNP

1) Following a 14‐day period, the particle size of both nanoparticles was reassessed to evaluate the chemical stability of the formulations.
2) The colloidal stability of I‐sR@MLNP in DMEM containing 10% FBS was monitored at 37 °C. One milliliter of the I‐sR@MLNP solution was centrifuged to remove the supernatant, then re‐dissolved in 1 mL of DMEM complete medium. DMEM solutions of I‐sR@MLNP, subjected to different durations, were analyzed through gel electrophoresis.

### Temperature Responsive Release of I‐sR@LNP and I‐sR@MLNP

To further validate the in vitro temperature‐triggered release of I‐sR@LNP and I‐sR@MLNP, drug release studies were conducted at 37, 42, and 46 °C. Celsius using the dialysis technique. In brief, 0.5 mL of nanoparticles were introduced into a dialysis bag (MWCO 50 kDa), which was then submerged in 30 mL of HEPES buffer medium and agitated at 60 RPM. Subsequently, 0.5 mL of the release sample was periodically extracted and replaced with an equal volume of fresh release medium at predetermined intervals. The siRNA content in the samples was determined through fluorescence spectroscopy, and the cumulative amount of released siRNA was calculated using the formula shown below:

(4) $$\mathrm{Cumulative}\;\mathrm{Release}\;\left(\%\right)=\frac{V\times C_{t}+V_{r}\times \sum C_{m}}{\mathrm{Dose}}\times 100\%$$

Here, V denotes the volume of the release medium, ct signifies the detected concentration of siRNA in the sample collected at time t, ∑Cm represents the sum of the collected samples, vr is the sampling volume used for analysis, and dose indicates the amount of siRNA added to the release medium.

### In Vitro Cell Biocompatibility of I‐sR@MLNP

The RAW264.7 cells and MDA‐MB‐231 cell lines were selected as the target cells, and MTT (3‐(4,5‐dimethylthiazol‐2‐yl)‐2,5‐diphenyltetrazolium bromide) assay was carried out using a standard protocol to verify the biocompatibility of ICG‐II. 10^5^ cells/well in logarithmic growth phase were added into 96‐well plates and incubated in an incubator at 37 °C with 5% CO_2_ for 24 h. Different concentrations of I‐sR@MLNP (0, 0.02, 0.04, 0.06, 0.08, 0.1 mg mL^−1^, respectively) were co‐cultured with the cells for 24 h. Each well was washed with PBS and 10 µL of MTT solution (5 mg mL^−1^). After 4 h of incubation, the medium containing MTT solution was removed. 150 µL of DMSO was added to each well and shaken for 15 min to dissolve the crystals completely. Finally, the absorbance at 490 nm of each well was measured on an enzyme labeller. In the experiment, six replicate wells were set for each concentration to calculate the cell survival rate.

### Targeting Validation of I‐sR@MLNP

To confirm the targeting capability of I‐sR@MLNP, SDS‐PAGE analysis was conducted, and the protocol for SDS‐PAGE analysis is outlined as follows. Initially, total membrane proteins were extracted from the samples using a total cellular protein extraction kit. Subsequently, the total protein content was quantified using a BCA kit. The extracted proteins were then subjected to electrophoresis on a 10% Bis‐Tris 10‐well mini‐gel in running buffer using a Bio‐Rad electrophoresis system. Following electrophoretic separation, PVDF membranes were employed for membrane transfer, followed by a 1‐h blocking step. Antibodies were subsequently incubated, and the gel was finally imaged.

1) The presence of VCAM‐1 on MDA‐MB‐231 cell membranes was determined using SDS‐PAGE gel electrophoresis and WB analysis.
2) The presence of the same target protein, integrin α4, on I‐siRNA‐MLNP as on macrophages was determined using SDS‐PAGE gel electrophoresis and WB analysis.

### Immunoprecipitation Experiments to Verify Protein Interactions

To verify the interaction of two proteins, integrin α4 on macrophage membranes and VCAM‐1 in MDA‐MB‐231 cells, immunoprecipitation experiments (CO‐IP) were performed. Cells were centrifuged and lysed at 4 °C after 12 h of I‐siRNA‐MLNP treatment. Anti‐GFP primary antibody (Abmart, M20004M) was then incubated with the lysates overnight at 4 °C to form immune complexes with integrin α4 and VCAM‐1 proteins. Protein A/ g agarose beads were coated and incubated at 4 °C for 2 h. Subsequently, SDS‐PAGE gel electrophoresis and WB analysis were performed.

### In Vitro Cellular Uptake and Lysosomal Escape

1) LNP (including ICG‐II and Cy5‐siRNA) and MLNP (including ICG‐II and Cy5‐siRNA) was co‐incubated with MDA‐MB‐231 cells for 2 h, 4 h, and 8 h, respectively. Subsequently, the cellular uptake of LNP and MLNP was investigated through flow cytometric analysis.
2) MDA‐MB‐231 cells were seeded in confocal dishes at a density of 5 × 10^4^ cells per dish. The cells were then exposed to 0.5 mL of various nanoparticles (I‐Cy5‐sR@LNP and I‐Cy5‐sR@MLNP) for 8 h. Following the treatment, cells were extracted from the medium, rinsed three times with PBS, and fixed with 10% paraformaldehyde for 15 min. The cellular uptake of nanoparticles was visualized using an Olympus fluorescence microscope system.
3) Having identified the optimal uptake time, I‐Cy5‐sR@MLNP was introduced to MDA‐MB‐231 cells in confocal dishes. The treated cells underwent incubation without light and with light exposure (785 nm, 0.6 W cm^−2^, 5 min). Following light exposure, the cells were further incubated for 0.5 h, stained using a Hoechst with lysosomal probe (Lyso‐Tracker Green), washed three times with PBS, and fixed with 10% paraformaldehyde for 15 min. The lysosomal escape of the nanoparticles was then visualized using an Olympus fluorescence microscope system.

### In Vitro Gene Silencing

Gene silencing assays were analyzed by qPCR and WB. First, MDA‐MB‐231 cells were inoculated in 24‐well plates with 5 × 10^4^ cells per well. Subsequently, the cells were divided into six groups, namely, control (group 1), sR@Lip3000 (group 2), I‐sR@MLNP (group 3), I@LNP+Laser (group 4), I@MLNP+Laser (group 5), and I‐sR@MLNP+Laser (group 6). the control group and sR@Lip3000 groups were used as negative and positive controls, respectively. A 5 min laser was used to induce siRNA release while applying photothermal effects. Cells were harvested after 24 h of incubation and mRNA and protein were extracted. qPCR Detecting System and WB analysis were used to detect mRNA and protein expression levels of HSP70.

### Single‐Treatment Cytotoxicity and Necrosis

1) MDA‐MB‐231 cells were categorized into six groups (PBS, Laser, I‐sR@MLNP, I@MLNP+Laser, and I‐sR@MLNP+Laser). The final siRNA concentration was 100 nm and the concentration of ICG‐II was 50 µg mL^−1^. Photothermal cytotoxicity investigations were conducted using a 785 nm pulsed laser at 0.6 W cm^−2^ for 5 min, employing the MTT method as previously described. Cell survival rates were compared among the six groups under identical light conditions at 785 nm. Apoptotic cells were treated with Annexin V‐FITC Apoptosis Detection Kit, detected by flow cytometry and the results were analyzed.
2) Expression of Caspase 3 protein and Bax protein was analyzed in different treated cell samples using SDS‐PAGE gel electrophoresis and WB.
3) In order to observe the cytotoxicity more visually, MDA‐MB‐231 cells were analyzed by live‐dead cell staining. The six groups of MDA‐MB‐231 cells with different treatments were incubated at 37 °C for 4 h and washed twice with PBS. Different groups of MDA‐MB‐231 cells were stained with calcein AM (2 µm) and PI (8 µm) solutions for 30 min, washed with PBS, and imaged by inverted fluorescence microscope.

### Evaluation of Gene Silencing Effects of siRNAs at Different Times

MDA‐MB‐231 cells were seeded in 24‐well plates and treated with I‐sR@MLNP (containing 50 µg mL^−1^ ICG‐II) after a 24 h incubation. Following an additional 8 h, the treated cells were irradiated for 5 min using a 785 nm laser (0.6 W cm^−2^). The final concentration of siRNA in the experiments was 100 nm. Subsequently, cells were collected at different time points of incubation (4, 8, 12, 18, 24, 36, 48, 72 h), and both mRNA and protein of HSP70 were extracted. qPCR Detecting System and WB analysis were employed to assess the expression levels of mRNA and protein of HSP70, and the data were subjected to statistical analysis.

### Dual‐Phase Treatment Cytotoxicity and Necrosis

MDA‐MB‐231 cells were cultured in 24‐well plates and categorized into five groups (control, I@MLNP+Laser‐1, I@MLNP+Laser‐2, I‐sR@MLNP+Laser‐1, and I‐sR@MLNP+Laser‐2). Here, Laser‐1 (L‐1) denotes a single light exposure, and Laser‐2 (L‐2) represents double consecutive light illuminations. The interval between the two light exposures was 36 h. Subsequently, MTT assay, flow cytometric analysis, and live‐dead cell staining were conducted on the various groups of treated cells to evaluate cell viability following the previously outlined experimental protocols. The expression of HSP70 protein, Caspase 3 protein, and Bax protein in various treated cell samples was analyzed through SDS‐PAGE gel electrophoresis and WB.

### In Vivo Biodistribution and the Assessment of MPTT

1) All animal experiments were conducted in accordance with the guidelines for the use of the Animal Ethics and Welfare Committee of Nanjing University. A mouse tumor model was established by inoculating MDA‐MB‐231 cells in the right axillary fossa position of Balb/c nude mice. In vivo experiments commenced when the tumor size reached 80±10 mm^3^. The mice were then randomly divided into two groups. Different groups of mice were administered I‐Cy5‐sR@LNP (50 µg mL^−1^, 200 µL) and I‐Cy5‐sR@MLNP (50 µg mL^−1^, 200 µL) via the tail vein, respectively, and were subsequently imaged using a near‐infrared fluorescence imaging system at 4, 8, 12, and 24 h. The tumor uptake time was determined using the NIRF system. Following the identification of the optimal tumor uptake time in vivo, at 12 h, a 785 nm laser was employed to irradiate the tumor site for 5 min, and an IR thermal imaging camera was utilized to record the temperature change at the tumor site over this duration.
2) To assess the PTT efficacy of I‐sR@MLNP, nude mice bearing MDA‐MB‐231 tumors were randomly assigned to six groups: 1) control group; 2) single laser group; 3) tail vein injection of ICG‐II and laser; 4) tail vein injection of I@LNP and laser; 5) tail vein injection of I@MLNP and laser; 6) tail vein injection of I‐sR@LNP and laser; 7) tail vein injection of I‐sR@MLNP and laser. For PTT assessment, a 785 nm laser with a power density of 0.6 W cm^−2^ was used, and a single irradiation session lasted for 5 min. Initially, various drugs were injected, and after 12 h, the primary irradiation was conducted to release siRNA predominantly. The second drug injections were administered 24 h post‐irradiation, followed by a second irradiation after an additional 12 h. The treatment evaluation period extended for 14 days. After treatment period, five mice from each group were randomly euthanized, and tumor tissues were collected. The remaining mice in each group were observed for survival for a duration of 30 days.

### Tissue Immunofluorescence

Tumor tissues were embedded and then frozen in optimal cutting temperature (OCT) medium. For target detection, sections underwent permeabilization (0.5% Triton X‐100 in PBS, 10 min) followed by blocking with 3% BSA. Primary antibodies targeting HSP70 (Abcam #ab2787, 1:100) and KI67 (Abcam #ab279653, 1:100) were applied at 4 °C overnight respectively. Fluorophore‐conjugated secondary antibodies were incubated for 1 h at room temperature, and nuclei were visualized with DAPI counterstaining. The TUNEL immunofluorescence staining procedure was performed strictly according to the manufacturer's protocol (Beyotime, #C1089). Images were acquired via confocal microscopy.

### In Vivo Safety Assessment

To investigate the safety of the constructed nanoparticles and the treatment modality, healthy nude mice were injected intravenously with saline (control), single light (control), ICG‐II (control), I@LNP, I@MLNP, I‐sR@LNP and I‐sR@MLNP. the injected dose was 10 mg mL^−1^ transformed to ICG‐II and 1.2 mg kg^−1^ transformed to siRNA. 2 days later venous blood was collected from anaesthetized nude mice for biochemical analysis. Subsequently, major tissues including heart, liver, brain, lung, and kidney were collected and stained with H&E for subsequent analysis.

### Data Analysis

All data are expressed as mean ± standard deviation (SD). Student's t‐test was used for statistical evaluation. Differences between groups were considered statistically significant when the probability (p) was less than 0.05.

## Conflict of Interest

Huiming Cai is CEO of Nuoyuan Medical Devices.

## Supporting information



Supporting Information

## Data Availability

The data that support the findings of this study are available from the corresponding author upon reasonable request.
