# Correction to “Strategic Timing of Gene Silencing: Cellular Kinetics‐Based Administration of siRNA for Optimized Photothermal Cancer Treatment”

**DOI:** 10.1002/advs.202518623

**Published:** 2025-10-15

**Authors:** Tianliang Fang, Li Li, Ziyad Tariq Muhseen, Lucas A. Lane, Huiming Cai, Christopher J. Butch, Yiqing Wang

https://doi.org/10.1002/advs.202510802

Correction 1

In Figure S31 (Supporting Information), incorrect merge data were included for the 48 h time point (duplicating the 24 h merge). The individual channel data was correct, and the correct merge image is now included.


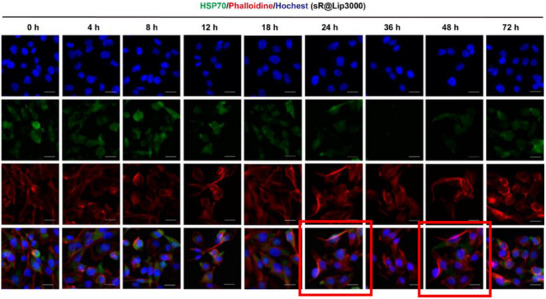


Original Figure S31 (Supporting Information). The CLSM images in MDA‐MB‐231 cells treated with siRNA‐Lipo3000 at different times. Scale bar: 50 µm.


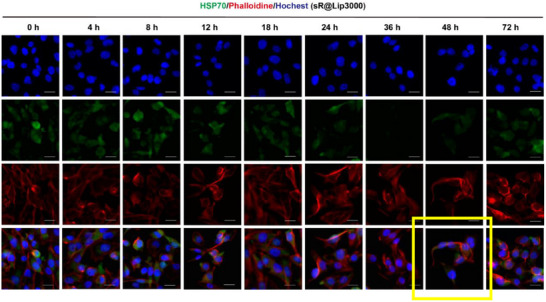


Revised Figure S31 (Supporting Information). The CLSM images in MDA‐MB‐231 cells treated with siRNA‐Lipo3000 at different times. Scale bar: 50 µm.

Correction 2

In Figure S44 (Supporting Information), liver cell H&E images were included in two rows in the figure, and incorrectly labelled as Kidney cells in the second row. The appropriate kidney cell images are now included.


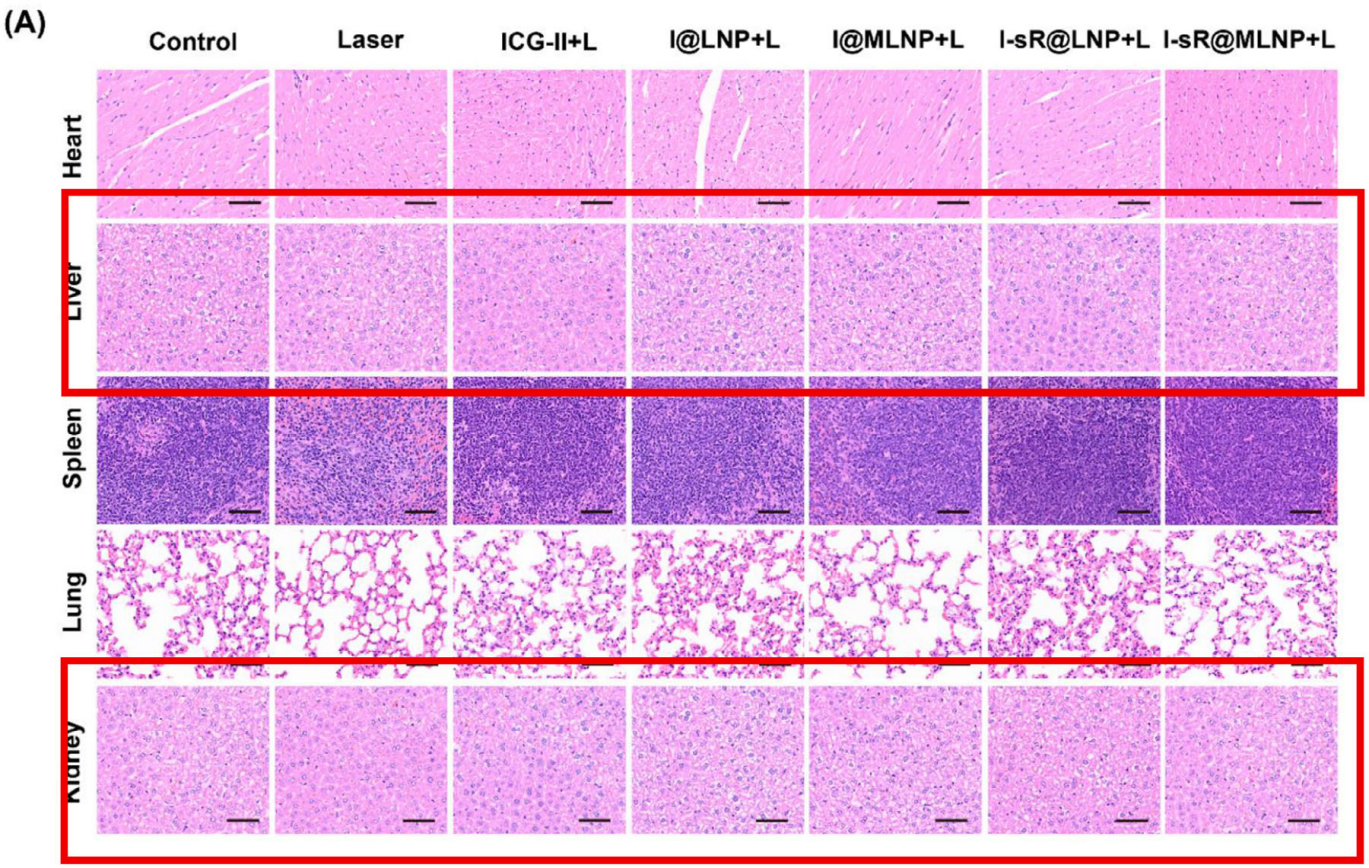


Original Figure S44 (Supporting Information). A) The H&E staining of different organs after 20‐day post‐injection. Note: Scale bars: 100 µm. B) The data analysis of blood biochemical indexes in different groups (*n *= 5).


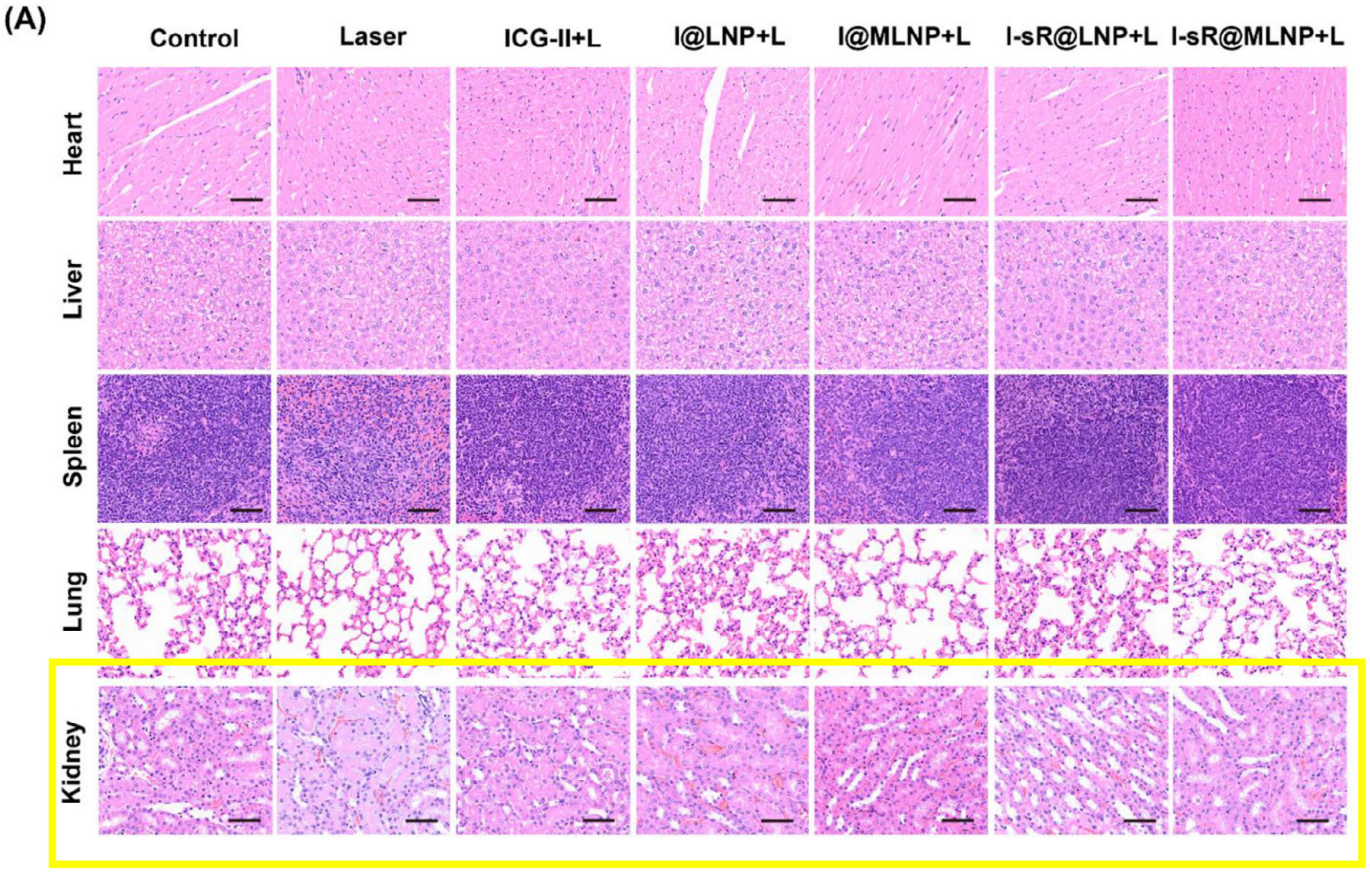


Revised Figure S44 (Supporting Information). A) The H&E staining of different organs after 20‐day post‐injection. Note: Scale bars: 100 µm. B) The data analysis of blood biochemical indexes in different groups (*n *= 5).

