# Supplementary material for: Strategic Timing of Gene Silencing: Cellular Kinetics‐Based Administration of siRNA for Optimized Photothermal Cancer Treatment
Source: Adv Sci (Weinh). 2025 Sep 6;12(44):e10802. doi: 10.1002/advs.202510802 (PMC12667499; doi:10.1002/advs.202510802)
Supplement: Supplementary file 1 — Supporting Information [file ADVS-12-e10802-s001.docx]

*Supporting Information

Strategic Timing of Gene Silencing: Cellular Kinetics-Based Administration of siRNA for Optimized Photothermal Cancer Treatment

Tianliang Fang, ^†^ Li Li, ^†^ Ziyad Tariq Muhseen, Lucas A. Lane*, Huiming Cai,* Christopher J. Butch,* and Yiqing Wang*


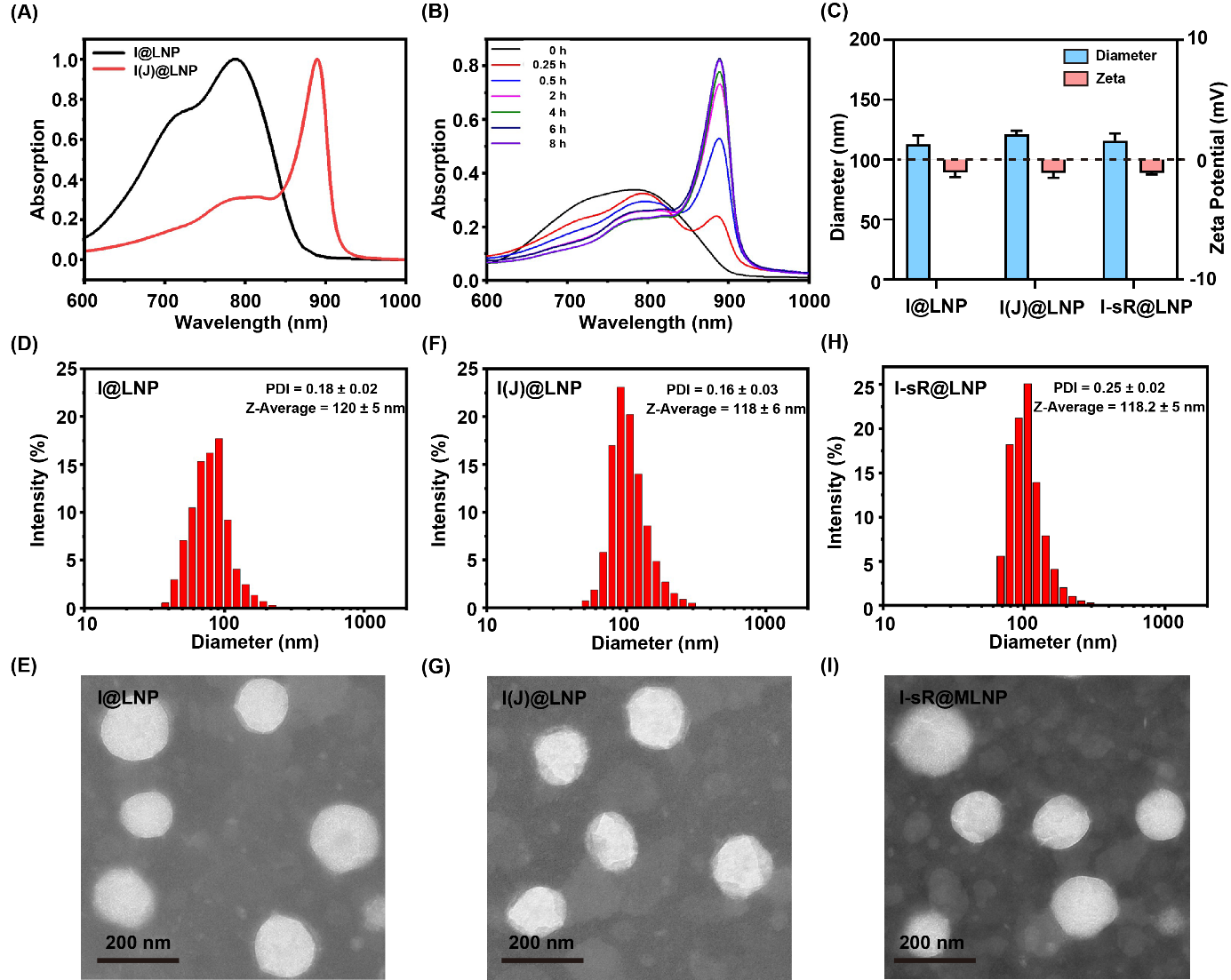


**Fig. S1.** (A) The normalized absorption of I@LNP and I(J)@LNP. (B) The absorption of I(J)@LNP at different times. (C) The diameters and zeta potentials of I@LNP, I(J)@LNP and I-sR@LNP (*n=3*). (D) The DLS measurement of I@LNP. (E) The TEM data of I@LNP. (F) The DLS measurement of I(J)@LNP. (G) The TEM data of I(J)@LNP. (H) The DLS measurement of I-sR@LNP. (I) The TEM data of I-sR@LNP.


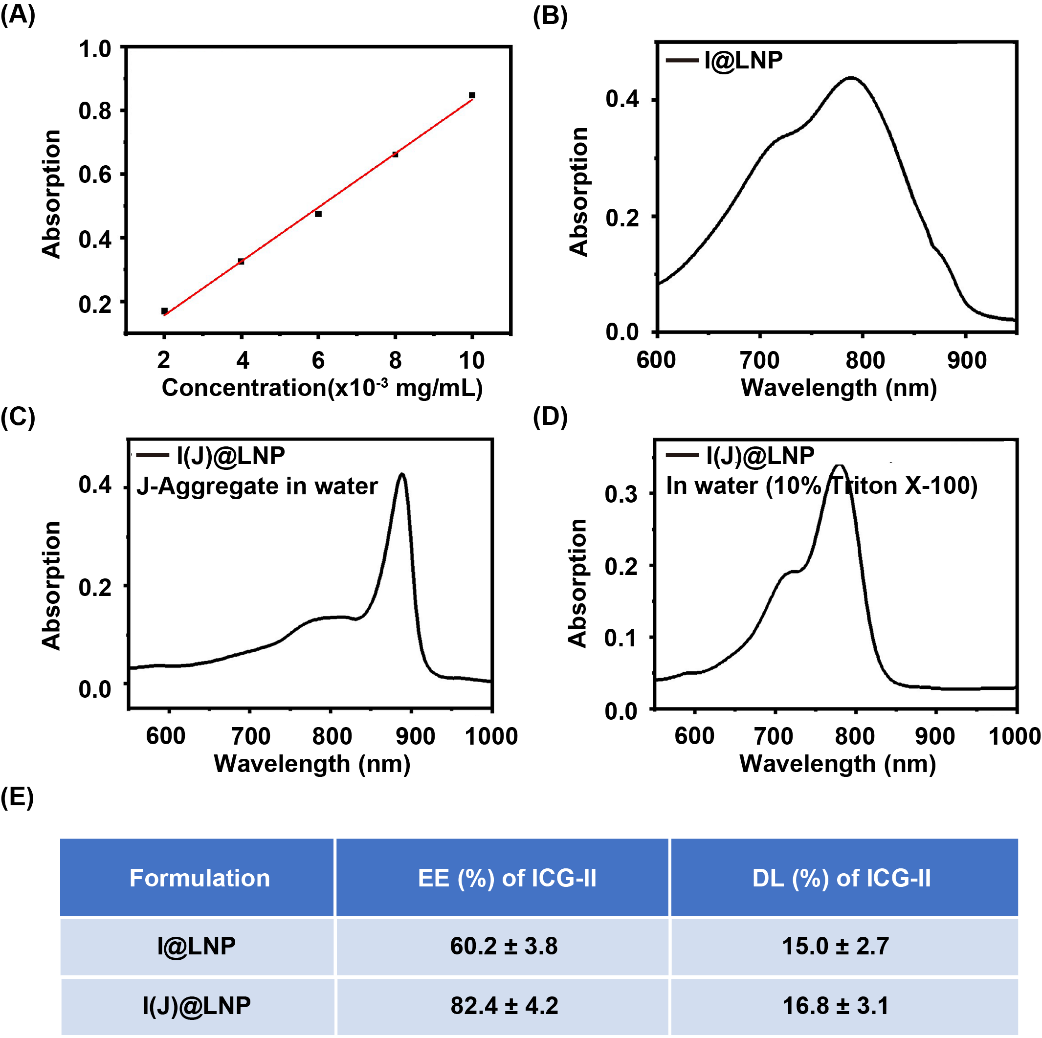


**Fig. S2.** (A) The UV absorption spectral standard curve of ICG-II in DMSO; (B) The absorption of I@LNP; (C) The absorption of I(J)@LNP; (D) The absorption of I(J)@LNP in water including 10% Triton X-100; (E) The encapsulation efficiency (EE) and drug loading efficiency (DL) of I@LNP and I(J)@LNP.


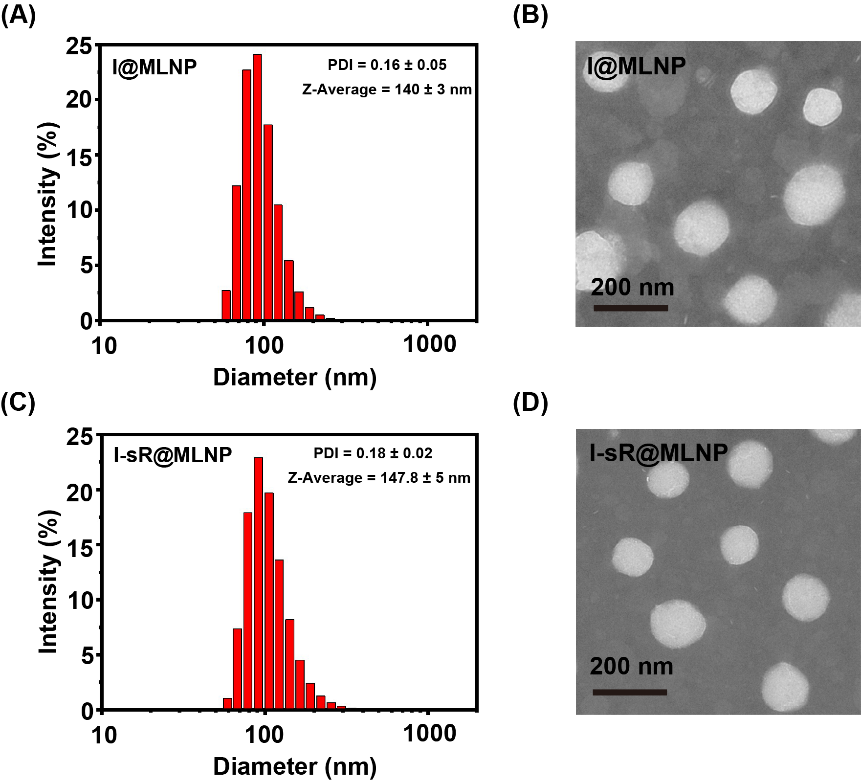


**Fig. S3.** (A) The DLS measurement of I@MLNP. (B) The TEM data of I@MLNP. (C) The DLS measurement of I-sR@MLNP. (D) The TEM data of I-sR@MLNP.


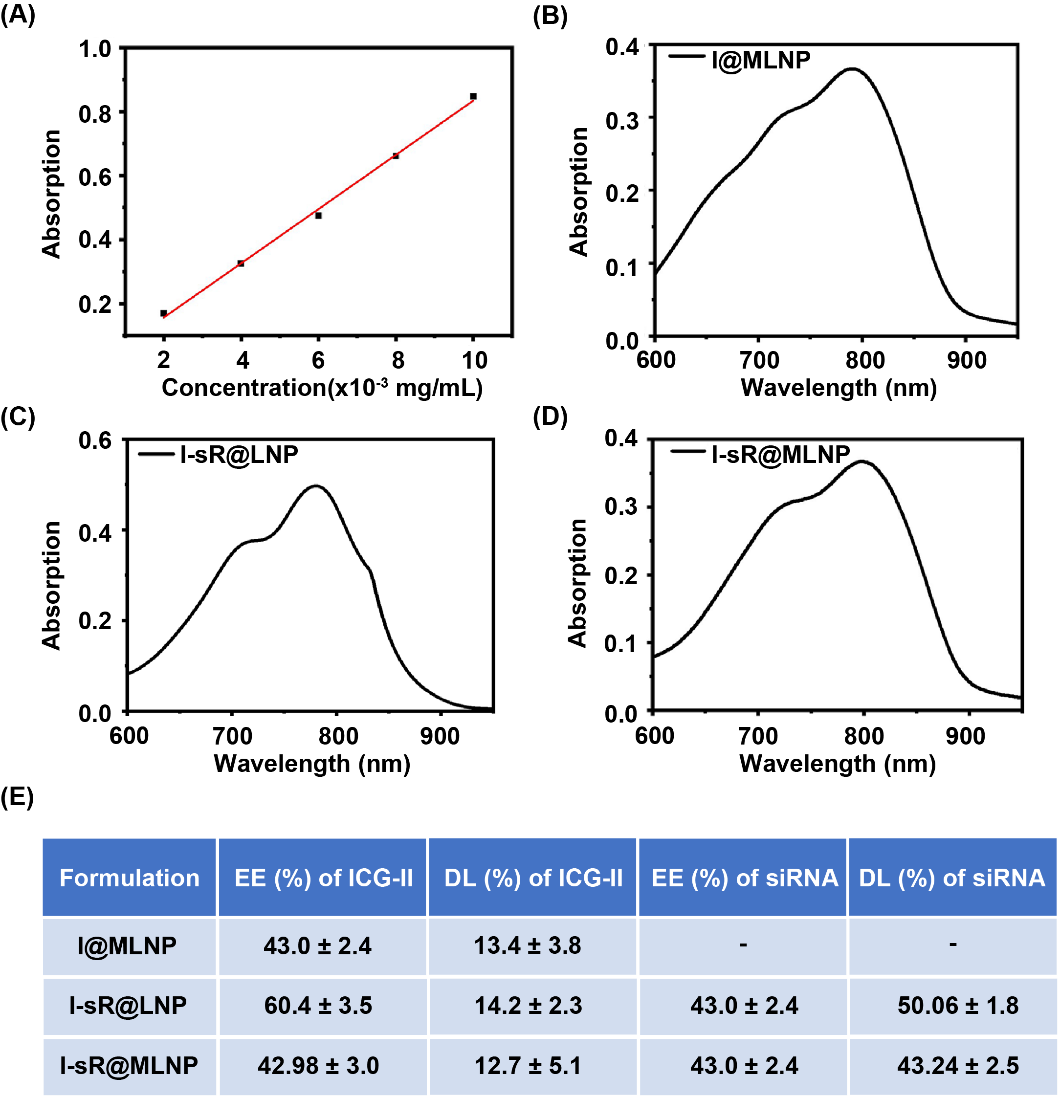


**Fig. S4.** (A) The UV absorption spectral standard curve of ICG-II in DMSO. (B) The absorption of I@MLNP. (C) The absorption of I-sR@LNP. (D) The absorption of I-sR@MLNP. (E) The EE and DL of I@MLNP, I-sR@LNP and I-sR@MLNP.


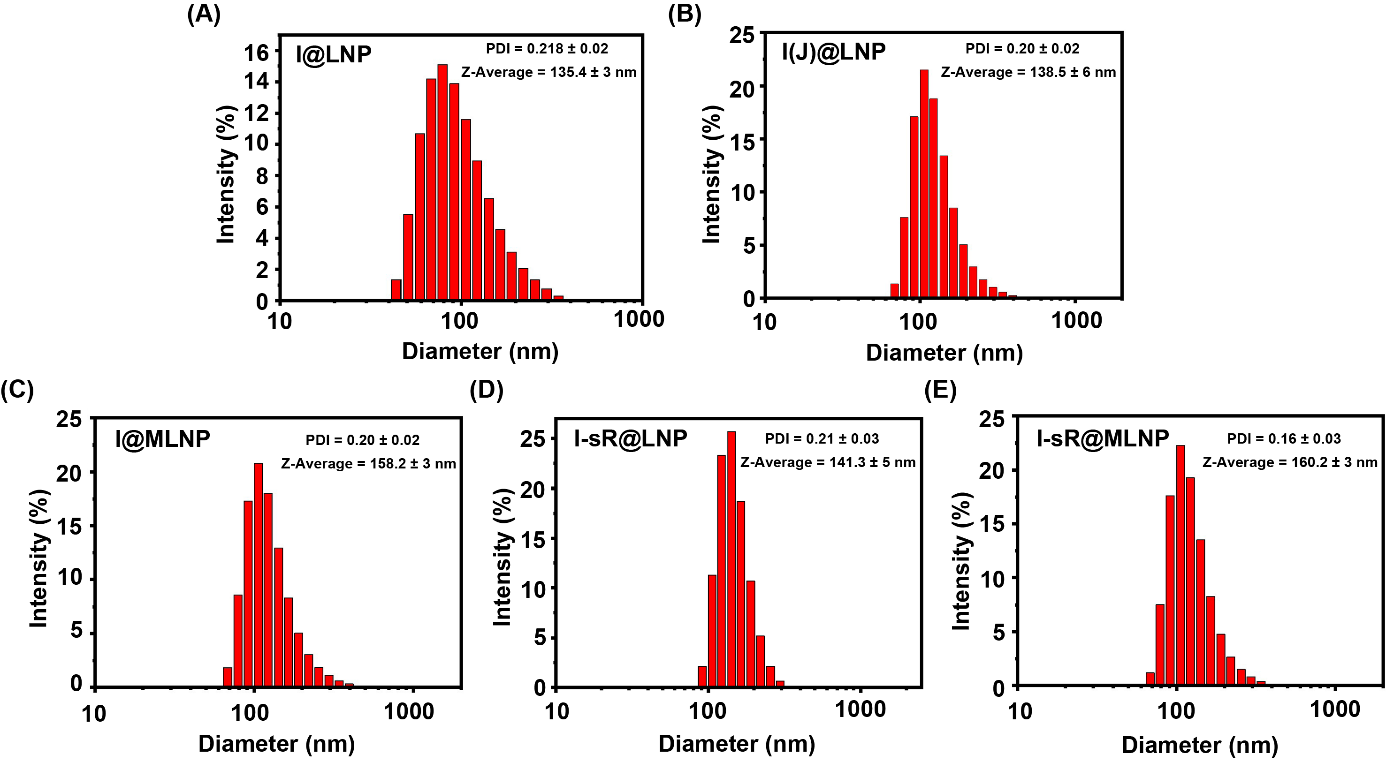


**Fig. S5.** (A) The DLS measurement of I@LNP after 14 days. (B) The DLS measurement of I(J)@LNP after 14 days. (C) The DLS measurement of I@MLNP after 14 days. (D) The DLS measurement of I-sR@LNP after 14 days. (E) The DLS measurement of I-sR@MLNP after 14 days.


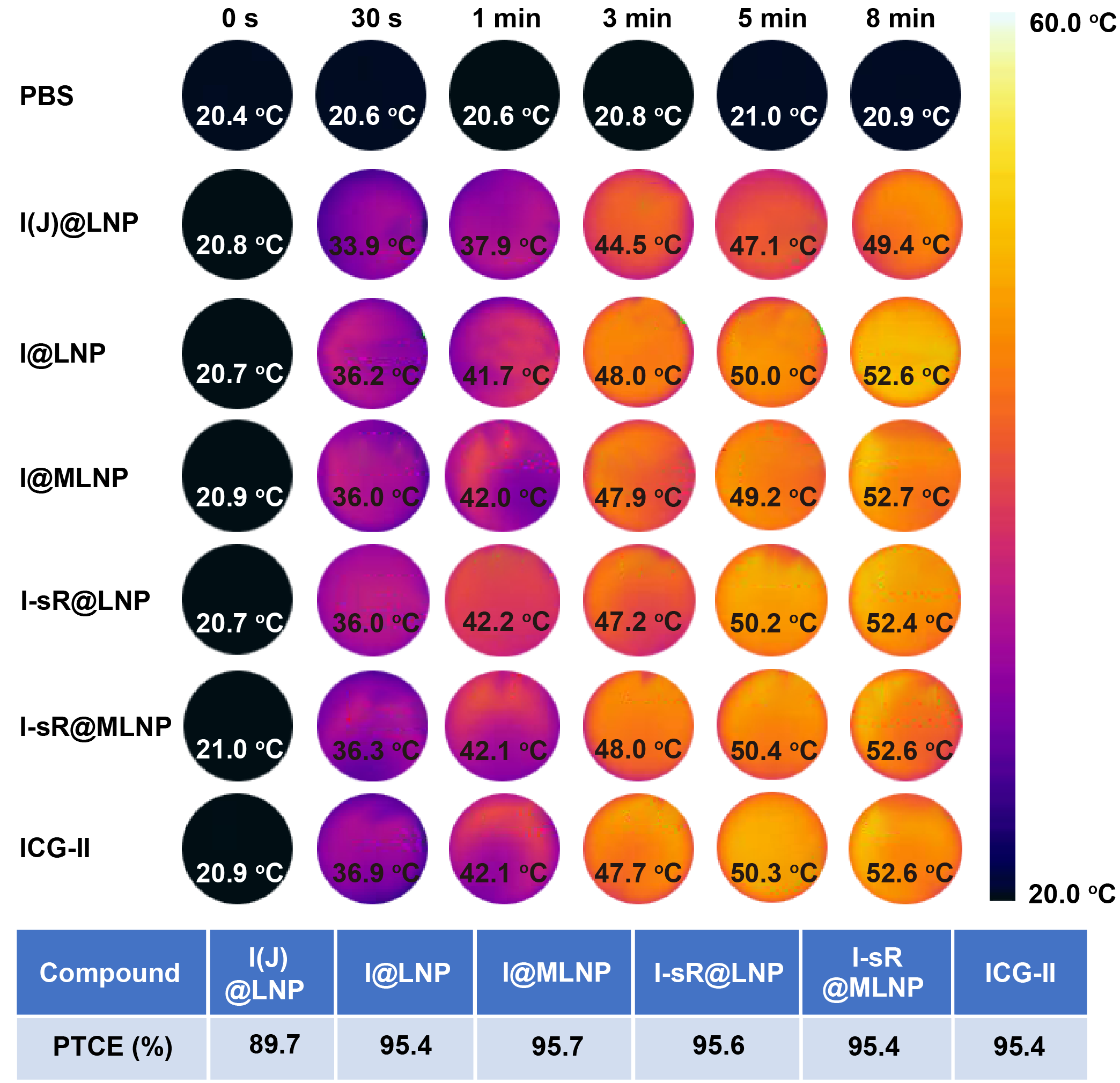


**Fig. S6.** The IR thermal images and PTCE of PBS, I(J)@LNP, I@LNP, I@MLNP, I-sR@LNP, I-sR@MLNP and ICG-II (80 μg/mL). All nanoparticles contain ICG-II at a concentration of 80 μg/mL.


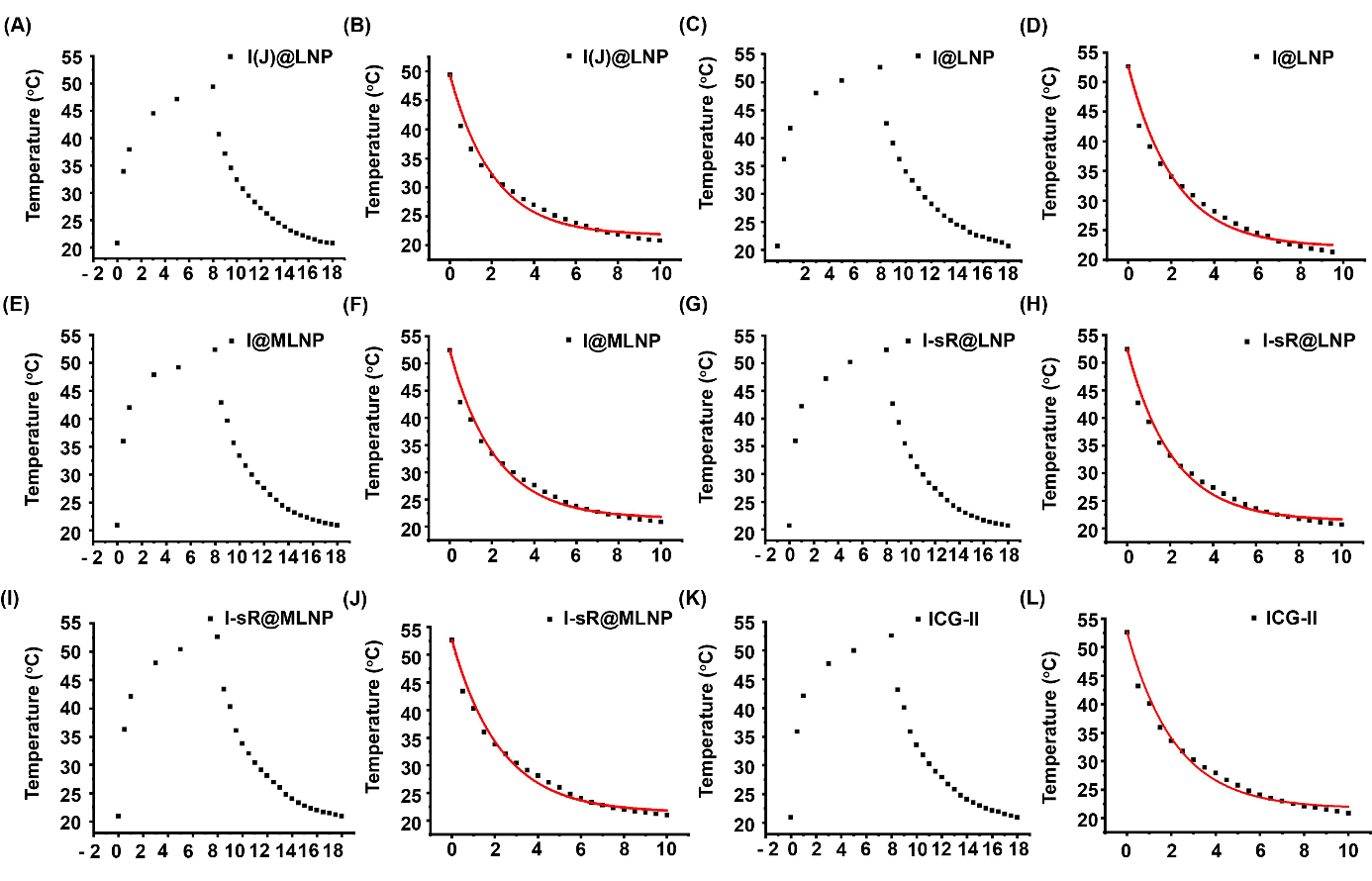


**Fig. S7.** (A) The temperature of I(J)@LNP under laser. (B) The Non-linear fitted curve of I(J)@LNP after passive cooling. (C) The temperature of I@LNP under laser. (D) The Non-linear fitted curve of I@LNP after passive cooling. (E) The temperature of I@MLNP under laser. (F) The Non-linear fitted curve of I@MLNP after passive cooling. (G) The temperature of I-sR@LNP under laser. (H) The Non-linear fitted curve of I-sR@LNP after passive cooling. (I) The temperature of I-sR@MLNP under laser. (J) The Non-linear fitted curve of I-sR@MLNP after passive cooling. (K) The temperature of ICG-II (80 μg/mL) under laser. (L) The Non-linear fitted curve of ICG-II (80 μg/mL) after passive cooling. All nanoparticles contain ICG-II at a concentration of 80 μg/mL. The laser is 785 nm and the laser power density is 1.0 W/cm^2^.


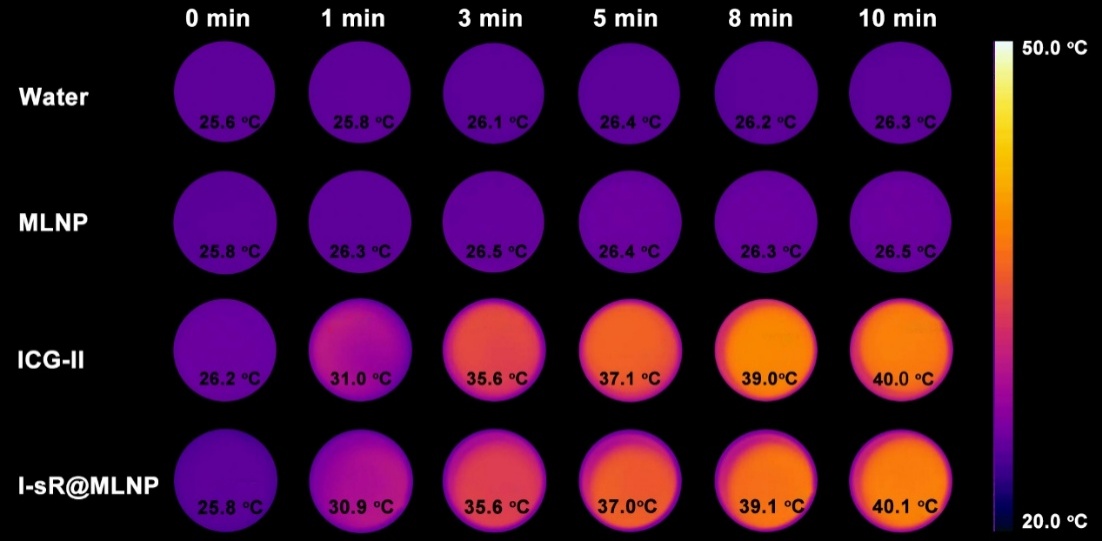


**Fig. S8.** IR thermal images of solutions of water, MLNP, ICG-II (50 μg/mL), and I-sR@MLNP (including 50 μg/mL) under 785 nm laser light (0.3 W/cm^2^).


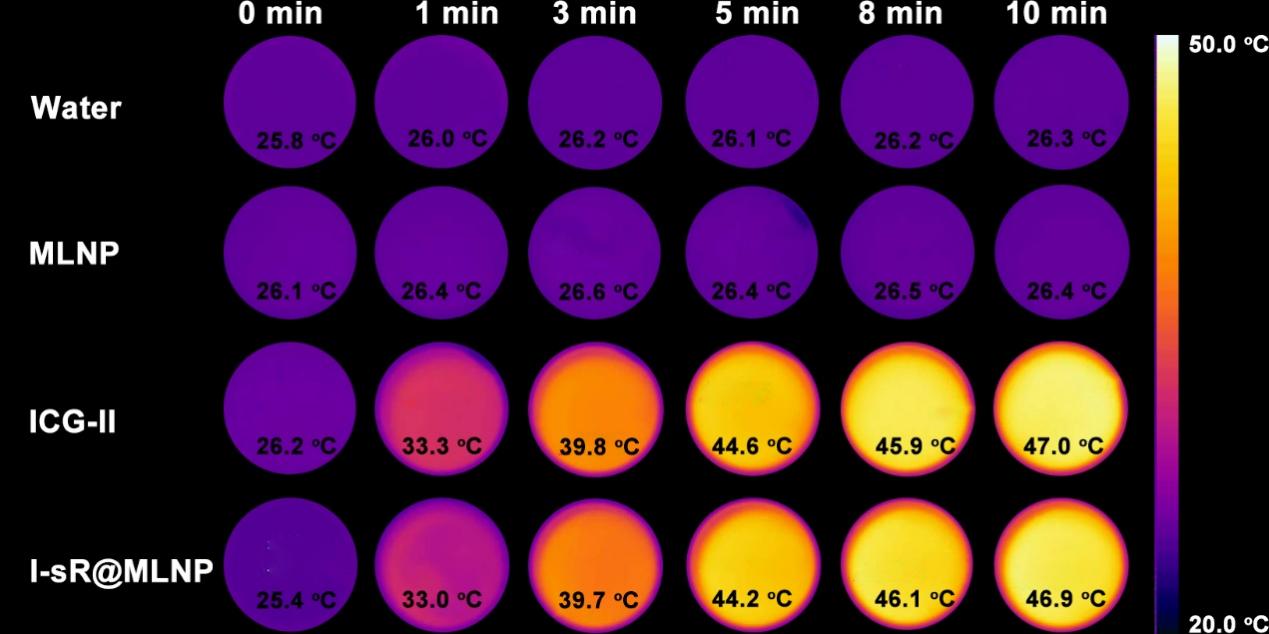


**Fig. S9.** IR thermal images of solutions of water, MLNP, ICG-II (50 μg/mL), and I-sR@MLNP (including 50 μg/mL) under 785 nm laser light (0.6 W/cm^2^).


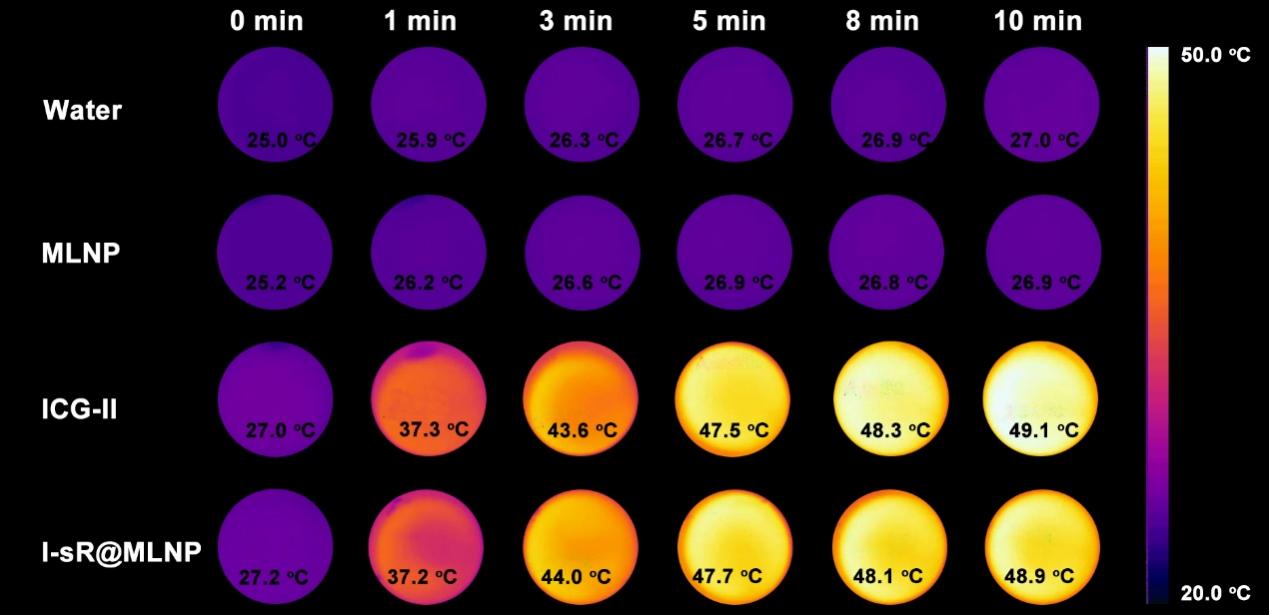


**Fig. S10.** IR thermal images of solutions of water, MLNP, ICG-II (50 μg/mL), and I-sR@MLNP (including 50 μg/mL) under 785 nm laser light (0.8 W/cm^2^).


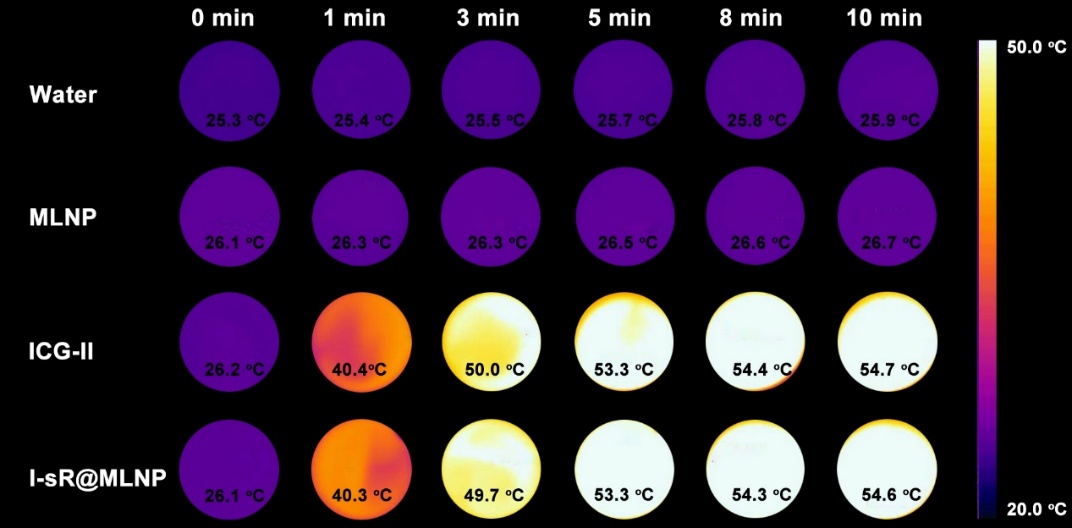


**Fig. S11.** IR thermal images of solutions of water, MLNP, ICG-II (50 μg/mL), and I-sR@MLNP (including 50 μg/mL) under 785 nm laser light (1.0 W/cm^2^).


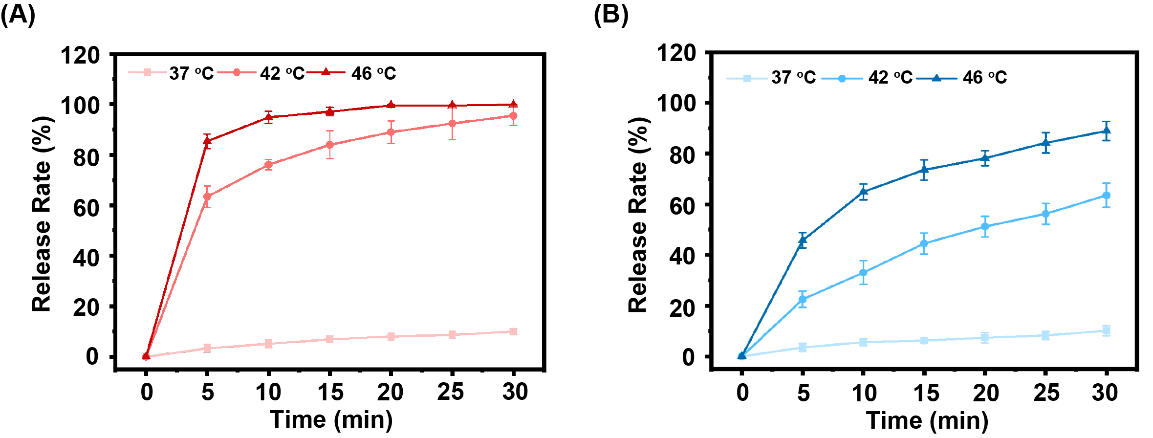


**Fig. S12.** (A) The siRNA release rates of I-sR@LNP (*n=3*); (B) The ICG-II released rates of I-sR@LNP (*n=3*).


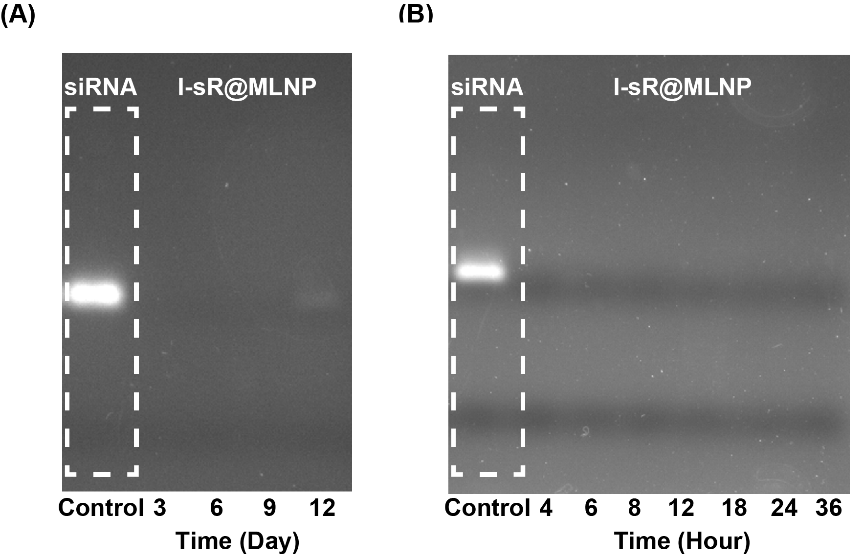


**Fig. S13.** (A) The stability of I-sR@MLNP in PBS; (B) The stability of I-sR@MLNP in DMEM (10% FBS).


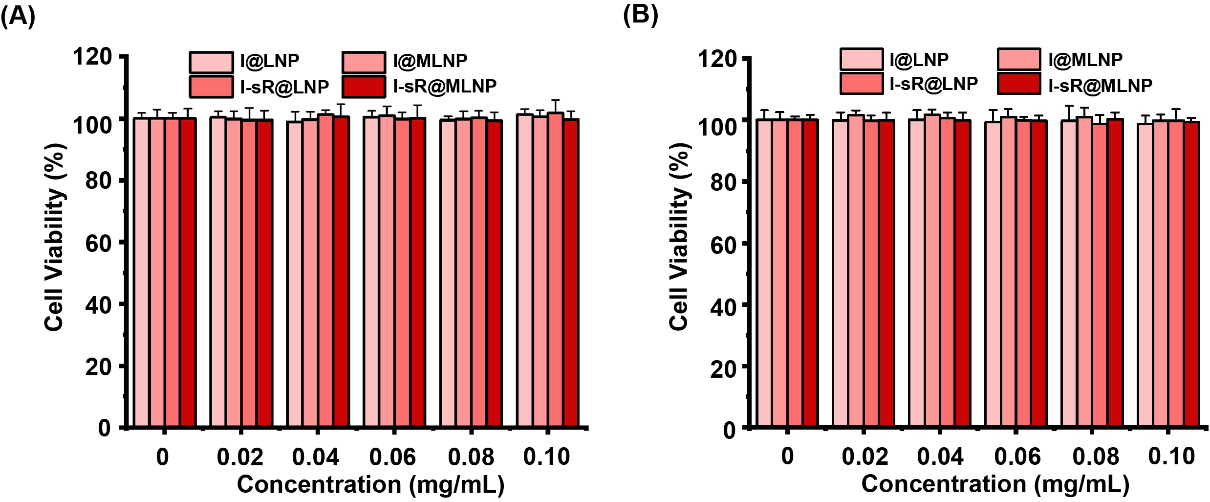


**Fig. S14.** (A) The cytotoxicity assay of nanoparticles without laser by MTT assays in RAW246.7 cells (*n=3*); (B) The cytotoxicity assay of nanoparticles without laser by MTT assays in MDA-MB-231 cells (*n=3*).


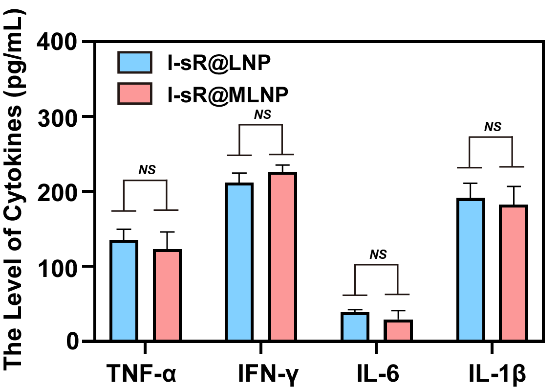


**Fig. S15.** Serum levels of TNF-α, IFN-γ, IL-6, and IL-1β in mice administered I-sR@LNP or I-sR@MLNP by ELISA (*n*=4);


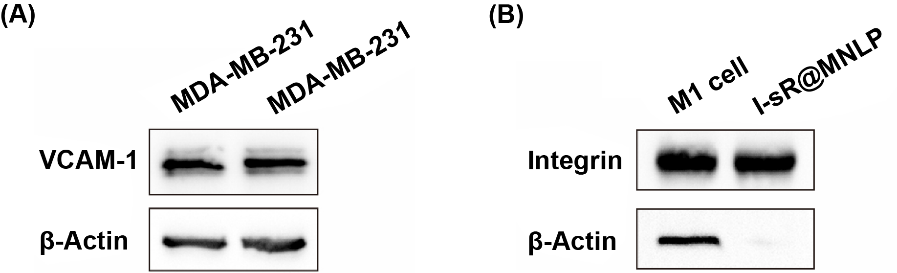


**Fig. S16.** (A) The WB analysis on the VCAM-1 level in MDA-MB-231 cells; (B) The WB analysis on the integrin α4 level of macrophage and I-sR@MLNP.


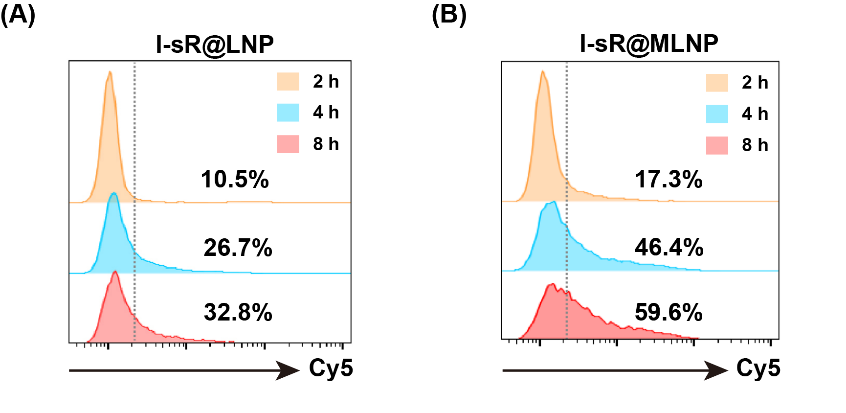


**Fig. S17.** (A) The flow analysis of cellular uptake in MDA-MB-231 cells with I-Cy5-sR@LNP at different times. (B) The flow analysis of cellular uptake in MDA-MB-231 cells with I-Cy5-sR@MLNP at different times.


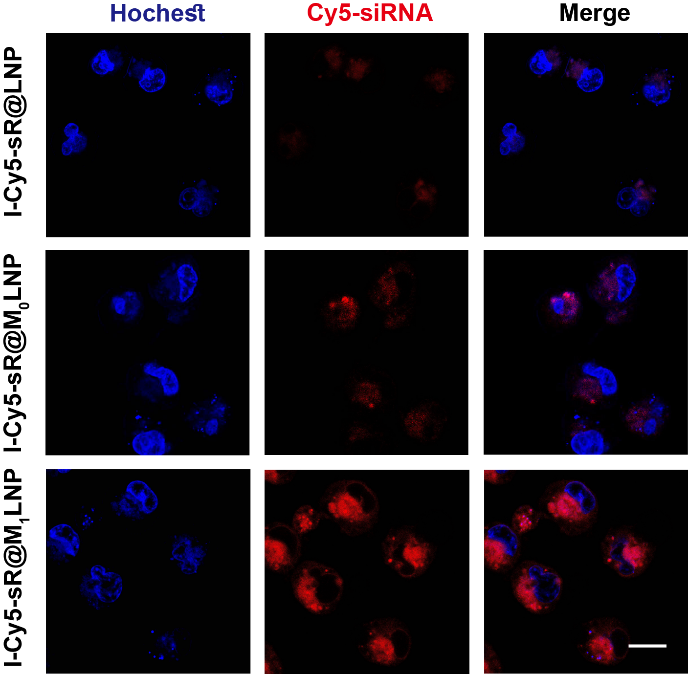


**Fig. S18.** The CLSM images of cell uptake in MDA-MB-231 cells with different approaches after 8h. Scale bar: 30 μm.


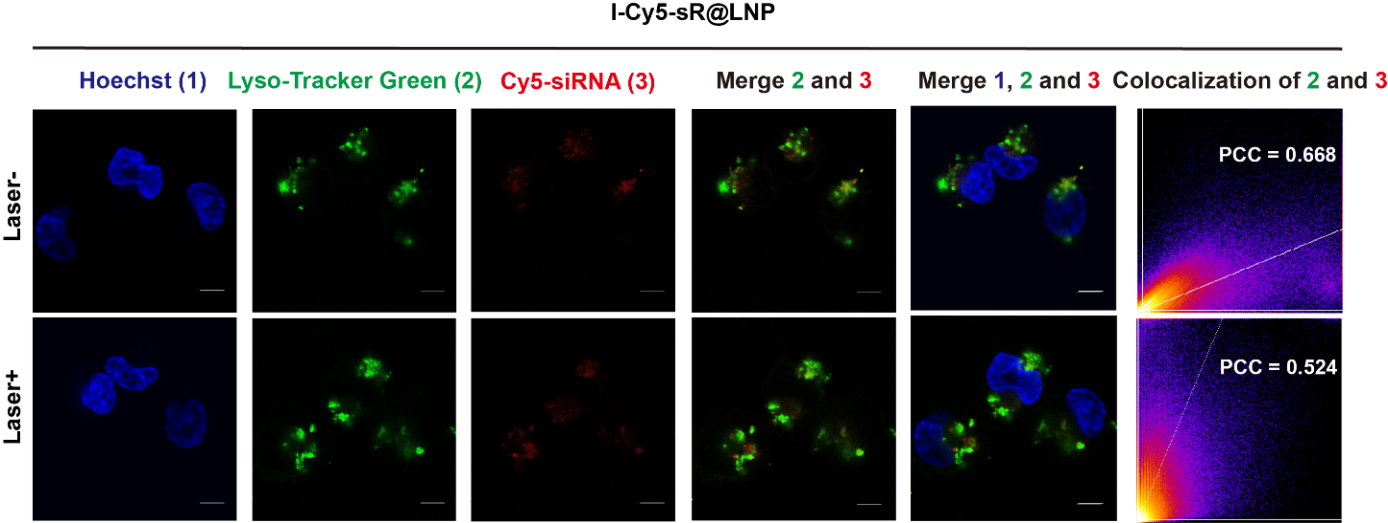


**Fig. S19.** The CLSM images of MDA-MB-231 cells with I-Cy5-sR@LNP; Channel 1 is nuclear staining with Hoechst. Channel 2 is lysosomal staining with a lysosomal green commercial probe. Channel 3 is cells treated with I-Cy5-sR@LNP. Scale bar: 20 μm. Note: The laser is 785 nm and the density of the laser is 0.6 W/cm^2^.


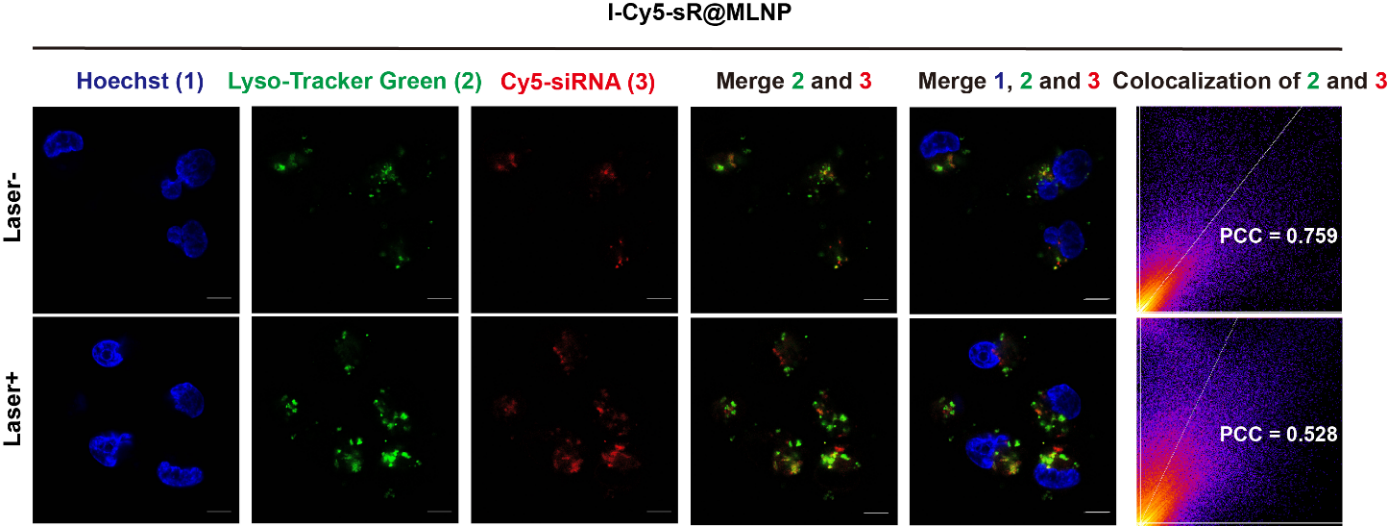


**Fig. S20.** The CLSM images of MDA-MB-231 cells with I-Cy5-sR@MLNP; Channel 1 is nuclear staining with Hoechst. Channel 2 is lysosomal staining with a lysosomal green commercial probe. Channel 3 is cells treated with I-Cy5-sR@MLNP. Scale bar: 20 μm. Note: The laser is 785 nm and the density of the laser is 0.6 W/cm^2^.


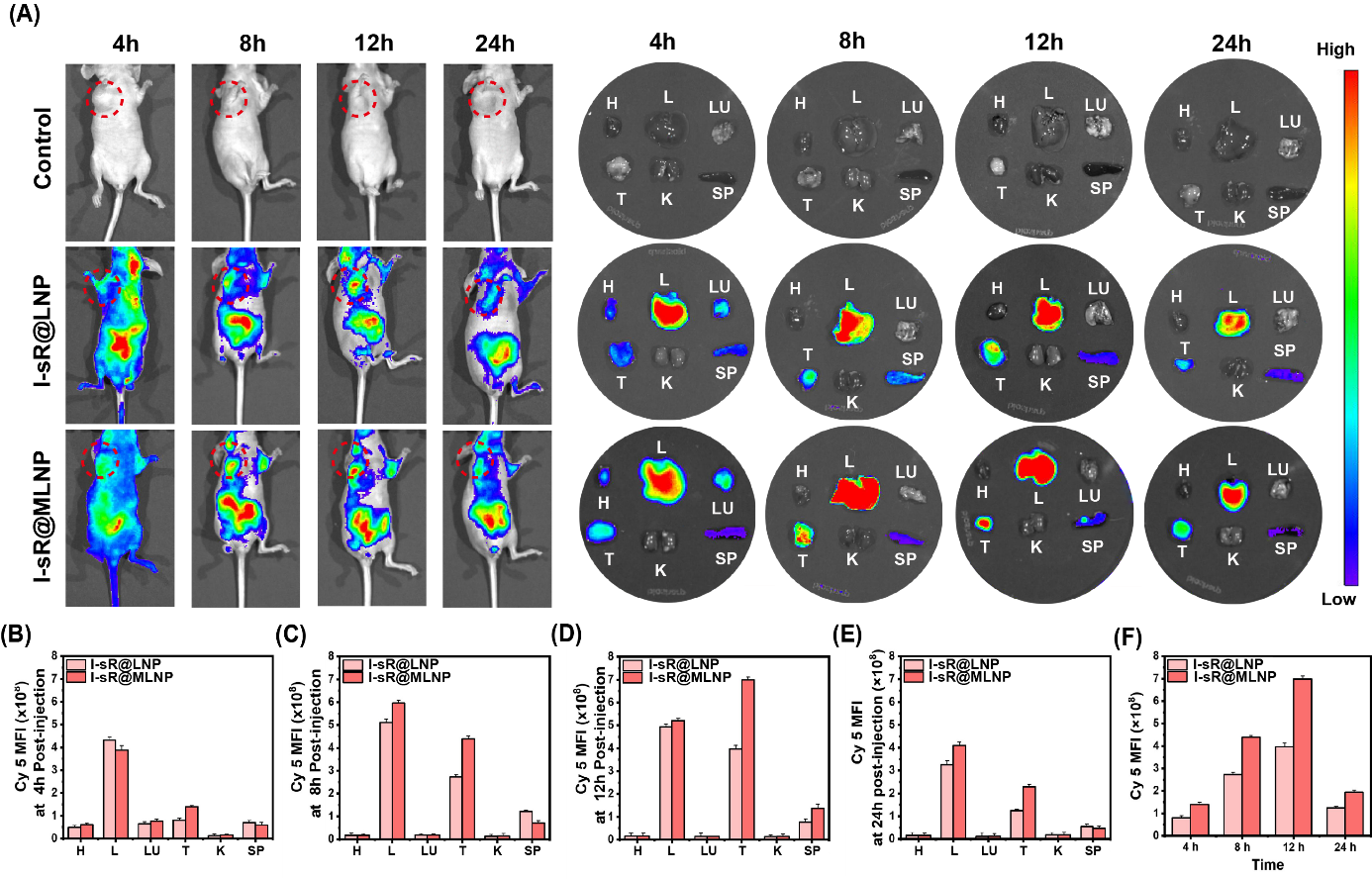


**Fig. S21.** (A)The systemic fluorescent imaging of mice at different time points after administration of physiological saline, and various samples containing cy5-siRNA. (B)-(E) The average fluorescence intensity of organ tissues at different times (*n=3*); (F) The average fluorescence intensity of tumor sites (*n=3*); I-sR@LNP was used to represent I-Cy5-sR@LNP. I-sR@MLNP was used to represent I-Cy5-sR@MLNP.


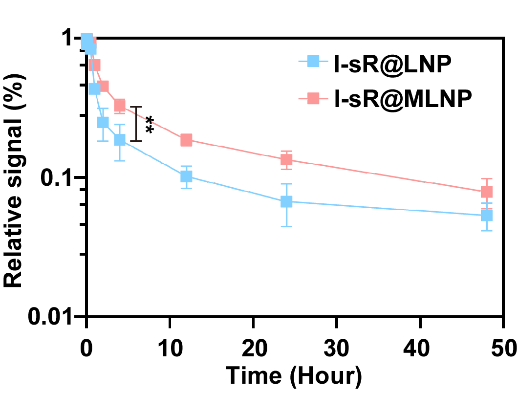


**Fig. S22.** Cy5-labeled I-sR@LNP and I-sR@MLNP were injected through tail vein of the mice. Fluorescence was measured at different time points as indicated (*n* = 3). I-sR@LNP was used to represent I-Cy5-sR@LNP. I-sR@MLNP was used to represent I-Cy5-sR@MLNP.


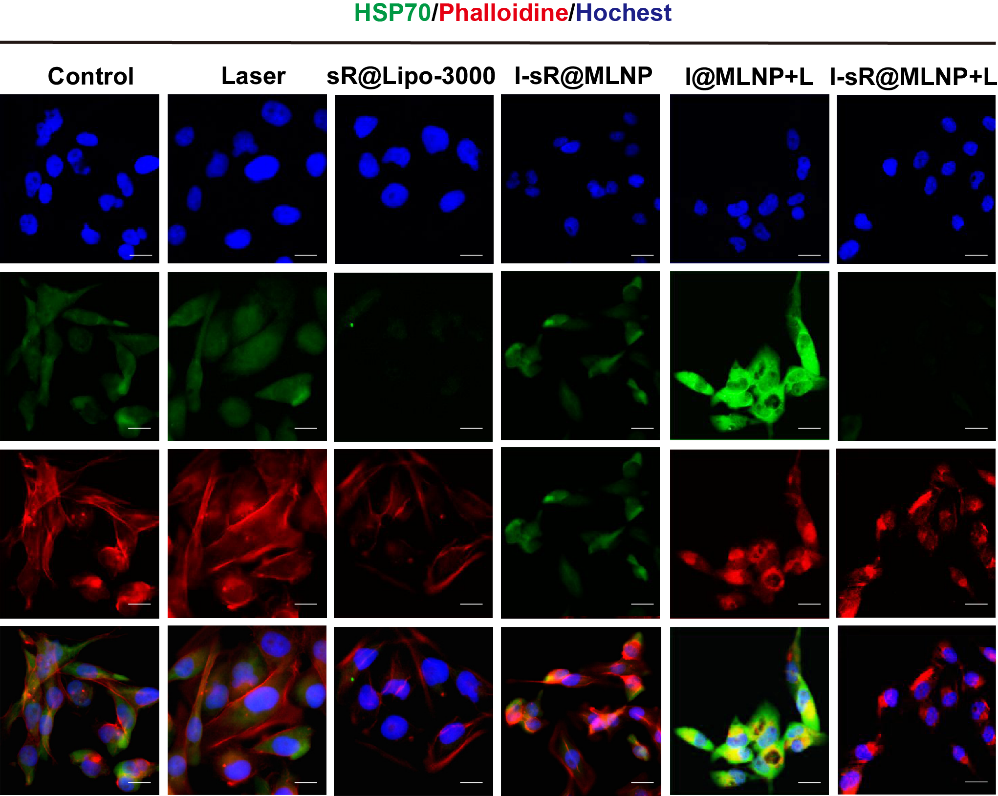


**Fig. S23.** The CLSM images in MDA-MB-231 cells with different approaches. Note: The concentration of ICG-II in various LNPs is 50 μg/mL. The laser is 785 nm and the density is 0.6 W/cm^2^. Scale bar: 50 μm.


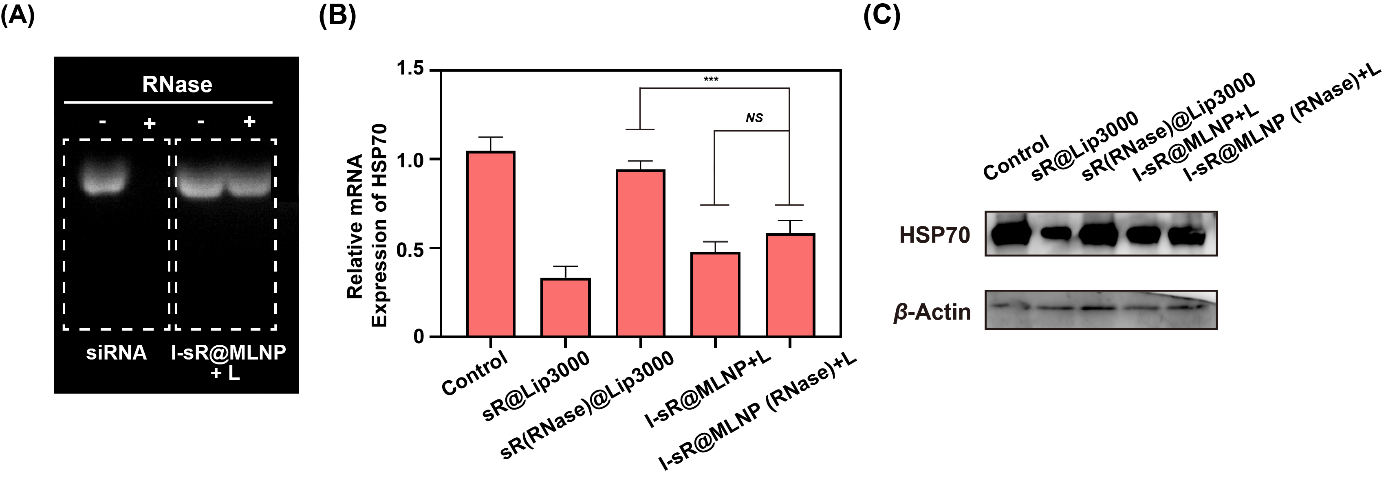


**Fig. S24.** (A) The stability of I-sR@MLNP in RNase; (B) The relative mRNA expression of HSP70 by qPCR; (C) The WB analysis on the HSP70 of MDA-MB-231 treated with different formulations.


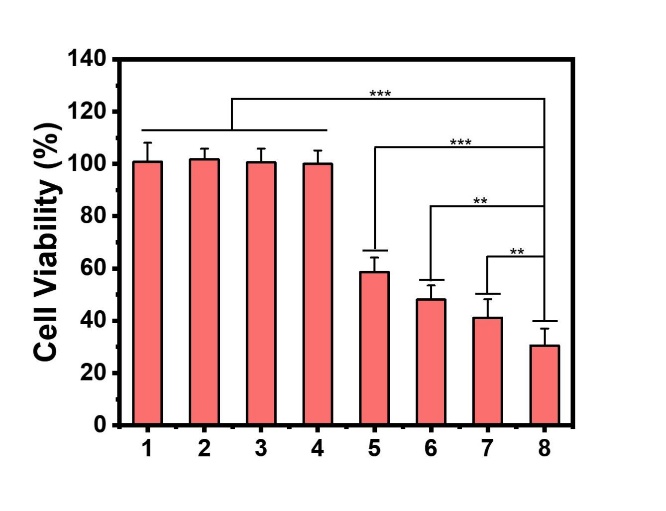


**Fig. S25.** The cell viability with different treatments under single laser (*n=3*). 1 to 8 respectively represent control, Laser, I-sR@LNP, I-sR@MLNP, I@LNP with Laser, I@MLNP with Laser, I-sR@LNP with Laser, and I-sR@MLNP with Laser. I@LNP, I@MLNP, I-sR@LNP and I-sR@MLNP contain 50 μg/mL ICG-II. I-sR@LNP and I-sR@MLNP contain 100 nM siRNA at the same time.


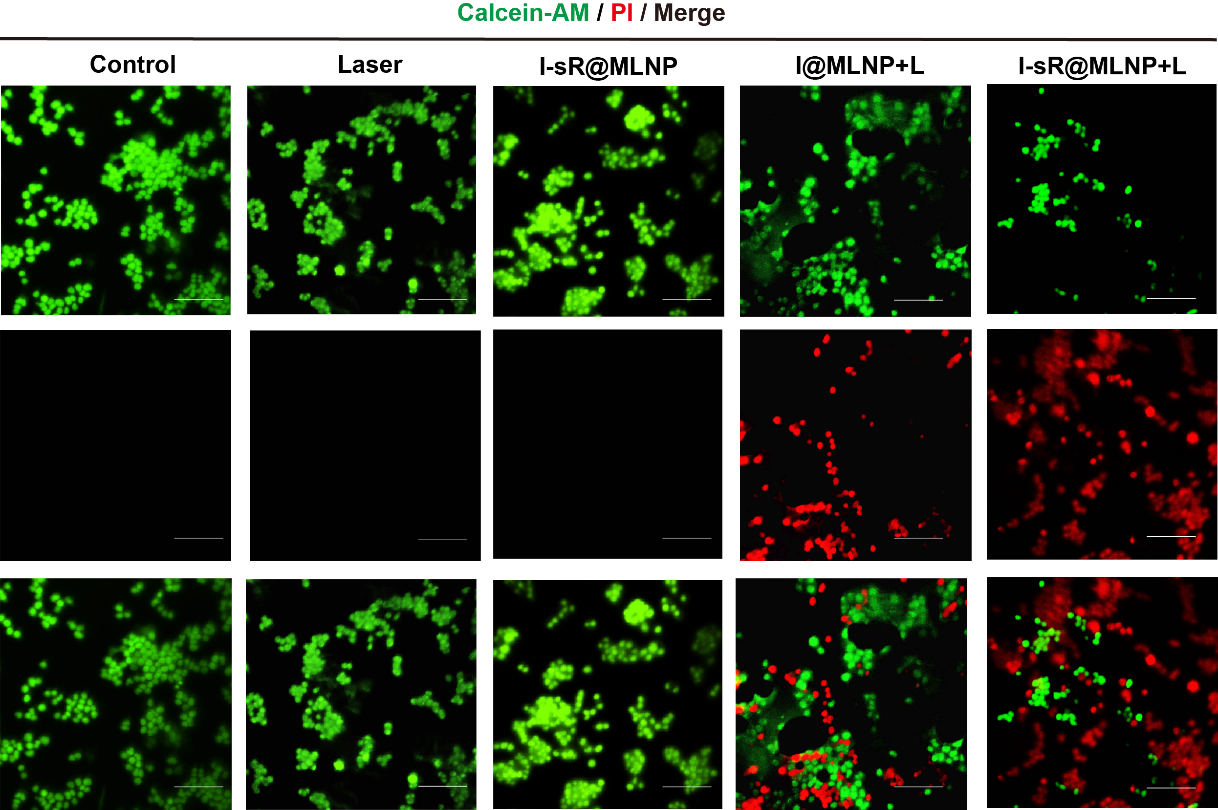


**Fig. S26.** The Live and dead cell staining in MDA-MB-231 cells with different approaches. Note: The concentration of ICG-II in various LNPs is 50 μg/mL. The laser is 785 nm and the density is 0.6 W/cm^2^. Scale bar: 100 μm.


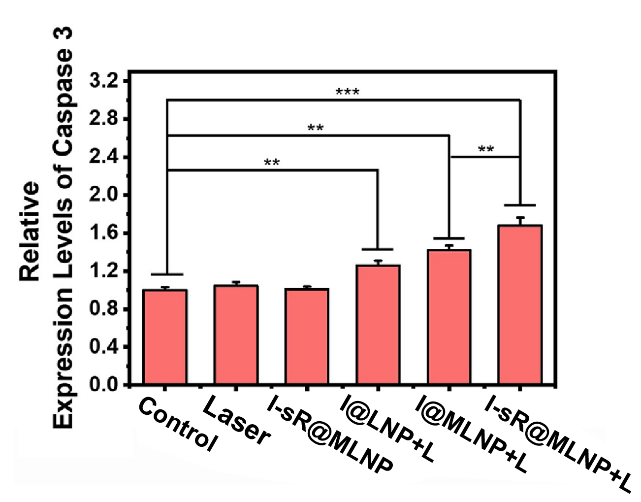


**Fig. S27.** The semi-quantitative analysis of WB of Caspase 3 level (*n=3*).


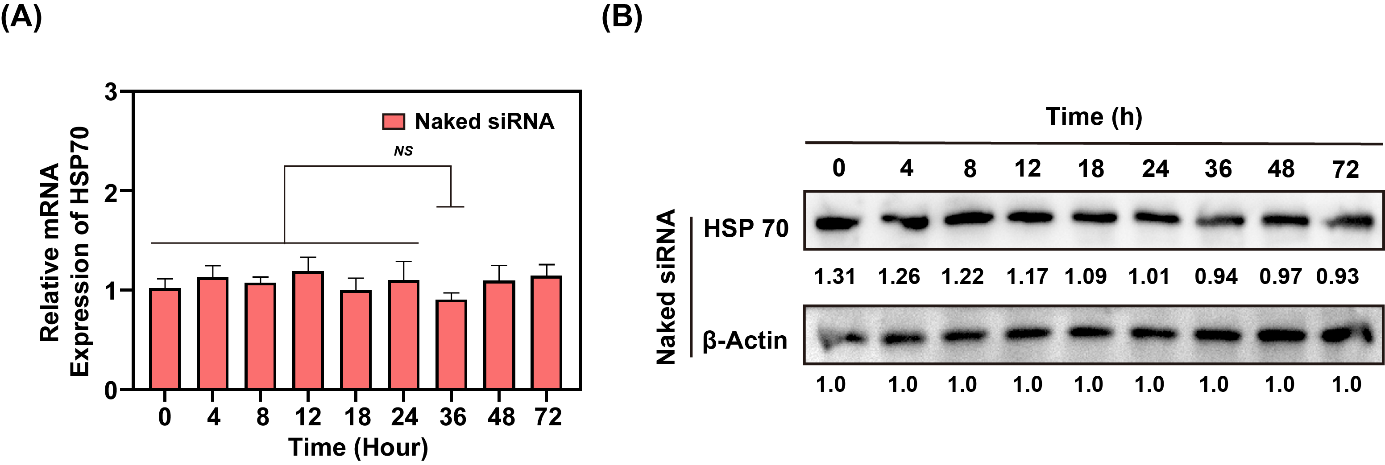


**Fig. S28.** A) The relative HSP70 mRNA levels in MDA-MB-231 cells after incubation with naked siRNA (*n*=3). B) The relative HSP70 protein levels in MDA-MB-231 cells after incubation with naked siRNA.


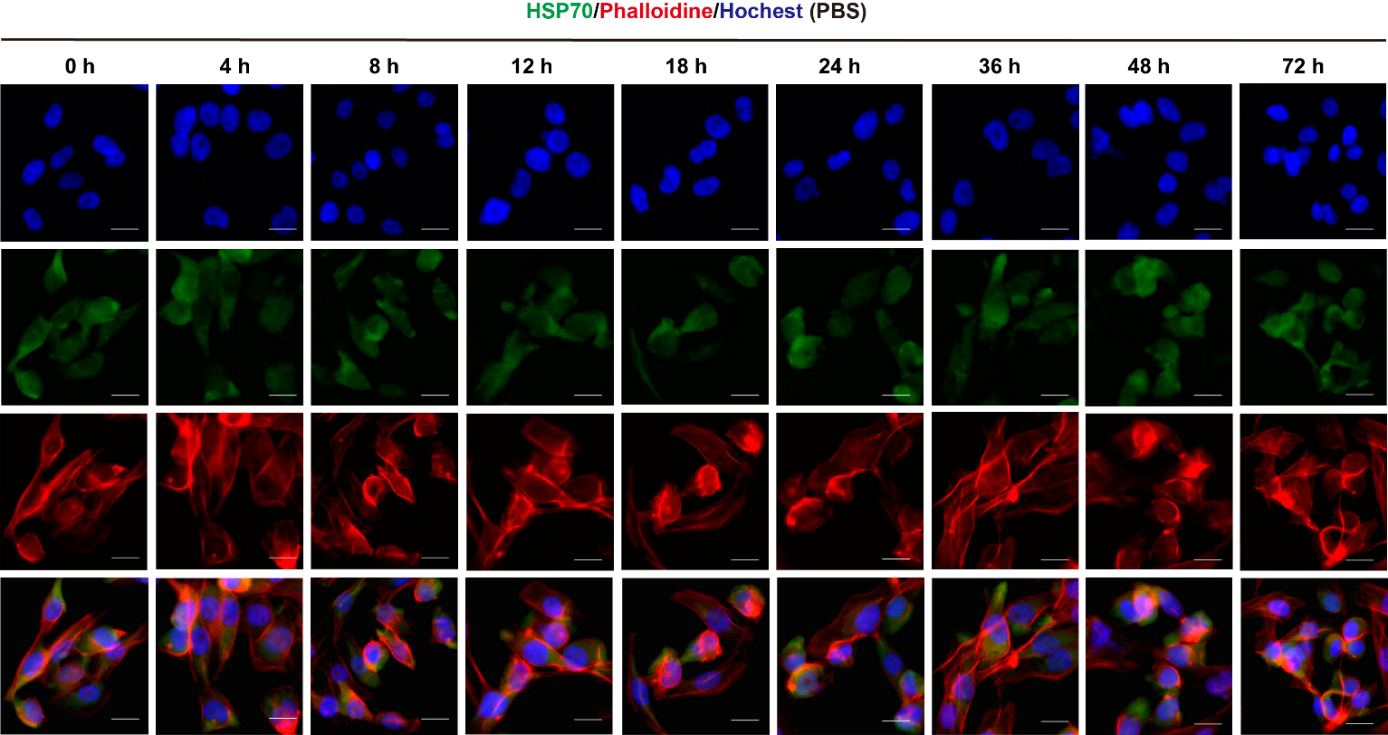


**Fig. S29.** The CLSM images in MDA-MB-231 cells treated with PBS at different times. Note: The concentration of ICG-II in various LNPs is 50 μg/mL. Scale bar: 50 μm.


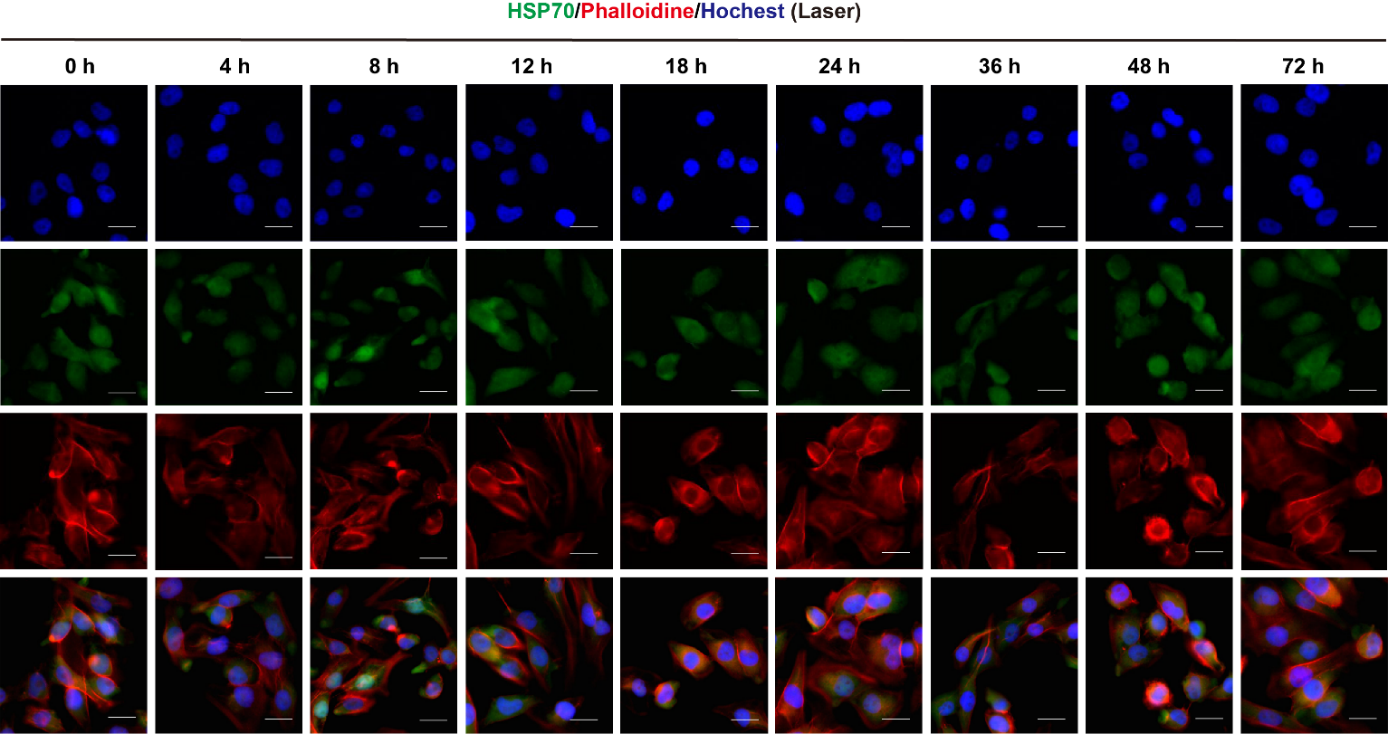


**Fig. S30.** The CLSM images in MDA-MB-231 cells under laser at different times. The laser is 785 nm and the density is 0.6 W/cm^2^. Scale bar: 50 μm.


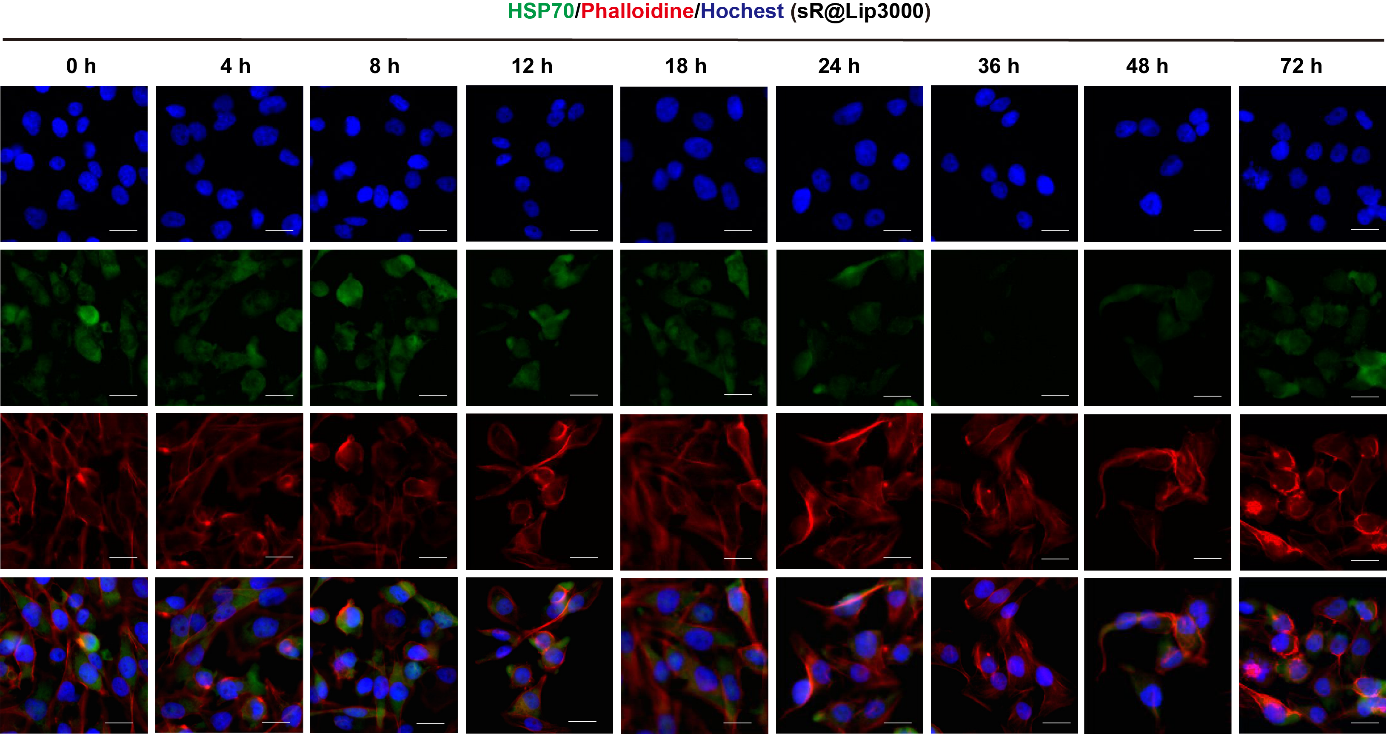


**Fig. S31.** The CLSM images in MDA-MB-231 cells treated with siRNA-Lipo3000 at different times. Scale bar: 50 μm.


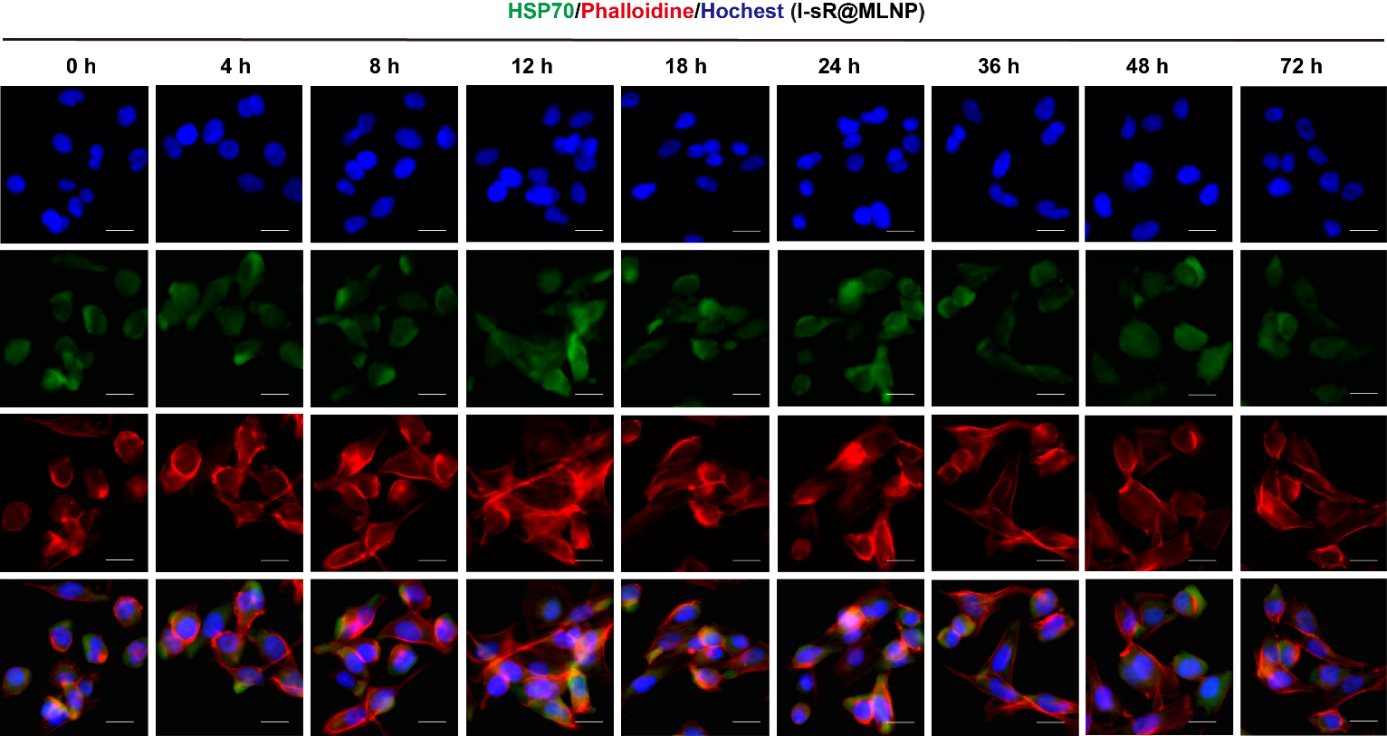


**Fig. S32.** The CLSM images in MDA-MB-231 cells treated with I-sR@MLNP at different times. Note: The concentration of ICG-II in various LNPs is 50 μg/mL. Scale bar: 50 μm.


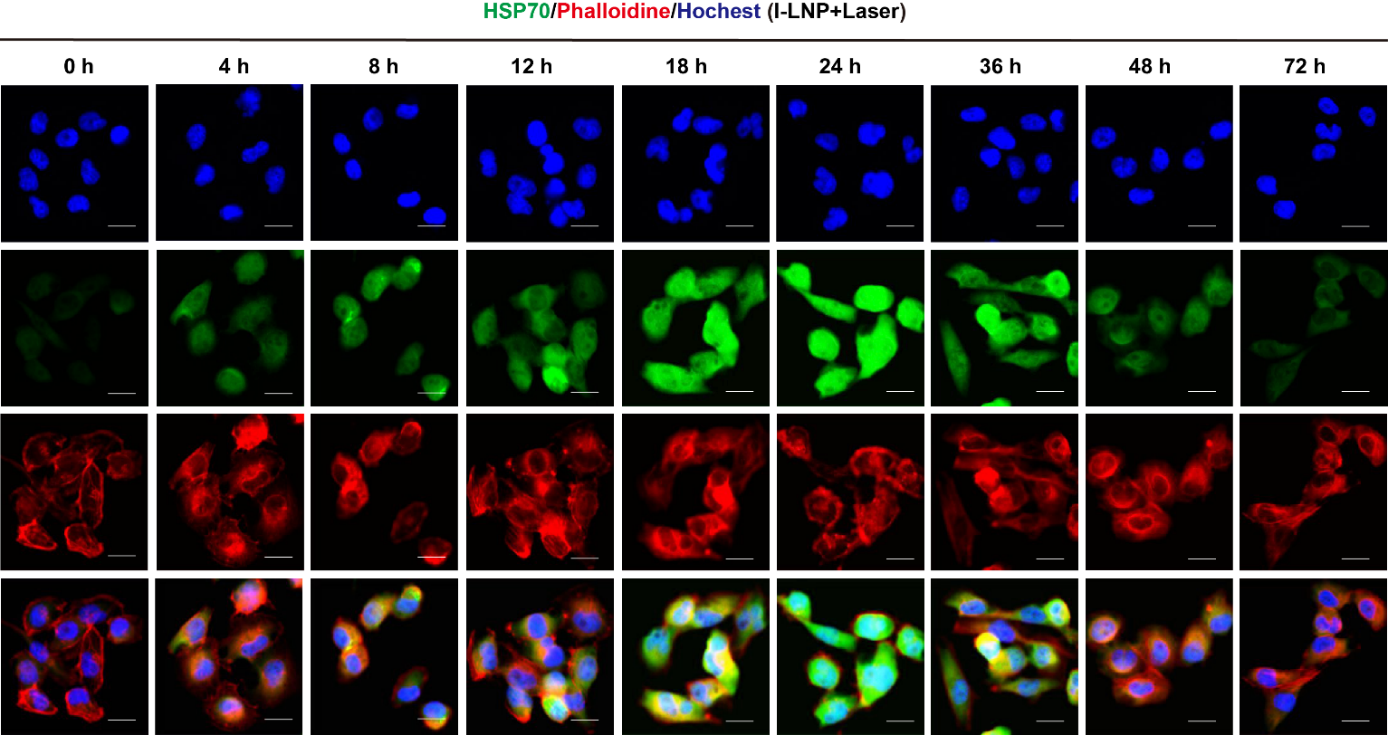


**Fig. S33.** The CLSM images in MDA-MB-231 cells treated with I-LNP under Laser at different times. Note: The concentration of ICG-II in various LNPs is 50 μg/mL. The laser is 785 nm and the density is 0.6 W/cm^2^. Scale bar: 50 μm.


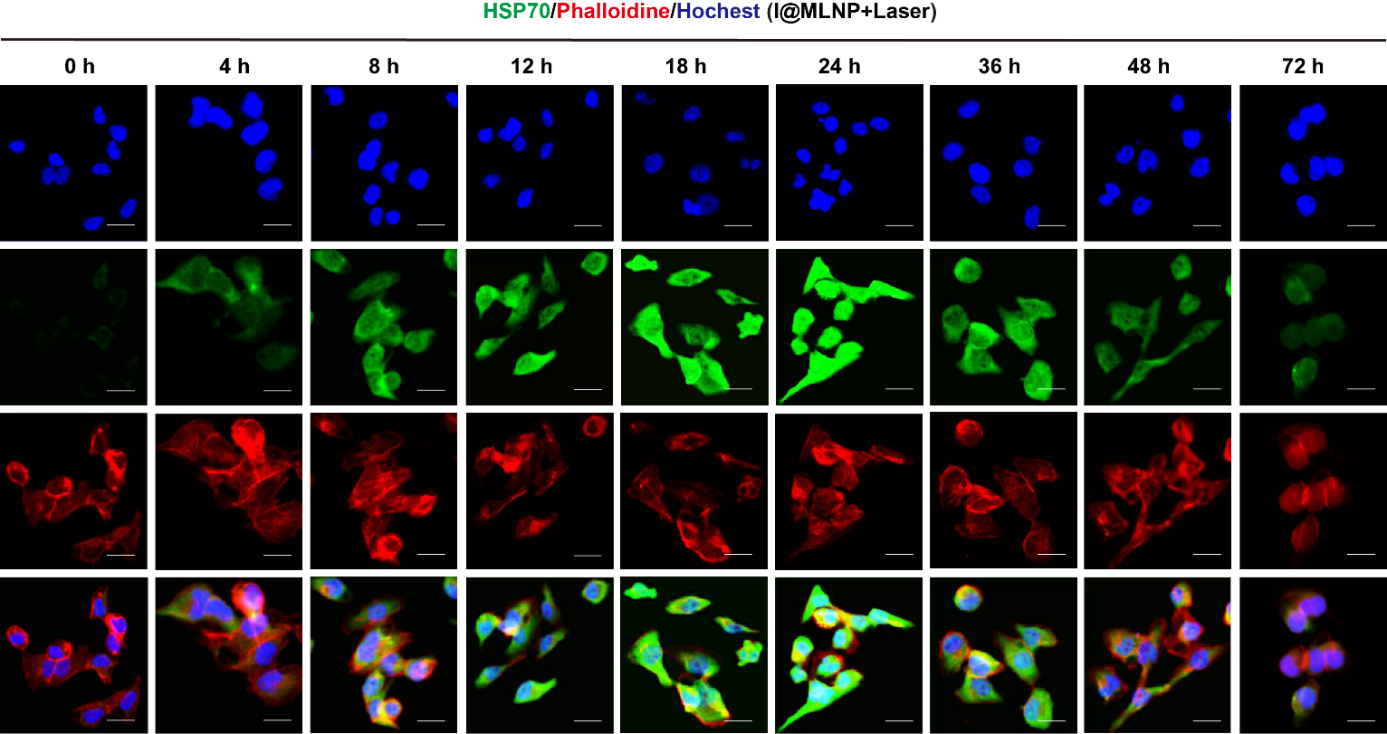


**Fig. S34.** The CLSM images in MDA-MB-231 cells treated with I@MLNP under Laser at different times. Note: The concentration of ICG-II in various LNPs is 50 μg/mL. The laser is 785 nm and the density is 0.6 W/cm^2^. Scale bar: 50 μm.


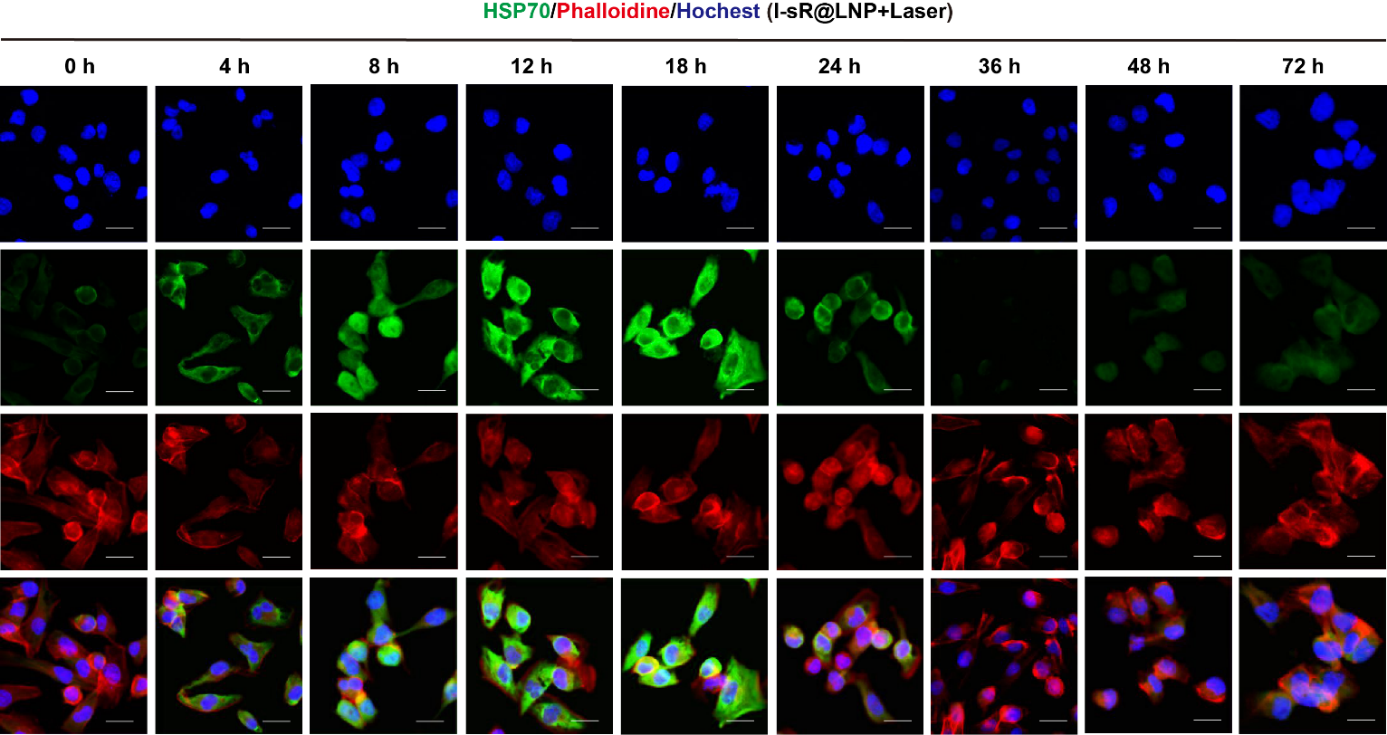


**Fig. S35.** The CLSM images in MDA-MB-231 cells treated with I-sR@LNP under Laser at different times. Note: The concentration of ICG-II in various LNPs is 50 μg/mL. The laser is 785 nm and the density is 0.6 W/cm^2^. Scale bar: 50 μm.


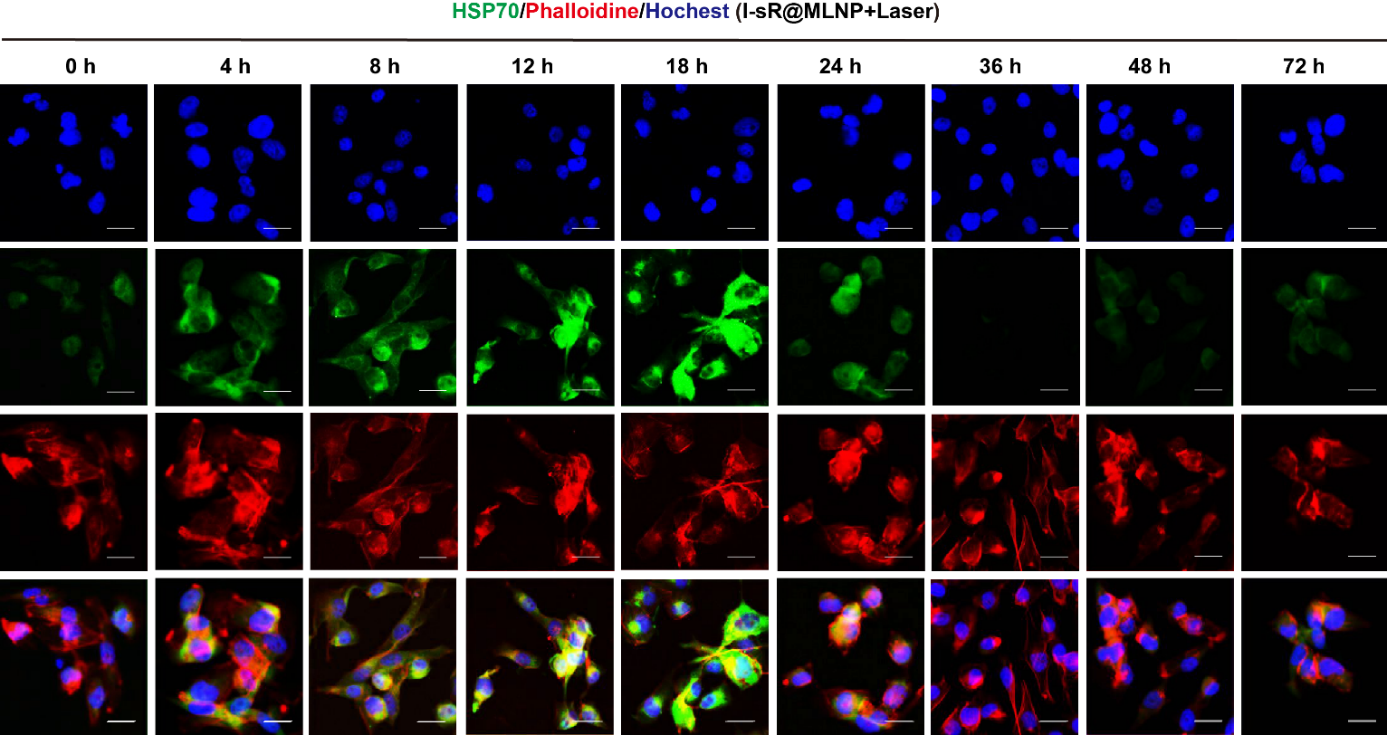


**Fig. S36.** The CLSM images in MDA-MB-231 cells treated with I-sR@MLNP under Laser at different times. Note: The concentration of ICG-II in various LNPs is 50 μg/mL. The laser is 785 nm and the density is 0.6 W/cm^2^. Scale bar: 50 μm.


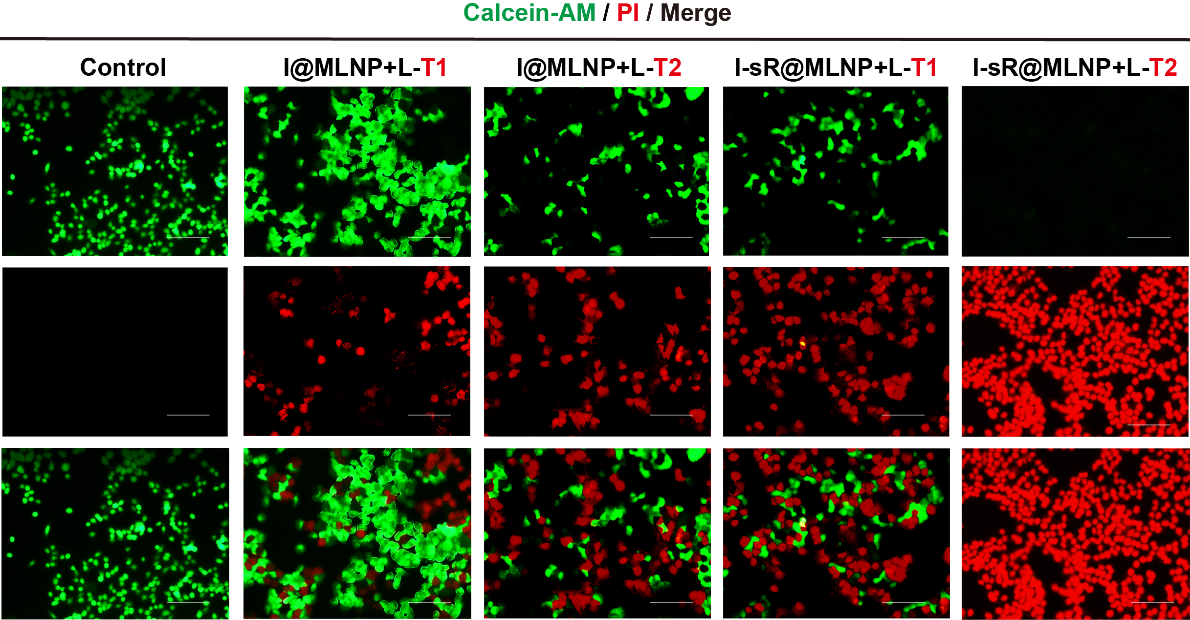


**Fig. S37.** The Live and dead cell staining in MDA-MB-231 cells with different approaches. Note: The interval is 36 hours. The concentration of ICG-II in various MLNPs is 50 μg/mL. The laser is 785 nm and the density is 0.6 W/cm^2^. Scale bar: 100 μm.


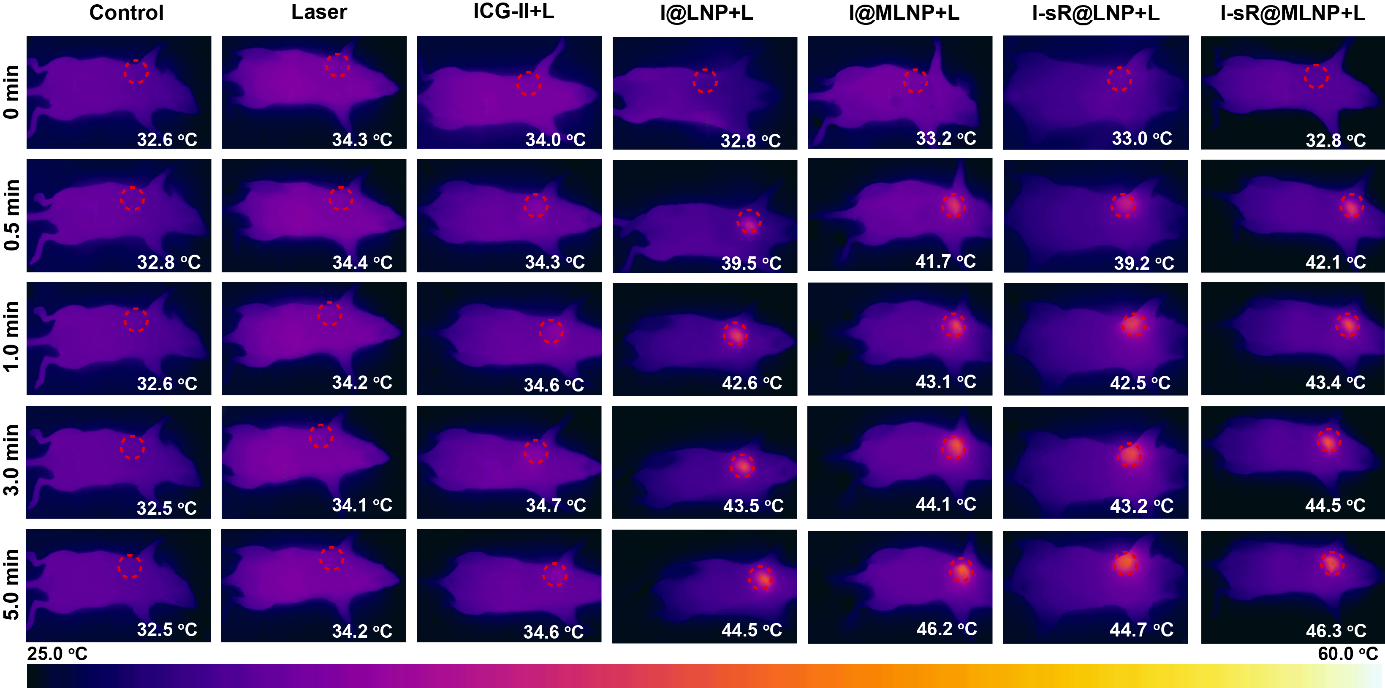


**Fig. S38.** The IR imaging photograph with different treatments.


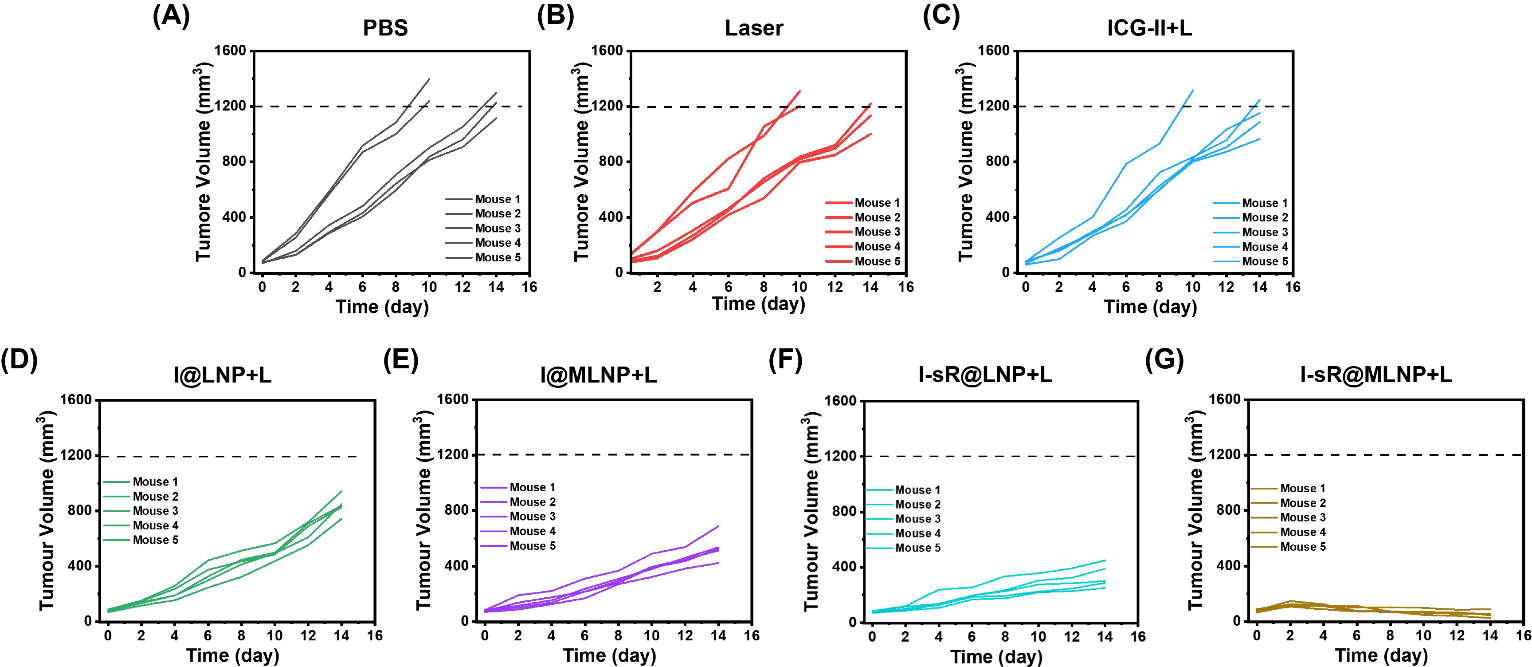


**Fig. S39.** (A) The tumor volume injected with PBS through the tail vein. (B) The tumor volume injected with PBS through the tail vein under a laser. (C) The tumor volume injected with ICG-II through the tail vein under a laser; (D) The tumor volume injected with I-@LNP through the tail vein under a laser. (E) The tumor volume injected with I-@MLNP through the tail vein under a laser. (F) The tumor volume injected with I-sR@LNP through the tail vein under a laser. (G) The tumor volume injected with I-sR@MLNP through the tail vein under a laser. The laser is 785 nm and the density is 0.6 W/cm^2^.


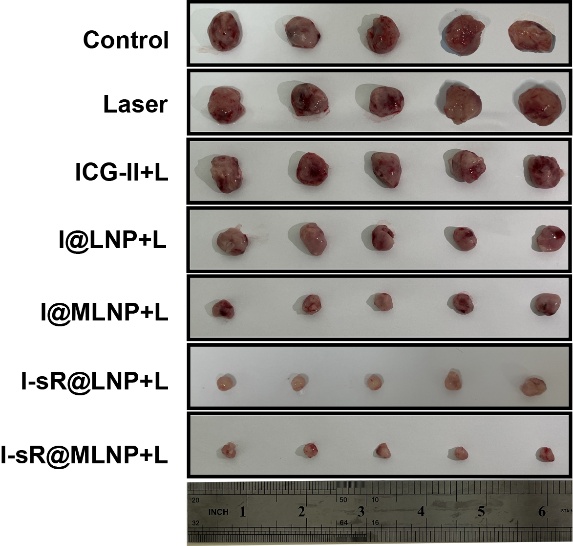


**Fig. S40.** A photograph of the excised tumors from all groups at 14 days after treatment. The laser is 785 nm and the density is 0.6 W/cm^2^.


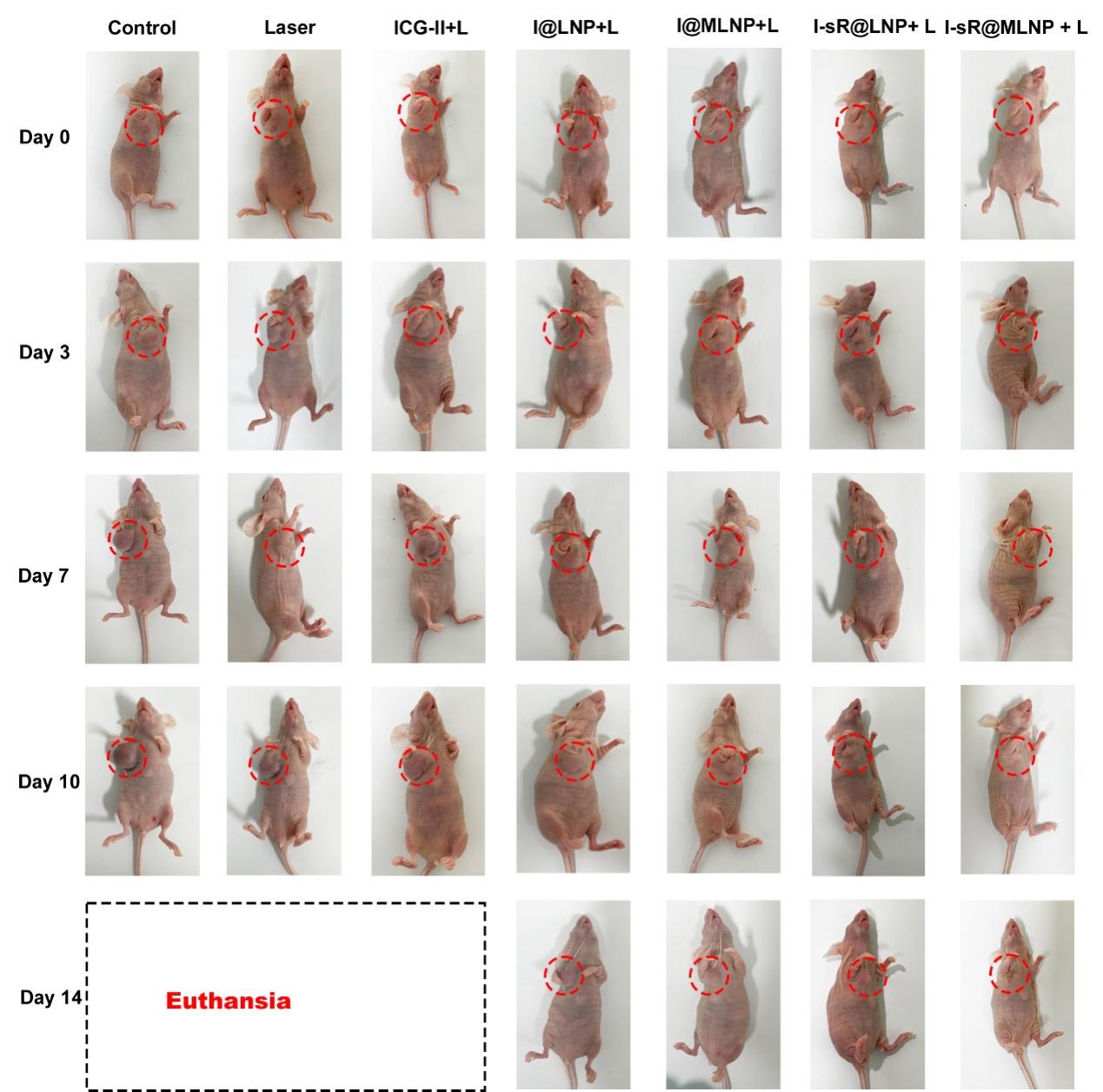


**Fig. S41.** The Representative time-dependent photographs of mice injected with different drugs through the tail vein for dual-phase MPTT.


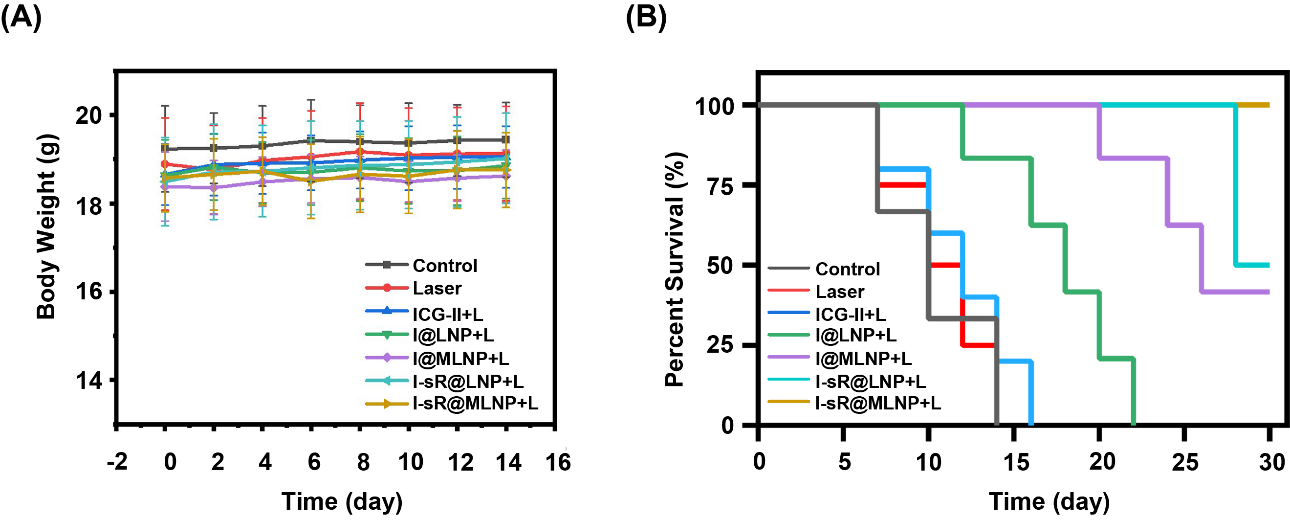


**Fig. S42.** (A) The body weight of different groups (*n=5*). (B) The survival rate of mice in different groups after 14 days (*n=5*).


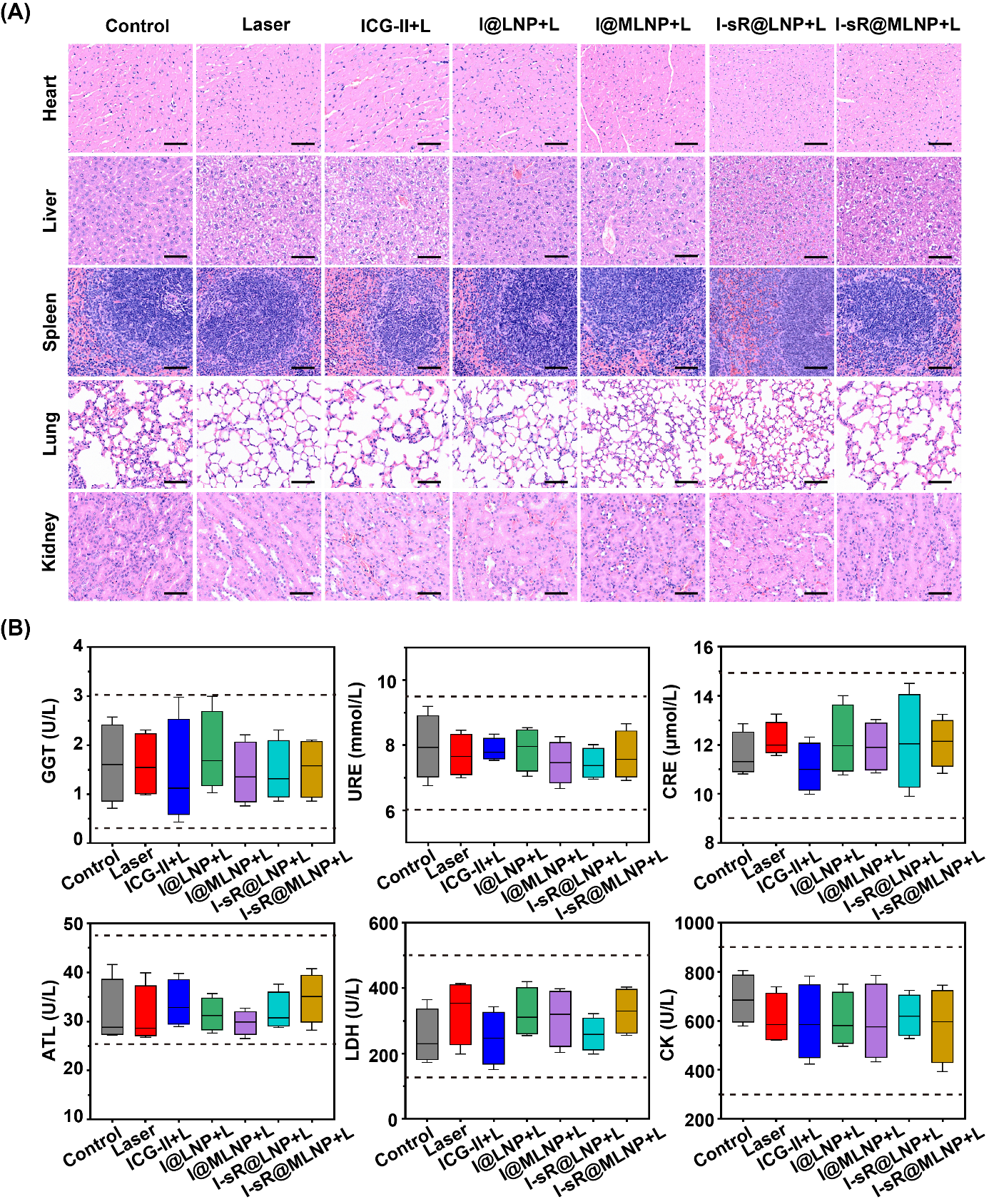


**Fig. S43.** (A) The H&E staining of different organs after 48-hour post-injection. Note: Scale bars: 100 μm. (B) The data analysis of blood biochemical indexes in different groups (*n=5*).


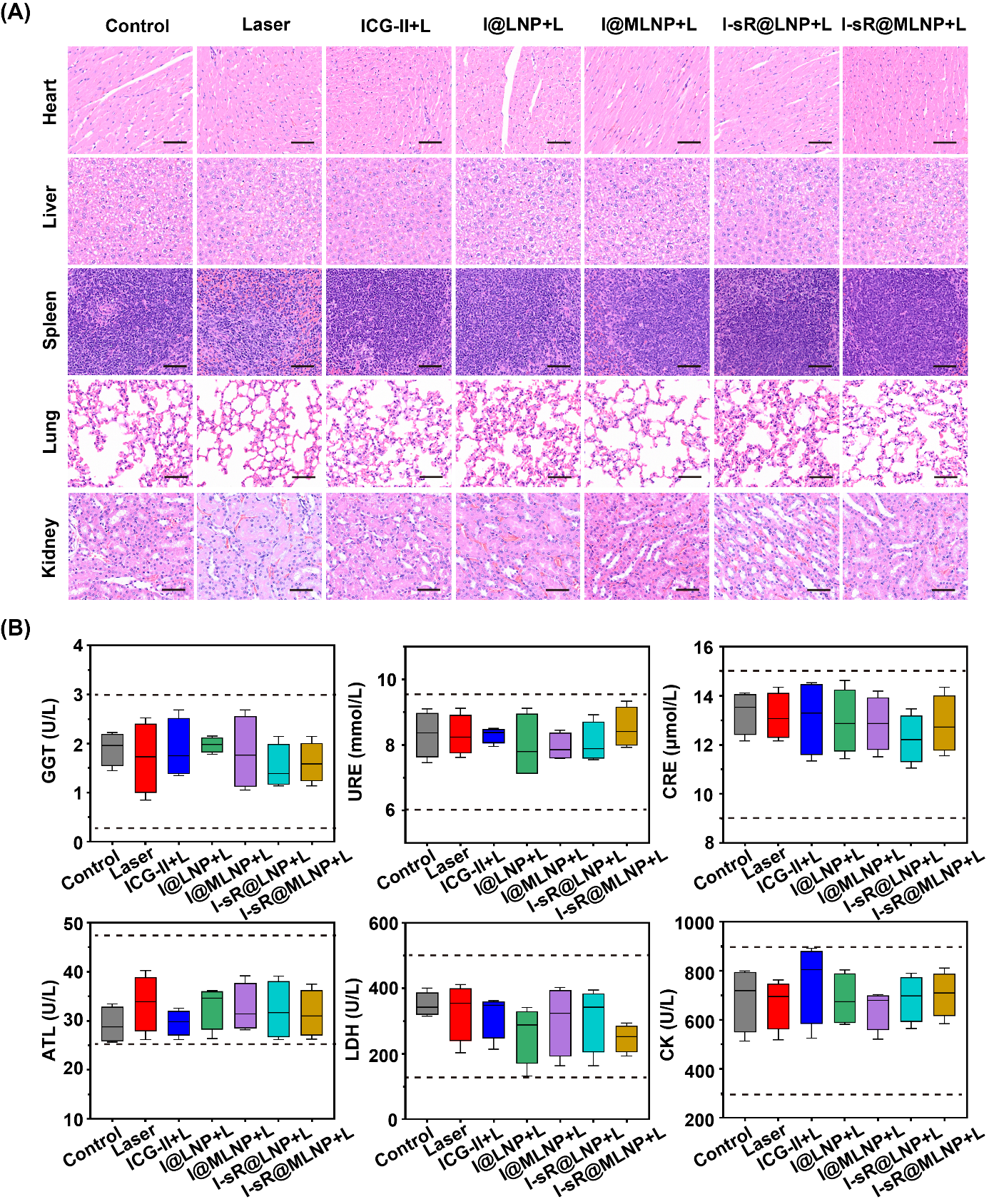


**Fig. S44.** (A) The H&E staining of different organs after 20-day post-injection. Note: Scale bars: 100 μm. (B) The data analysis of blood biochemical indexes in different groups (*n=5*).

**Table S1.** The summary of reported nanoparticle.

| **Compound** | **λex (nm)** | **λem (nm)** | **PTCE (%)** | **Detection dosage** | **Laser condition** | **Ref.** |  |
| --- | --- | --- | --- | --- | --- | --- | --- |
| I-siRNA-MLNP | 780 | ND | 95.4 | 80 μg/mL | 785 nm 1 W/cm^2^ | This work | |
| C6T1 NPs | 750 | ND | 89.3 | 10 μg/mL | 808 nm 0.75 W/cm^2^ | ^[29]^ | |
| tmf-BDP NPs | 810 | ND | 88 | 13.3 μg/mL | 808 nm 0.3 W/cm^2^ | ^[30]^ | |
| TA1 NPs | 730 | ND | 84.3 | 50 μg/mL | 808 nm 2 W/cm^2^ | ^[31]^ | |
| F8-PEG NPs | 750 | ND | 81.7 | 50 μg/mL | 808 nm 1 W/cm^2^ | ^[32]^ | |
| NMS2 NPs | 953 | ND | 75 | 25 μg/mL | 808 nm 1 W/cm^2^ | ^[33]^ | |
| 2DMTT-BBTD NPs | 736 | ND | 74.8 | 283.2 mg/mL | 808 nm 0.8 W/cm^2^ | ^[34]^ | |
